# Preparation and X-ray Structural Study of Dibenzobromolium and Dibenzochlorolium Derivatives

**DOI:** 10.1021/acsomega.3c07512

**Published:** 2024-01-03

**Authors:** Christopher D. Huss, Akira Yoshimura, Gregory T. Rohde, Irina A. Mironova, Pavel S. Postnikov, Mekhman S. Yusubov, Akio Saito, Viktor V. Zhdankin

**Affiliations:** † Department of Chemistry and Biochemistry, University of Minnesota Duluth, Duluth, Minnesota 55812, United States; ‡ Faculty of Pharmaceutical Sciences, Aomori University, 2-3-1 Kobata, Aomori 030-0943, Japan; § Marshall School, Duluth, Minnesota 55811, United States; ∥ Research School of Chemistry and Applied Biomedical Sciences, The Tomsk Polytechnic University, Tomsk 634050, Russia; ⊥ Department of Solid-State Engineering, University of Chemistry and Technology, Prague 16628, Czech Republic; # Division of Applied Chemistry, Institute of Engineering, Tokyo University of Agriculture and Technology, 2-23-16 Naka-cho, Koganei, Tokyo 184-8588, Japan

## Abstract

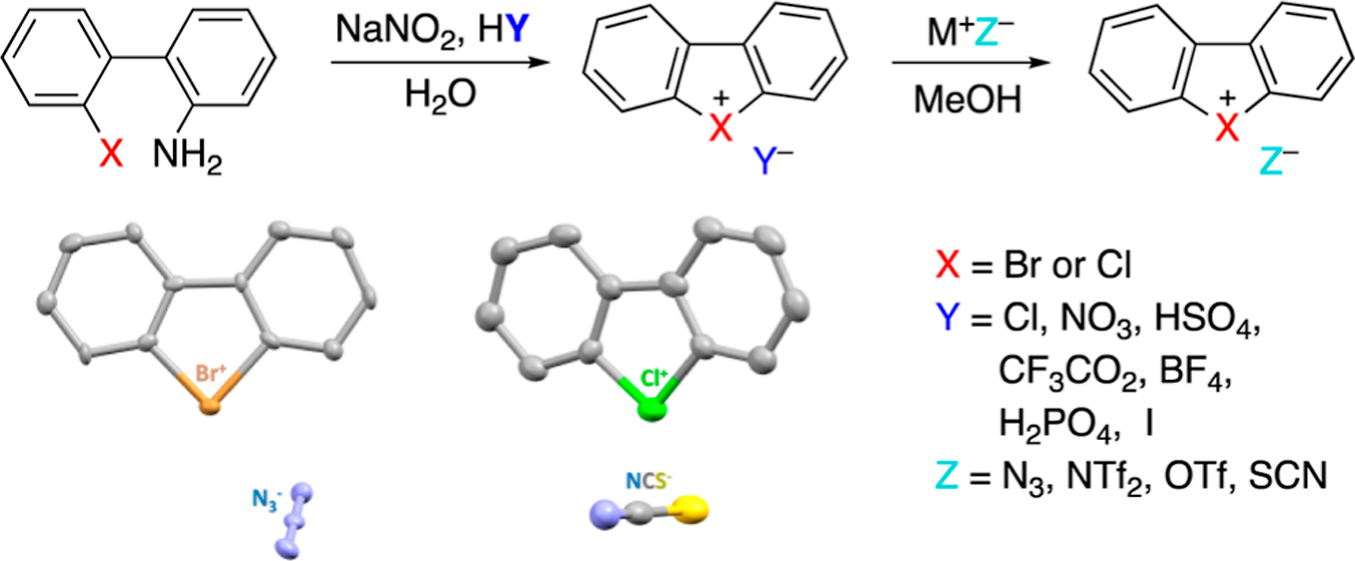

Various five-membered cyclic dibenzobromolium salts (dibenzo[*b*,*d*]bromol-5-ium chloride, nitrate, hydrogen sulfate, dihydrogen phosphate, trifluoroacetate, and tetrafluoroborate) were prepared by diazotization–cyclization of 2′-bromo-[1,1′-biphenyl]-2-amine in solution of appropriate acids. The chlorolium analogues (iodide, trifluoroacetate, and tetrafluoroborate) were obtained by a similar procedure. Additional dibenzohalolium derivatives (dibenzo[*b*,*d*]bromol-5-ium and dibenzo[*b*,*d*]chlorol-5-ium azides, bis(trifluoromethanesulfonyl)imidates, thiocyanates, and trifluoromethanesulfonates) were prepared by anion exchange. Structures of ten of these dibenzohalolium derivatives were established by X-ray analysis. Bond distances and angles for the halogen atoms in different dibenzohalolium derivatives were summarized and discussed.

## Introduction

Cyclic diaryliodonium salts, which are mainly represented by the five-membered dibenzoiodolium derivatives, have found wide application in organic synthesis and pharmaceutical research.^1^ The analogous dibenzobromolium and dibenzochlorolium derivatives have been less investigated despite been known since 1952.^2^ Very recently, several important synthetic applications of dibenzobromolium salts have been reported, such as halogen-bonding organocatalysts,^3^ reagents for cross-coupling reactions with anionic nucleophiles,^4^ as aryne precursors in cycloaddition reactions,^5^ and reagents for one pot synthesis of benzo[*c*]-cinnolines and azobenzenes.^6^ Synthetic utilization of dibenzochlorolium salts as aryne precursors has recently been reported.^7^ In general, arylchloronium and arylbromonium derivatives have higher reactivity toward nucleophilic organic substrates compared to aryliodonium salts, which justifies synthetic interest in these compounds. It should be noted that previously prepared dibenzobromolium and dibenzochlorolium derivatives were limited to tetrafluoroborate,^3a,4,9^ hexafluorophosphate,^9^ iodide,^2a^ chloride,^2a^ triflate,^3a,5,7^ tosylate,^6^ and tetraarylborate^3a^ salts. It is well known that there is a significant secondary bonding between the iodine atom in aryliodonium salt and the counteranion, which has a strong effect on the reactivity of aryliodonium species.^1^ It can be expected that the nature of the counterion in dibenzobromolium and dibenzochlorolium derivatives also affects the structure and reactivity of these important reagents. In the present paper, we report convenient experimental procedures for the preparation of dibenzobromolium and dibenzochlorolium salts with various anions and X-ray structural analysis of these compounds.

## Results and Discussion

**Scheme 1:**
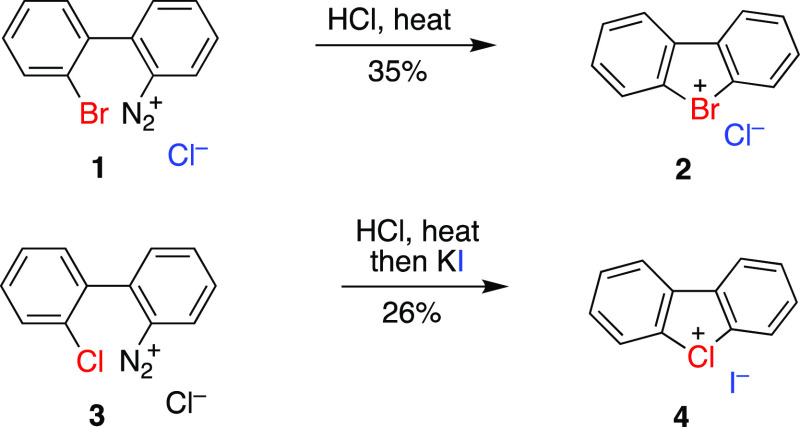
Classic Synthetic Approach to Dibenzobromolium and Dibenzochlorolium Salts^2a,9^

A typical synthetic approach to dibenzobromolium (**2**) and dibenzochlorolium (**4**) salts involves thermal decomposition of the corresponding diazonium salts **1** and **3** with a neighboring bromine or chlorine atom (Scheme 1).^2a,9^ This approach requires the isolation of potentially explosive diazonium salts and affords dibenzohalolium salts **2** and **4** in a relatively low yield.

**Scheme 2:**
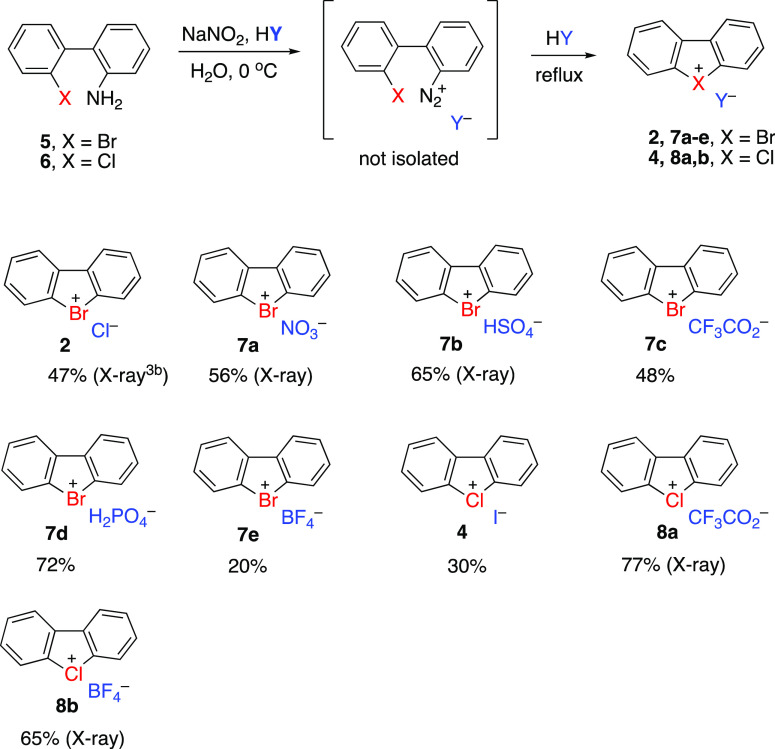
Synthesis of Dibenzobromolium and Dibenzochlorolium Salts of Strong Acids

We have developed a simple and convenient experimental procedure for direct preparation of several dibenzobromolium and dibenzochlorolium salts directly from 2′-bromo-[1,1′-biphenyl]-2-amine **5** or 2′-chloro-[1,1′-biphenyl]-2-amine **6** by the diazotization–cyclization sequence in the presence of appropriate acids (Scheme 2). In this procedure, the corresponding diazonium salts were generated from amines **5** and **6** and sodium nitrite in aqueous solution in the presence of strong acids HY at 0 °C. The resulting solution was then heated to reflux until completion of gas evolution, cooled to 0 °C, and white precipitates of products were collected by filtration. This straightforward procedure allows the preparation of products **2**, **4**, **7**, and **8** in better yields (up to 77%) and with wide selection of counteranions.

**Scheme 3:**
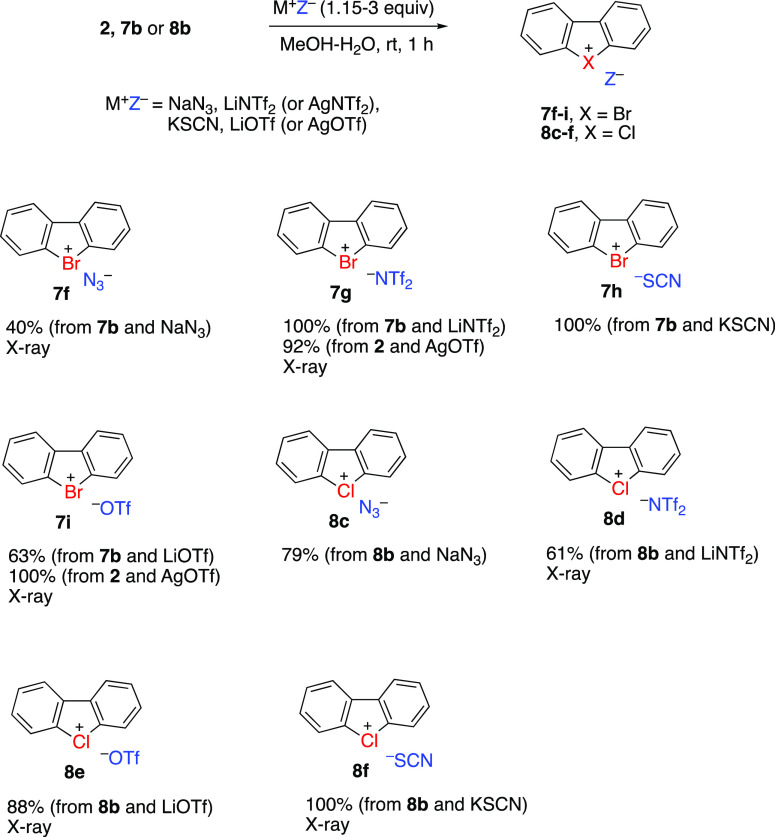
Preparation of Dibenzobromolium and Dibenzochlorolium Derivatives by Anion Exchange Reactions with Inorganic Salts

Dibenzobromolium and dibenzochlorolium salts **2**, **7b**, and **8b** can be further converted to several other derivatives by anion exchange reactions with appropriate inorganic salts (NaN_3_, LiNTf_2_ or AgNTf_2_, KSCN, LiOTf or AgOTf) in aqueous methanol solution (Scheme 3). It is noteworthy that the anion exchange approach allows the preparation of novel azides **7f**, **8c** and thiocyanates **7h**, **8f** which contain highly nucleophilic anions in their structure. Thermal stability of these derivatives is an unexpected result because arylchloronium and arylbromonium derivatives have exceptionally high reactivity toward nucleophiles, even compared to the highly reactive aryliodonium salts.^8^ Dibenzoiodolium azide and thiocyanate were previously reported.^10^

**Figure 1:**
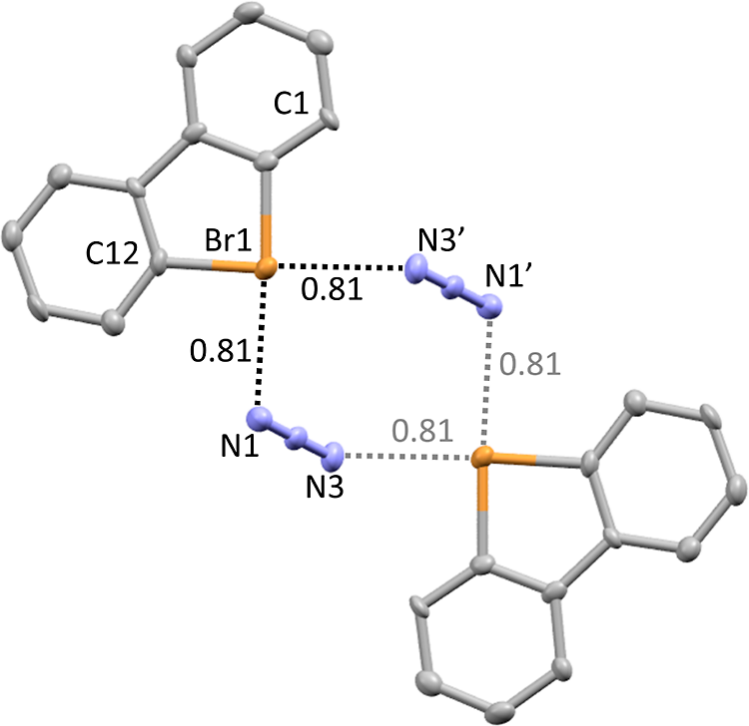
Perspective view of **7f** drawn to the 50% probability level. Hydrogen atoms were removed for clarity. Pairs of molecules and anions form close contacts and are related by an inversion center. Numbers in the figure represent *normalized contact* (Nc, defined here as the observed distance divided by the sum of the Bondi van der Waals radii). Selected distance [Å] and angles [deg] for **7f**: Br1–C1 1.939(5); Br1–C12 1.944(4); C1–Br1–C12 86.7(2); Br1···N1 2.730(4); Br1···N3′ 2.724(3); C1–Br1···N1 177.5(1); and C12–Br1···N3′ 176.7(1).

**Figure 2:**
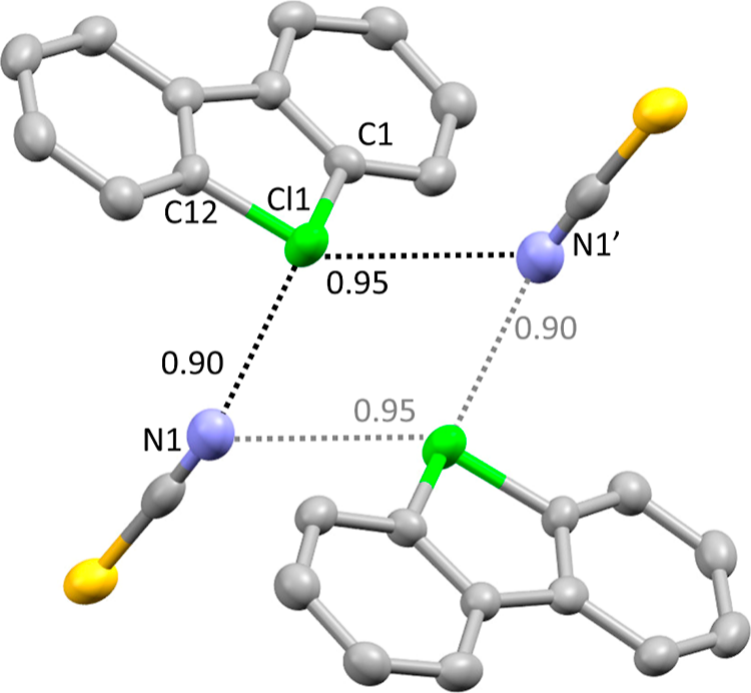
Perspective view of **8f** drawn to the 50% probability level. Hydrogen atoms were removed for clarity. Pairs of molecules and anions form close contacts and are related by an inversion center. Numbers in the figure represent *normalized contact* (Nc, defined here as the observed distance divided by the sum of the Bondi van der Waals radii). Selected distance [Å] and angles [deg] for **8f**: Cl1–C1 1.776(3); Cl1–C12 1.781(4); C1–Cl1–C12 92.1(2); Cl1···N1 2.981(3); Cl1···N3′ 3.133(4); C1–Cl1···N1 152.2(1); and C12–Cl1···N3′ 145.4(1).

All products **7** and **8** were obtained as thermally stable, white, microcrystalline solids, and fully characterized by NMR and IR spectroscopy and ESI mass spectrometry. Structures of ten of these products were established by single-crystal X-ray diffraction. The bond and close contact distances and angles for the halogen atoms in these structures are summarized in Table 1. The two halogen–carbon bonds were found to be symmetric throughout the series of dibenzobromolium and dibenzochlorolium compounds. The average distances of the shorter and longer C–X distance are 1.928 and 1.935 Å for the **7** series and 1.779 and 1.789 Å for the **8** series, respectively. The average difference between the longer and shorter halogen–carbon bonds was found to be within 0.02 Å for the **7** and **8** series compounds. The average C1–Br–C12 and C1–Cl1–C12 bond angles of **7** and **8** are 87 and 92°, respectively, with compounds in each series being within about 1°. The close contact distances are discussed using *normalized contact* (Nc, defined here as the observed distance divided by the sum of the Bondi van der Waals radii). Two close contacts with the halogen atom form the coordination sphere consistent with halogen bonding (XB).^11^ The extended four-coordinate environment of **7** and **8** was consistent with the distorted square planar environment. The average Nc of the two close contacts that completed the four-coordinate geometry was 0.85 for **7** and 0.90 for **8** indicating a moderate attractive interaction, with the **7** series interacting stronger than the **8** series. The stronger interactions of the **7** series were also observed by the close contact forming an angle closer to 180° with the halogen and carbon atom compared to a smaller angle for the **8** series. Structures of **7f** and **8f** are shown as examples of the families of compounds (Table 1, Figures 1, and 2). Additionally, three Br species, **7a**, **7b**, and **7g**, contain bifurcated halogen bonding from a bidentate ligand, but are still consistent with the assignment.^11^ A survey of several dibenzoiodolium salts resulted in an average Nc of ∼0.81 of close contacts with anions of ^–^OTs, ^–^NTf_2_, Cl^–^, ^–^SCN, and N_3_^–^.^7,10,11^ The trend of average Nc for the halogen bonding in the dibenzohalolium series provides the basis of a structure–function hypothesis for the counteranion-reactivity dependence to be I (0.81) > Br (0.85) > Cl (0.90), with the average Nc value in parentheses.

**Table 1:** Bond and Close Contact Distance (Ratios) and Angles of Dibenzobromolium and Dibenzochlorolium Salts

compound (anion)	C1–X (Å)	C12–X (Å)	C1–X–C12 (deg)	Ave Nc^a^ of XB donor	largest angles of XB (deg)
**7a** (NO_3_^–^)	1.927(5)	1.930(5)	86.5(2)	0.86^b^	165.0(2)
					170(1)
**7b** (HSO_4_^–^)	1.924(4)^c^	1.937(5)^c^	87.2(2)	0.85^d^	171.4(2)
					175.4(2)
**7f** (N_3_^–^)	1.939(5)	1.944(4)	86.7(2)	0.81	177.5(1)
					176.7(1)
**7g** (^−^NTf_2_)	1.925(5)	1.925(5)	87.9(3)	0.87^e^	159.6(2), 152.8(3)
				0.85^e^	167.5(4), 158.8(5)
**7i** (^−^OTf)	1.921(9)	1.940(9)	87.5(4)	0.84	173.2(3)
					166.6(3)
**7** average	1.928	1.935	87.2	0.85	N/A
**8a** (CF_3_CO_2_^–^)	1.778(5)^c^	1.788(5)	91.4(3)	0.87	175.5(2)
					161.0(2)
**8b** (BF_4_^–^)	1.776(7)	1.784(7)	91.9(3)	0.89	171.5(3)
					143.9(2)
**8d** (^−^NTf_2_)	1.791(9)^c^	1.80(1)^c^	91.6(4)	0.88	165.4(3)
					158.1(3)
**8e** (^−^OTf)	1.776(8)^c^	1.79(1)^c^	92.0(5)	0.91	173.2(4)
					166.8(4)
**8f** (^−^SCN)	1.776(3)	1.781(4)	92.1(2)	0.93	152.2(1)
					145.4(1)
**8** average	1.779	1.789	91.8	0.90	N/A

a Nc is defined as the observed contact length divided by the sum of the van der Waals radii (Bondi).

b Structure of **7a** also contains a bifurcated halogen bond from a second oxygen atom of the NO_3_^–^ at an Nc of 0.92 and a water oxygen atom at Nc 0.97.

c Bond lengths C1–X and C12–X reported as the short and long bonds in the complex for comparison.

d Structure **7b** has one bifurcated halogen bond with an additional oxygen atom from the HSO_4_^–^ at Nc 0.96.

e Structure **7g** has a *Z*′ = 3 and contains three different close contact coordination spheres. One close contact set contains bifurcated halogen bonds from two oxygen atoms, each pair from separate NTf_2_^–^ counterions with the second oxygen atom from the NTf_2_^–^ at Nc 0.89. A second close contact coordination sphere of approximately square planar with a single nitrogen and oxygen atoms (from separate NTf_2_^–^) as close contacts was also observed. The third cation–anion set in **7g** was badly disordered and not considered in this analysis.

It is interesting to compare the coordination pattern in structures **7f** and **8f** with the previously reported X-ray structures of their iodolium analogues, dibenzoiodolium azide, and thiocyanate.^10^ The coordination spheres of halogen atoms in dibenzoiodolium azide^10^ and dibenzobromolium azide **7f** are very similar to the I···N distance 2.749–2.753 Å (Nc 0.78) and the Br···N distance 2.724–2.730 Å (Nc 0.81), respectively. Both compounds have a dimeric structure with a square-planar geometry of the iodine or bromine centers. The coordination of halogen atoms in dibenzoiodolium thiocyanate^10^ and dibenzochlorolium thiocyanate **8f** is different. Dibenzoiodolium thiocyanate has a polymeric structure formed by two short intermolecular contacts between iodine center and two thiocyanate anions. One of these contacts (∼2.79 Å, Nc 0.79) is between iodine and nitrogen, while the second (∼3.11 Å, Nc 0.82) is between iodine and sulfur atoms.^10^ In contrast, dibenzochlorolium thiocyanate **8f** has a dimeric structure with a square-planar geometry of the chlorine centers coordinated on nitrogen with Cl···N distances 2.981–3.133 Å (Nc 0.90–0.95). No significant chlorine–sulfur interaction is observed in structure **8f**.

## Conclusions

In summary, we have developed a convenient experimental procedure for the preparation of dibenzobromolium and dibenzochlorolium salts by the diazotization–cyclization sequence of the appropriate precursors in the presence of strong acids. Additional dibenzohalolium derivatives, most notably azides and thiocyanates, were prepared by anion exchange. Structures of key products, including the first known examples of stable dibenzobromolium azide and dibenzochlorolium thiocyanate, have been established by single-crystal X-ray diffraction analysis.

## Experimental Section

### General Information

All reactions were performed in open air with a stopper and oven-dried glassware. All commercial reagents were of ACS grade and were used without further purification. NMR spectra were recorded using an Oxford 500 and 300 MHz and a Bruker 400 MHz NMR spectrophotometer (^1^H NMR, ^13^C NMR, and ^19^F NMR). Chemical shifts are reported in parts per million (ppm). Melting points were determined in an open capillary tube with a Mel-Temp II melting point apparatus. Infrared spectra were recorded on a PerkinElmer Spectrum 1600 series FT-IR spectrometer. X-ray crystal analysis was performed by Rigaku RAPID II XRD Image Plate using graphite-monochromated Cu or Mo Kα radiation (λ = 1.54187 or 0.71073 Å) at 125 or 173 K. See the CIF file for more detailed crystallography information. 2′-Bromo-[1,1′-biphenyl]-2-amine **5** and 2′-chloro-[1,1′-biphenyl]-2-amine **6** were prepared according to the reported procedures.^2a^

#### General Procedure for the Synthesis of Cyclic Bromonium Salts **2**, **7a–e** and Cyclic Chloronium Salts **4**, **8a,b**

A solution of 2′-bromo-[1,1′-biphenyl]-2-amine **5** (1.0 equiv) or 2′-chloro-[1,1′-biphenyl]-2-amine **6** (1.0 equiv) in the respective acid (3.0 equiv) was stirred overnight at room temperature. Then, an additional amount of 10% aqueous acid was added and the reaction continued to stir at reflux until the precipitate completely dissolved. After that, the reaction mixture was cooled to 0 °C and sodium nitrate aqueous solution (2 equiv of 0.75 M solution) was added to the reaction mixture and stirred at 0 °C for 1 h. Urea (2.3 equiv) was then added, and the reaction mixture was stirred at 0 °C for another 1 h, then the reaction mixture was heated to reflux until completion of gas evolution. After the completion of the reaction, the reaction mixture was filtrated, washed with a hot 10% solution of the acid used, and the combined solution was concentrated and then kept at 0 °C until the product precipitated out. Then, the precipitate was collected, and after washing with water and ether, the solid was dried in vacuum to obtain final products.

##### Dibenzo[*b*,*d*]bromol-5-ium Chloride **2**(12)

Reaction with **5** (820 mg, 3.3 mmol) and HCl according to the general procedure afforded 376 mg (47%) of product **2**, isolated as an off-white solid: mp 238.1–238.7 °C; IR (KBr) cm^–1^: 3424, 3046, 1630, 1414, 742; ^1^H NMR (400 MHz, DMSO-*d*_6_): δ 8.78 (d, *J* = 8.4 Hz, 2H), 8.57 (d, *J* = 7.6 Hz, 2H), 7.97–7.88 (m, 2H), 7.86–7.79 (m, 2H); ^13^C NMR (100 MHz, DMSO-*d*_6_): δ 137.8, 135.7, 131.9, 131.6, 126.5, 126.5; HRMS (ESI-positive ionization): calcd for C_12_H_8_Br^79^ [M]^+^: 230.9804, found: 230.9818, and calcd for C_12_H_8_Br^81^ [M]^+^: 232.9784, found: 232.9799. The X-ray structure for this compound was previously published.^3b^

##### Dibenzo[*b*,*d*]bromol-5-ium Nitrate **7a**

Reaction with **5** (606 mg, 2.4 mmol) and HNO_3_ according to the general procedure afforded 403 mg (56%) of product **7a**, isolated as a white solid: mp 183.8–185.5 °C; IR (KBr) cm^–1^: 3481, 3051, 1642, 1361, 1017, 745, 640; ^1^H NMR (500 MHz, CD_3_OD): δ 8.50 (d, *J* = 7.5 Hz, 2H), 8.37 (d, *J* = 8.5 Hz, 2H), 7.99–7.94 (m, 2H), 7.87–7.82 (m, 2H); ^13^C NMR (75 MHz, CD_3_OD): δ 136.5, 135.9, 132.0, 131.6, 126.4, 125.1; HRMS (ESI-positive ionization): calcd for C_12_H_8_Br^79^ [M]^+^: 230.9804, found: 230.9784, and calcd for C_12_H_8_Br^81^ [M]^+^: 232.9784, found: 232.9804.

**Figure 3:**
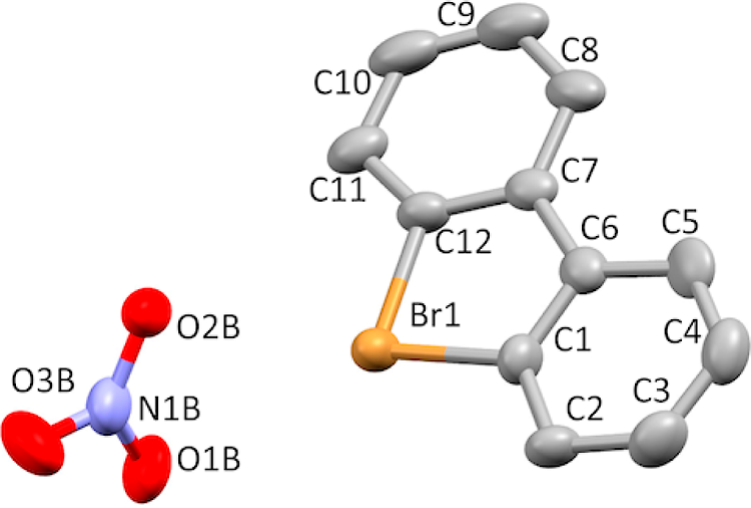
Thermal ellipsoid plot of **7a** drawn to the 50% probability level. One molecule of interest and nitrate ion are displayed for clarity. Water molecules and hydrogen atoms were removed for clarity.

Single crystals of product **7a** suitable for X-ray crystallographic analysis (Figure 3) were obtained by slow crystallization from MeOH–H_2_O solution. X-ray diffraction data for **7a** were collected on a Rigaku RAPID II Image Plate system using graphite-monochromated Cu Kα radiation (λ = 1.54187 Å) at 123 K. The structure was solved by SIR2004 and refined using SHELXL-2014/7. Crystal data for **7a** C_12_H_10_._5_NO_4_._3_Br, monoclinic, space group *P*2(1)/*n*, *a* = 14.3632(3) Å, *b* = 9.5511(2) Å, *c* = 17.5549(12) Å, α = 90°, β = 90.259(6)°, γ = 90°, *V* = 2408.23(18) Å^3^, *Z* = 8, 24,096 reflections measured, 4205 unique reflections, 3179 *I* > 2/*s*(*I*), 399 parameters, 236 restraints; GooF = 1.117, final *R*_1_ = 0.0608, *R*_w_ (all) = 0.0.0803. CCDC 2278336.

##### Dibenzo[*b*,*d*]bromol-5-ium Hydrogen Sulfate **7b**

Reaction with **5** (645 mg, 2.6 mmol) and H_2_SO_4_ according to the general procedure afforded 617 mg (72%) of product **7b**, isolated as a tan solid: mp 125.1–126.6 °C; IR (KBr) cm^–1^: 3044, 1649, 1413, 1288, 1173, 1006, 876, 737, 577; ^1^H NMR (400 MHz, CD_3_CN): δ 8.42 (dd, *J* = 8.0, 1.6 Hz, 2H), 8.30 (dd, *J* = 8.8, 0.8 Hz, 2H), 7.94 (m, 2H), 7.84 (m, 2H); ^13^C NMR (100 MHz, CD_3_CN): δ 137.3, 136.1, 132.9, 132.3, 127.3, 125.7; HRMS (ESI-positive ionization): calcd for C_12_H_8_Br^79^ [M]^+^: 230.9804, found: 230.9815, and calcd for C_12_H_8_Br^81^ [M]^+^: 232.9784, found: 232.9799.

**Figure 4:**
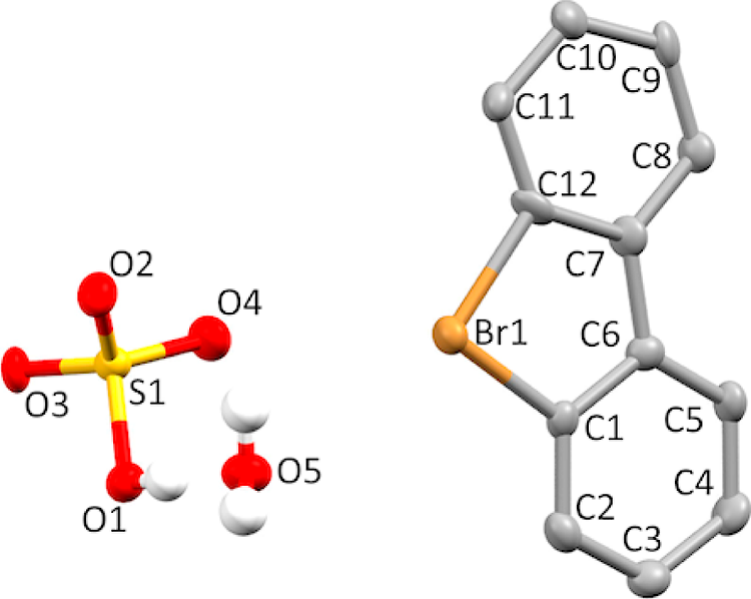
Thermal ellipsoid plot of **7b** drawn to the 50% probability level. Nonoxygen hydrogen atoms were removed for clarity.

Single crystals of product **7b** (as a hydrate) suitable for X-ray crystallographic analysis (Figure 4) were obtained by slow crystallization from MeOH–H_2_O solution. X-ray diffraction data for **7b·H**_**2**_**O** were collected on a Rigaku RAPID II Image Plate system using graphite-monochromated Cu Kα radiation (λ = 1.54187 Å) at 123 K. The structure was solved by SIR2004 and refined using SHELXL-2014/7. Crystal data for **7b·H**_**2**_**O** C_12_H_11_BrO_5_S, triclinic, space group *P*1̅, *a* = 7.12480(10) Å, *b* = 9.8114(2) Å, *c* = 10.5624(7) Å, α = 62.678(4)°, β = 72.726(5)°, γ = 71.746(5)°, *V* = 612.62(5) Å^3^, *Z* = 2, 6535 reflections measured, 2179 unique reflections, 1738 *I* > 2/*s*(*I*), 178 parameters, 27 restraints; GooF = 1.124, final *R*_1_ = 0.0439, *R*_w_ (all) = 0.0563. CCDC 2278333.

##### Dibenzo[*b*,*d*]bromol-5-ium Trifluoroacetate **7c**

Reaction with **5** (568 mg, 2.3 mmol) and TFA according to the general procedure afforded 381 mg (48%) of product **7c**, isolated as an off-white solid: mp 137.2–139.7 °C; IR (KBr) cm^–1^: 3102, 1754, 1417, 1196, 1011, 751; ^1^H NMR (400 MHz, CD_3_CN): δ 8.41 (d, *J* = 8.0 Hz, 2H), 8.31 (d, *J* = 8.4 Hz, 2H), 8.03–7.91 (m, 2H), 7.91–7.78 (m, 2H); ^13^C NMR (100 MHz, CD_3_CN): δ 160.5 (^2^*J*_CF_ = 39.6 Hz), 137.7, 136.5, 133.3, 132.3, 127.6, 126.1, 117.2 (^1^*J*_CF_ = 287.7 Hz); ^19^F NMR (376 MHz, CD_3_CN): δ −76.6; HRMS (ESI-positive ionization): calcd for C_12_H_8_Br^79^ [M]^+^: 230.9804, found: 232.9815, and calcd for C_12_H_8_Br^81^ [M]^+^: 232.9784, found: 232.9801.

##### Dibenzo[*b*,*d*]bromol-5-ium Dihydrogen Phosphate **7d**

Reaction with **5** (631 mg, 2.5 mmol) and H_3_PO_4_ according to the general procedure afforded 606 mg (72%) of product **7d**, isolated as a white solid: mp 223.5–225.6 °C; IR (KBr) cm^–1^: 3095, 2824, 2323, 1640, 1448, 1244, 1102, 984, 759; ^1^H NMR (500 MHz, DMSO-*d*_6_): δ 8.59 (d, *J* = 8.0 Hz, 2H), 8.53 (d, *J* = 8.5 Hz, 2H), 7.97–7.92 (m, 2H), 7.87–7.82 (m, 2H); ^13^C NMR (75 MHz, DMSO-*d*_6_): δ 137.2, 136.0, 132.5, 131.9, 127.0, 126.4; ^31^P NMR (121 MHz, DMSO-*d*_6_): δ 1.5; HRMS (ESI-positive ionization): calcd for C_12_H_8_Br^79^ [M]^+^: 230.9804, found: 230.9815, and calcd for C_12_H_8_Br^81^ [M]^+^: 232.9784, found: 232.9799.

##### Dibenzo[*b*,*d*]bromol-5-ium Tetrafluoroborate **7e**(9)

Reaction with **5** (681 mg, 2.7 mmol) and HBF_4_ according to the general procedure afforded 172 mg (20%) of product **7e**, isolated as a white solid: mp 226.2–228.9 °C; IR (KBr) cm^–1^: 3347, 1634, 1421, 1299, 1040, 736; ^1^H NMR (400 MHz, CD_3_CN): δ 8.41 (d, *J* = 7.6 Hz, 2H), 8.29 (d, *J* = 8.4 Hz, 2H), 7.96 (t, *J* = 7.6 Hz, 2H), 7.90–7.79 (m, 2H); ^13^C NMR (100 MHz, CD_3_CN): δ 137.7, 136.5, 133.4, 132.8, 127.7, 126.1; ^19^F NMR (376 MHz, CD_3_CN): δ −151.2; HRMS (ESI-positive ionization): calcd for C_12_H_8_Br^79^ [M]^+^: 230.9804, found: 230.9816, and calcd for C_12_H_8_Br^81^ [M]^+^: 232.9784, found: 232.9800.

##### Dibenzo[*b*,*d*]chlorol-5-ium Iodide **8a**(2a)

Reaction with **6** (586 mg, 2.9 mmol) and HCl was performed according to the general procedure. After the completion of the reaction, the reaction mixture was filtrated, and the filter was washed with a hot 10% HCl. Then, KI (2390 mg, 14.4 mmol) was added to the solution, and the solution was kept at 0 °C until the solid precipitated out. Then, the solid precipitate was collected, and after washing with water and ether, the solid was dried by vacuum pumping to obtain product **4**. Yield: 268 mg (30%), isolated as a white solid: mp 87.5–88.6 °C; IR (KBr) cm^–1^: 3451, 1626, 1433, 1000, 753; ^1^H NMR (400 MHz, DMSO-*d*_6_): δ 8.68 (d, *J* = 7.6 Hz, 4H), 8.07–7.90 (m, 4H); ^13^C NMR (100 MHz, DMSO-*d*_6_): δ 140.5, 132.5, 132.5, 132.2, 126.0, 123.4; HRMS (ESI-positive ionization): calcd for C_12_H_8_Cl^35^ [M]^+^: 187.0309, found: 187.0323, and calcd for C_12_H_8_Cl^37^ [M]^+^: 189.0282, found: 189.0285.

##### Dibenzo[*b*,*d*]chlorol-5-ium Trifluoroacetate **8a**

*AYO-3039*: Reaction with **6** (590 mg, 2.9 mmol) and TFA according to the general procedure afforded 673 mg (77%) of product **8a**, isolated as an off-white solid: mp 128.3–130.5 °C; IR (KBr) cm^–1^: 3443, 3105, 1764, 1411, 1195, 991, 756; ^1^H NMR (400 MHz, CD_3_CN): δ 8.48 (d, *J* = 8.0 Hz, 2H), 8.42(d, *J* = 8.8 Hz, 2H), 8.04–7.98 (m, 2H), 7.97–7.90 (m, 2H); ^13^C NMR (100 MHz, CD_3_CN): δ 160.1 (^2^*J*_CF_ = 39.1 Hz) 140.3, 133.0, 132.8, 132.8, 126.3, 122.9, 116.9 (^1^*J*_CF_ = 289.1 Hz); ^19^F NMR (377 MHz, CD_3_CN): δ −76.5; HRMS (ESI-positive ionization): calcd for C_12_H_8_Cl^35^ [M]^+^: 187.0309, found: 187.0327, and calcd for C_12_H_8_Cl^37^ [M]^+^: 189.0282, found: 189.0291.

**Figure 5:**
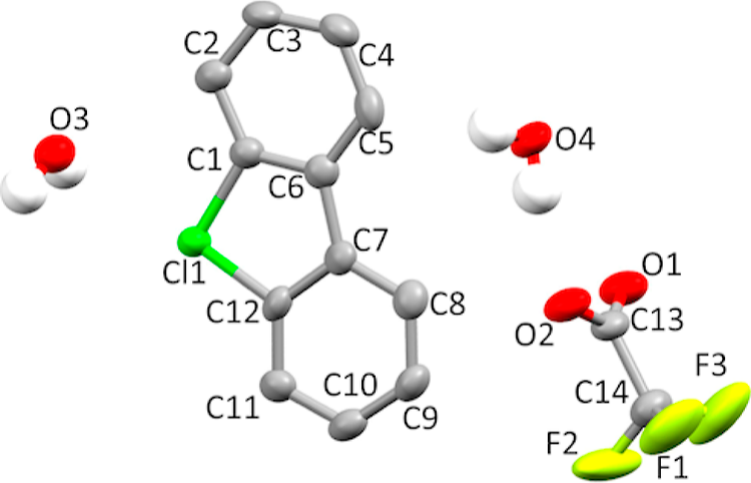
Thermal ellipsoid plot of **8a·2H**_**2**_**O** drawn to the 50% probability level. Nonoxygen hydrogen atoms were removed for clarity.

Single crystals of product **8a** (as a dihydrate) suitable for X-ray crystallographic analysis (Figure 5) were obtained by slow crystallization from MeOH–H_2_O solution. X-ray diffraction data for **8a·2H**_**2**_**O** were collected on a Rigaku RAPID II Image Plate system using graphite-monochromated Cu Kα radiation (λ = 1.54187 Å) at 173 K. The structure was solved by Sir2011 and refined using SHELXL-2014/7. Crystal data for **8a·2H**_**2**_**O** C_14_H_12_ClF_3_O_4_, orthorhombic, space group *P*2(1)2(1)2(1), *a* = 6.94200(10) Å, *b* = 9.1974(2) Å, *c* = 22.4103(16) Å, α = 90°, β = 90°, γ = 90°, *V* = 1430.86(11) Å^3^, *Z* = 4, 4994 reflections measured, 2418 unique reflections, 2038 *I* > 2/*s*(*I*), 212 parameters, 6 restraints; Flack parameter: 0.50(5); GooF = 1.132, final *R*_1_ = 0.0581, *R*_w_ (all) = 0.0734. CCDC 2278338.

The space group *P*2_1_2_1_2_1_ was assigned to structure 20201101-1 (CCDC 22783) using the app XPLAIN (XPLAIN 1.7.2 April 9, 2015, Copyright 2008–2015 Rigaku, Inc.) based on systematic absences consistent with three perpendicular screw axes. The data >5σ was especially convincing of the assignment of three perpendicular screw axes. Data modeled in space groups consistent with the screw axes and centrosymmetric structures did not yield sensible solutions.

##### Dibenzo[*b*,*d*]chlorol-5-ium Tetrafluoroborate **8b**(9)

Reaction with **6** (1130 mg, 5.6 mmol) and HBF_4_ according to the general procedure afforded 997 mg (65%) of product **8b**, isolated as a white solid: mp 189.4–192.1 °C; IR (KBr) cm^–1^: 3439, 3062, 1625, 1447, 1416, 1080, 755, 566; ^1^H NMR (400 MHz, CD_3_OD): δ 8.60 (dd, *J* = 8.0, 1.6 Hz, 2H), 8.52 (dd, *J* = 8.4, 1.0 Hz, 2H), 8.08–8.02 (m, 2H), 8.00–7.94 (m, 2H); ^13^C NMR (100 MHz, CD_3_OD): δ 141.2, 133.7, 133.5, 133.4, 126.8, 123.4; ^19^F NMR (377 MHz, CD_3_OD): δ −154.6; (ESI-positive ionization): calcd for C_12_H_8_Cl^35^ [M]^+^: 187.0309, found: 187.0324, and calcd for C_12_H_8_Cl^37^ [M]^+^: 189.0282, found: 189.0286.

**Figure 6:**
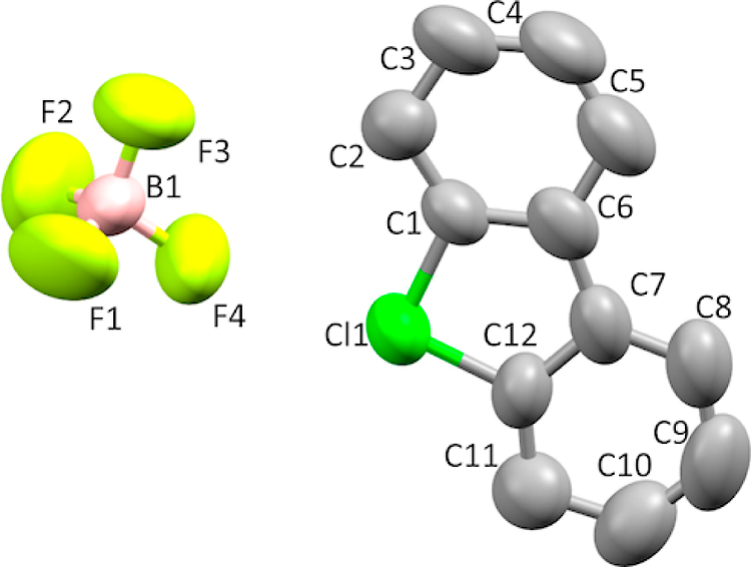
Thermal ellipsoid plot of **8b** drawn to the 50% probability level. Hydrogen atoms were removed for clarity. The X-ray structure for **8b** was previously published.^7^

Single crystals of product **8b** suitable for X-ray crystallographic analysis (Figure 6) were obtained by slow crystallization from MeOH–H_2_O solution. X-ray diffraction data for **8b** were collected on a Rigaku RAPID II Image Plate system using graphite-monochromated Cu Kα radiation (λ = 1.54187 Å) at 288 K. The structure was solved by Superflip and refined using SHELXL-2014/7. Crystal data for **8b** C_12_H_8_BClF_4_, monoclinic, space group *P*2(1)/*n*, *a* = 5.8786(2) Å, *b* = 15.9239(5) Å, *c* = 12.4693(8) Å, α = 90°, β = 90.853(6)°, γ = 90°, *V* = 1167.12(9) Å^3^, *Z* = 4, 9504 reflections measured, 2037 unique reflections, 900 *I* > 2/*s*(*I*), 163 parameters, 0 restraints; GooF = 1.118, final *R*_1_ = 0.0847, *R*_w_ (all) = 0.1392. CCDC 2278341.

#### General Procedure for Ligand Exchange Reactions

The appropriate inorganic salt was added to the solution of bromolium **2** or **7b** or chlorolium **8b** salt in MeOH or MeOH–H_2_O (1:1 or 5:1) and stirred at room temperature for 1 h. After completion of the reaction, the solvent was removed under reduced pressure to obtain a solid residue. Diethyl ether was added to the solid residue to form a suspension, which was filtered, washed several times with diethyl ether, and dried in vacuum to obtain the pure product **7f**–**i** or **8c**–**f**.

##### Dibenzo[*b*,*d*]bromol-5-ium Azide **7f**

Reaction with cyclic bromolium salt **7b** (191 mg, 0.5 mmol) and NaN_3_ (98 mg, 1.5 mmol) in 5:1 MeOH–H_2_O (9 mL) according to the general procedure for the ligand exchange afforded 55 mg (40%) of product **7f**, isolated as a white solid: mp 150 °C (explosion); IR (KBr) cm^–1^: 3660, 2013, 1587, 1409, 1121, 748, 623; ^1^H NMR (400 MHz, CD_3_OD): δ 8.52 (dd, *J* = 8.0, 1.6 Hz, 2H), 8.40 (d, *J* = 8.8 Hz, 2H), 8.02–7.95 (m, 2H), 7.89–7.84 (m, 2H); ^13^C NMR (75 MHz, CD_3_OD): δ 136.5, 135.9, 132.0, 131.6, 126.4, 125.1; HRMS (ESI-positive ionization): calcd for C_12_H_8_Br^79^ [M]^+^: 230.9804, found: 230.9815, and calcd for C_12_H_8_Br^81^ [M]^+^: 232.9784, found: 232.9799.

**Figure 7:**
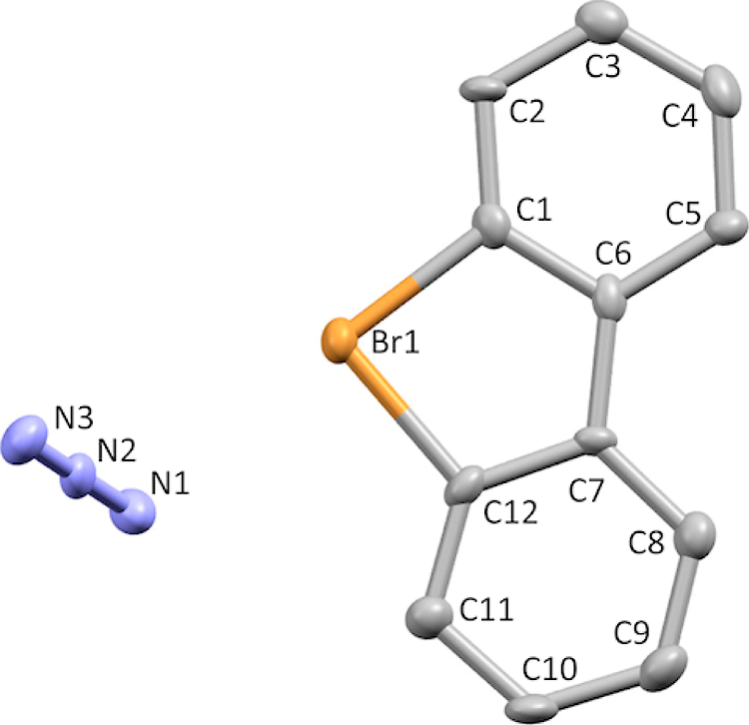
Thermal ellipsoid plot of **7f** drawn to the 50% probability level. Hydrogen atoms were removed for clarity.

Single crystals of product **7f** suitable for X-ray crystallographic analysis (Figure 7) were obtained by slow crystallization from MeOH–H_2_O solution. X-ray diffraction data for **7f** were collected on a Rigaku RAPID II Image Plate system using graphite-monochromated Cu Kα radiation (λ = 1.54187 Å) at 123 K. The structure was solved by Sir 2011 and refined using SHELXL-2014/7. Crystal data for **7f** C_12_H_8_BrN_3_, triclinic, space group *P*1̅, *a* = 7.29030(10) Å, *b* = 8.7296(2) Å, *c* = 9.5278(7) Å, α = 112.453(8)°, β = 92.228(7)°, γ = 110.533(8)°, *V* = 514.22(5) Å^3^, *Z* = 2, 5403 reflections measured, 1708 unique reflections, 1403 *I* > 2/*s*(*I*), 145 parameters, 42 restraints; GooF = 1.068, final *R*_1_ = 0.0338, *R*_w_ (all) = 0.0442. CCDC 2278334.

##### Dibenzo[*b*,*d*]bromol-5-ium Bis(trifluoromethanesulfonyl)imidate **7g**

Reaction with bromolium salt **7b** (64 mg, 0.2 mmol) and LiNTf_2_ (172 mg, 0.6 mmol) in MeOH–H_2_O (2 mL) according to the general procedure afforded 102 mg (100%) of product **7g**, isolated as a white solid: mp 118–120 °C; IR (KBr) cm^–1^: 3443, 1643, 1357, 1195, 1139, 1055, 748, 588; ^1^H NMR (400 MHz, CD_3_CN): δ 8.43 (dd, *J* = 7.7, 1.8 Hz, 2H), 8.28 (d, *J* = 8.8 Hz, 2H), 7.98 (t, *J* = 7.7 Hz, 2H), 7.88–7.83 (m, 2H); ^13^C NMR (100 MHz, CD_3_CN): δ 137.3, 136.1, 133.0, 132.4, 127.3, 125.6, 120.5 (^1^*J*_CF_ = 318.7 Hz); ^19^F NMR (376 MHz, CD_3_CN): δ −80.7; HRMS (ESI-positive ionization): calcd for C_12_H_8_Br^79^ [M]^+^: 230.9804, found: 230.9814, and calcd for C_12_H_8_Br^81^ [M]^+^: 232.9784, found: 232.9799.

Reaction with cyclic bromolium salt **2** (200 mg, 0.747 mmol) and AgNTf_2_ (334 mg, 0.860 mmol) in MeOH (14 mL) according to the general procedure afforded analytically pure product **7g**; 353 mg (92%) isolated as a light-yellow solid identical to the sample from the previous experiment.

**Figure 8:**
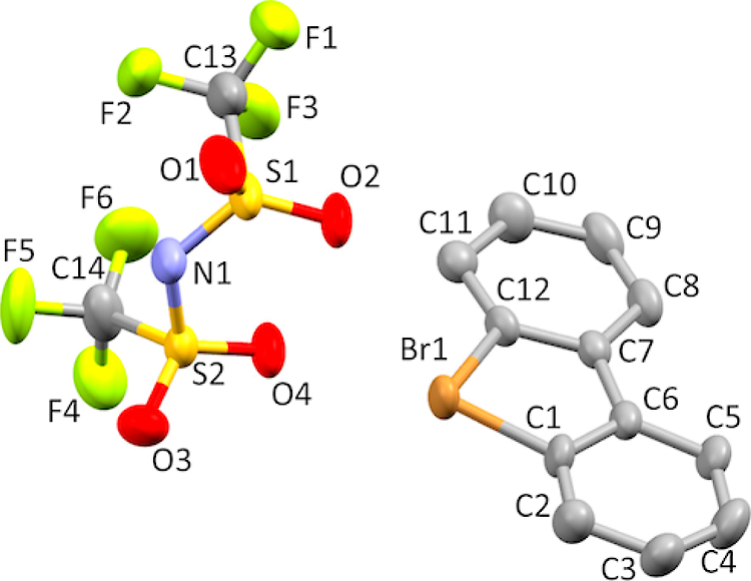
Thermal ellipsoid plot of **7g** drawn to the 50% probability level. Hydrogen atoms were removed for clarity. Only one molecule and counterion of interest out of three are shown for clarity.

Single crystals of product **7g** suitable for X-ray crystallographic analysis (Figure 8) were obtained by slow crystallization from MeOH–H_2_O solution. X-ray diffraction data for **7g** were collected on a Rigaku RAPID II Image Plate system using graphite-monochromated Cu Kα radiation (λ = 1.54187 Å) at 123 K. The structure was solved by Sir 2011 and refined using SHELXL-2014/7.^13^ Crystal data for **7g** C_14_H_8_Br_1_F_6_N_1_O_4_S_2_, triclinic, space group *P*1̅, *a* = 11.3077(2) Å, *b* = 11.9852(2) Å, *c* = 17.4687(12) Å, α = 108.334(8)°, β = 101.247(7)°, γ = 91.519(6)°, *V* = 2194.28(19) Å^3^, *Z* = 5, 24,326 reflections measured, 7480 unique reflections, 6054 *I* > 2/*s*(*I*), 787 parameters, 1348 restraints; GooF = 1.104, final *R*_1_ = 0.0996, *R*_w_ (all) = 0.1156. CCDC 2278335.

Significant disorder was modeled in structure **7g**. Three independent molecules of interest and three independent counteranions were found in the unit cell with one pair on an inversion center. Two of the three anions and molecules of interest were modeled with significant disorder. One counteranion with the “B” series label was modeled over an inversion center with 50% occupancy with the Part −1 command and the 1–2 and 1–3 lengths between atoms were restrained with the SAME command to be the same as the counterion that was modeled over one position. Furthermore, the atoms neighboring by the inversion center were constrained to have the same anisotropic displacement parameters with the EADP command and nonpositive definite atoms were restrained to be more isotropic with the ISOR command.

The second counteranion (labeled “A” and “C” series) was modeled over two positions and were restrained to have the same 1–2 and 1–3 atom distances and constrained to have the same anisotropic displacement parameters using the SAME and EADP command as mentioned previously and the atom. The occupancy of the two positions refined to an occupancy of 61–39%.

One of the disordered molecules of interest (labeled “A” and “C” series) was modeled over two positions and the occupancy refined to a 59–41%. The anisotropic displacement parameters of neighboring atoms were constrained with the EADP command. The 1–2 and 1–3 atom distances were restrained to be the same as the molecule of interest that was modeled in a single position using the SAME command. Both clockwise and anticlockwise directions of the 1–2 and 1–3 distances were used due to molecular symmetry.

The second disordered molecule of interest (labeled “B” and “D” series) was modeled over two positions and an inversion center using the PART −1 command. Interestingly, the four positions refine to a 0.24:0.24:0.26:0.26 occupancy ratio. The 1–2 and 1–3 atom distances were restrained to be the same as the molecule of interest that was modeled as a single position using the SAME command. Both clockwise and anticlockwise directions of the 1–2 and 1–3 distances were used due to molecular symmetry. Finally, the atoms neighboring by the inversion center and disorder were constrained to have the same anisotropic displacement parameters with the EADP command and nonpositive definite atoms were restrained to be more isotropic with the ISOR command.

##### Dibenzo[*b*,*d*]bromol-5-ium Thiocyanate **7h**(14)

Reaction with bromolium salt **7b** (64 mg, 0.2 mmol) and KSCN (172 mg, 0.6 mmol) in MeOH–H_2_O (2 mL) according to the general procedure afforded 37.0 mg (63%) of product **7h**, isolated as a white solid: mp; 171.5–174.2 °C; IR (KBr) cm^–1^: 3433, 3051, 2041, 1408, 1116, 751; ^1^H NMR (400 MHz, CD_3_CN): δ 8.42 (dd, *J* = 8.0, 1.6 Hz, 2H), 8.35 (d, *J* = 8.4 Hz, 2H), 8.00–7.94 (m, 2H), 7.92–7.82 (m, 2H); ^13^C NMR (100 MHz, CD_3_CN): δ 137.5, 136.1, 132.9, 132.3, 129.8, 127.2, 125.7; HRMS (ESI-positive ionization): calcd for C_12_H_8_Br^79^ [M]^+^: 230.9804, found: 230.9819, and calcd for C_12_H_8_Br^81^ [M]^+^: 232.9784, found: 232.9805.

##### Dibenzo[*b*,*d*]bromol-5-ium Trifluoromethanesulfonate **7i**(12)

Reaction with cyclic bromolium salt **7b** (64 mg, 0.2 mmol) and LiOTf (94 mg, 0.6 mmol) in MeOH–H_2_O (2 mL) according to the general procedure afforded 48.2 mg (63%) of product **7i**, isolated as a white solid: mp 202.3–204.8 °C; IR (KBr) cm^–1^: 3447, 3099, 1624, 1451, 1263, 1165, 1029, 752, 634; ^1^H NMR (400 MHz, CD_3_CN): δ 8.42 (dd, *J* = 8.0, 1.6 Hz, 2H), 8.30 (dd, *J* = 8.8, 0.8 Hz, 2H), 8.01–7.94 (m, 2H), 7.89–7.82 (m, 2H); ^13^C NMR (100 MHz, CD_3_CN): δ 137.1, 135.9, 132.7, 132.1, 127.1, 125.5, 119.9 (^1^*J*_CF_ = 318.5 Hz); ^19^F NMR (376 MHz, CD_3_CN): δ −79.3; HRMS (ESI-positive ionization): calcd for C_12_H_8_Br^79^ [M]^+^: 230.9804, found: 230.9814, and calcd for C_12_H_8_Br^81^ [M]^+^: 232.9784, found: 232.9802.

Reaction with bromolium salt **2** (200 mg, 0.747 mmol) and AgOTf (221 mg, 0.860 mmol) in MeOH–H_2_O (7.5 mL) according to the general procedure afforded analytically pure product **7i**; 284 mg (99%) isolated as a white solid identical to the sample from the previous experiment.

**Figure 9:**
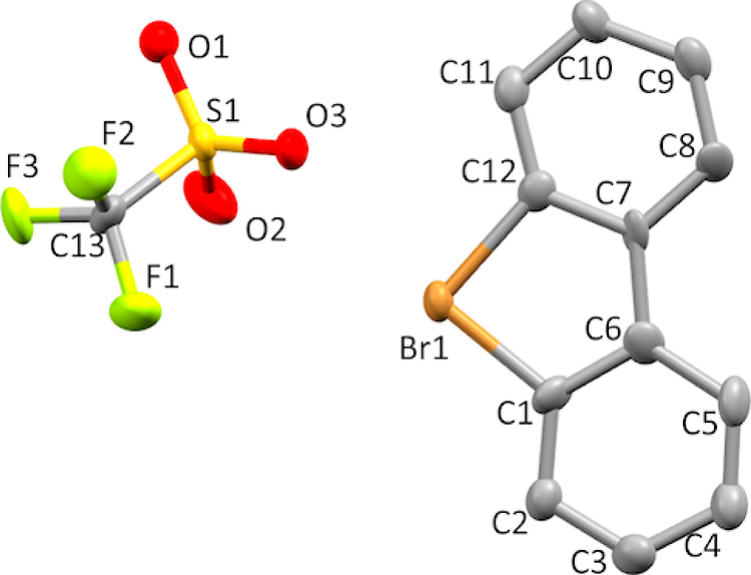
Thermal ellipsoid plot of **7i** drawn to the 50% probability level. Hydrogen atoms were removed for clarity.

Single crystals of product **7i** suitable for X-ray crystallographic analysis (Figure 9) were obtained by slow crystallization from MeOH–H_2_O solution. X-ray diffraction data for **7i** were collected on a Rigaku RAPID II Image Plate system using graphite-monochromated Mo Kα radiation (λ = 0.71073 Å) at 173 K. The structure was solved by Superflip and refined using SHELXL-2014/7. Crystal data for **7i** C_13_H_8_BrF_3_O_3_S, monoclinic, space group *P*2(1)/*c*, *a* = 7.2055(4) Å, *b* = 16.2633(11) Å, *c* = 11.2972(9) Å, α = 90°, β = 91.134(6)°, γ = 90°, *V* = 1323.61(16) Å^3^, *Z* = 4, 4621 reflections measured, 2266 unique reflections, 1511 *I* > 2/*s*(*I*), 190 parameters, 72 restraints; GooF = 1.108, final *R*_1_ = 0.0645, *R*_w_ (all) = 0.1121. CCDC 2278332.

##### Dibenzo[*b*,*d*]chlorol-5-ium Azide **8c**

Reaction with chlorolium salt **8b** (55 mg, 0.2 mmol) and NaN_3_ (39 mg, 0.6 mmol) in 1:1 MeOH–H_2_O (2 mL) according to the general procedure afforded 36.4 mg (79%) of product **8c**, isolated as a white solid: mp 173.5–174.9 °C; IR (KBr) cm^–1^: 3440, 3063, 2118, 1624, 1448, 1082, 757; ^1^H NMR (400 MHz, CD_3_OD): δ 8.60 (dd, *J* = 7.6, 1.6 Hz, 2H), 8.52 (d, *J* = 8.0 Hz, 2H), 8.08–8.02 (m, 2H), 8.01–7.95 (m, 2H); ^13^C NMR (100 MHz, CD_3_OD): δ 139.8, 132.3, 132.1, 131.9, 125.4, 122.0; (ESI-positive ionization): calcd for C_12_H_8_Cl^35^ [M]^+^: 187.0309, found: 187.0318, and calcd for C_12_H_8_Cl^37^ [M]^+^: 189.0282, found: 189.0286.

##### Dibenzo[*b*,*d*]chlorol-5-ium Bis(trifluoromethanesulfonyl)imidate **8d**

Reaction with chlorolium salt **8b** (55 mg, 0.2 mmol) and LiNTf_2_ (172 mg, 0.6 mmol) in 1:1 MeOH–H_2_O (2 mL) according to the general procedure afforded 57.1 mg (61%) of product **8d**, isolated as a white solid: mp 90.6–92.5 °C; IR (KBr) cm^–1^: 3437, 1634, 1344, 1190, 1136, 1056, 752, 655; ^1^H NMR (400 MHz, CD_3_CN): δ 8.49 (dd, *J* = 8.0 Hz, 1.6 Hz, 2H), 8.38 (d, *J* = 8.8 Hz, 2H), 8.05–8.00 (m, 2H), 7.98–7.91 (m, 2H); ^13^C NMR (75 MHz, CD_3_OD): δ 140.0, 132.5, 132.3, 132.2, 125.7, 122.2, 120.0 (^1^*J*_CF_ = 318.7 Hz); ^19^F NMR (376 MHz, CD_3_CN): δ −80.2; (ESI-positive ionization): calcd for C_12_H_8_Cl^35^ [M]^+^: 187.0309, found: 187.0326, and calcd for C_12_H_8_Cl^37^ [M]^+^: 189.0282, found: 189.0289.

**Figure 10:**
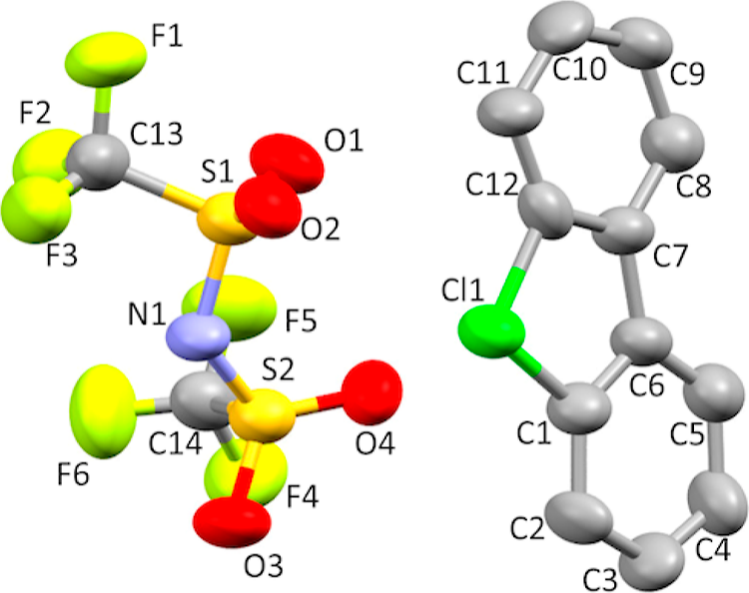
Thermal ellipsoid plot of **8d** drawn to the 50% probability level. Hydrogen atoms were removed for clarity.

Single crystals of product **8d** suitable for X-ray crystallographic analysis (Figure 10) were obtained by slow crystallization from MeOH–H_2_O solution. X-ray diffraction data for **8d** were collected on a Rigaku RAPID II Image Plate system using graphite-monochromated Cu Kα radiation (λ = 1.54187 Å) at 173 K. The structure was solved by Sir 2011 and refined using SHELXL-2014/7. Crystal data for **8d** C_14_H_8_ClF_6_NO_4_S_2_, triclinic, space group *P*1̅, *a* = 8.2454(3) Å, *b* = 10.9498(4) Å, *c* = 11.2133(8) Å, α = 115.897(8)°, β = 100.605(7)°, γ = 95.653(7)°, *V* = 876.67(10) Å^3^, *Z* = 2, 7230 reflections measured, 2963 unique reflections, 1285 *I* > 2/*s*(*I*), 253 parameters, 0 restraints; GooF = 1.001, final *R*_1_ = 0.1077, *R*_w_ (all) = 0.1791. CCDC 2278339.

##### Dibenzo[*b*,*d*]chlorol-5-ium Trifluoromethanesulfonate **8e**

Reaction with chlorolium salt **8b** (110 mg, 0.4 mmol) and LiOTf (186 mg, 1.2 mmol) in 1:1 MeOH–H_2_O (4 mL) according to the general procedure afforded 118.7 mg (88%) of product **8e**, isolated as a white solid: mp 186.0–192.4 °C; IR (KBr) cm^–1^: 3439, 3091, 1629, 1453, 1292, 1159, 1043, 754, 645; ^1^H NMR (400 MHz, CD_3_CN): δ 8.49 (dd, *J* = 8.0, 1.6 Hz, 2H), 8.39 (d, *J* = 8.8 Hz, 2H), 8.06–8.00 (m, 2H), 7.97–7.91 (m, 2H); ^13^C NMR (100 MHz, CD_3_CN): δ 140.3, 133.1, 132.8, 126.4, 122.8, 121.7 (^1^*J*_CF_ = 318.5 Hz); ^19^F NMR (376 MHz, CD_3_CN): δ −79.4; (ESI-positive ionization): calcd for C_12_H_8_Cl^35^ [M]^+^: 187.0309, found: 187.0318, and calcd for C_12_H_8_Cl^37^ [M]^+^: 189.0282, found: 189.0280.

**Figure 11:**
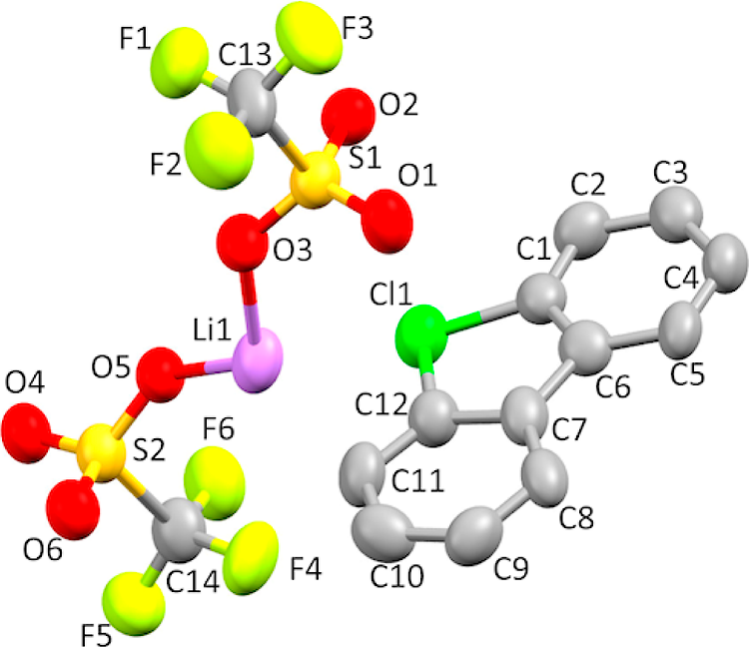
Thermal ellipsoid plot of **8e·LiOTf** drawn to the 50% probability level. Nonoxygen hydrogen atoms were removed for clarity.

Single crystals of product **8e·LiOTf** suitable for X-ray crystallographic analysis (Figure 11) were obtained by slow crystallization from MeOH–H_2_O solution. X-ray diffraction data for **8e·LiOTf** were collected on a Rigaku RAPID II Image Plate system using graphite-monochromated Cu Kα radiation (λ = 1.54187 Å) at 173 K. The structure was solved by Sir 2011 and refined using SHELXL-2014/7. Crystal data for **8e·LiOTf** C_14_H_8_ClF_6_LiNO_6_S_2_, triclinic, space group *P*1̅, *a* = 9.0350(6) Å, *b* = 10.5743(7) Å, *c* = 10.9780(8) Å, α = 62.017(4)°, β = 81.475(6)°, γ = 85.939(6)°, *V* = 915.97(11) Å^3^, *Z* = 2, 7619 reflections measured, 3121 unique reflections, 1546 *I* > 2/*s*(*I*), 271 parameters, 0 restraints; GooF = 1.022, final *R*_1_ = 0.1119, *R*_w_ (all) = 0.1914. CCDC 2278340.

##### Dibenzo[*b*,*d*]chlorol-5-ium Thiocyanate **8f**

Reaction with chlorolium salt **8b** (55 mg, 0.2 mmol) and KSCN (58 mg, 0.6 mmol) in 1:1 MeOH–H_2_O (2 mL) according to the general procedure afforded 49 mg (100%) of product **8f**, isolated as a white solid: mp 106.5–110.3 °C; IR (KBr) cm^–1^: 3456, 2059, 1626, 1467, 747; ^1^H NMR (400 MHz, CD_3_OD): δ 8.60 (dd, *J* = 7.6, 1.6 Hz, 2H), 8.53 (d, *J* = 8.4 Hz, 2H), 8.08–8.02 (m, 2H), 8.01–7.94 (m, 2H); ^13^C NMR (100 MHz, CD_3_OD): δ 139.9, 132.3, 132.1, 132.0, 131.1, 125.4, 122.0; (ESI-positive ionization): calcd for C_12_H_8_Cl^35^ [M]^+^: 187.0309, found: 187.0321, and calcd for C_12_H_8_Cl^37^ [M]^+^: 189.0282, found: 189.0290.

**Figure 12:**
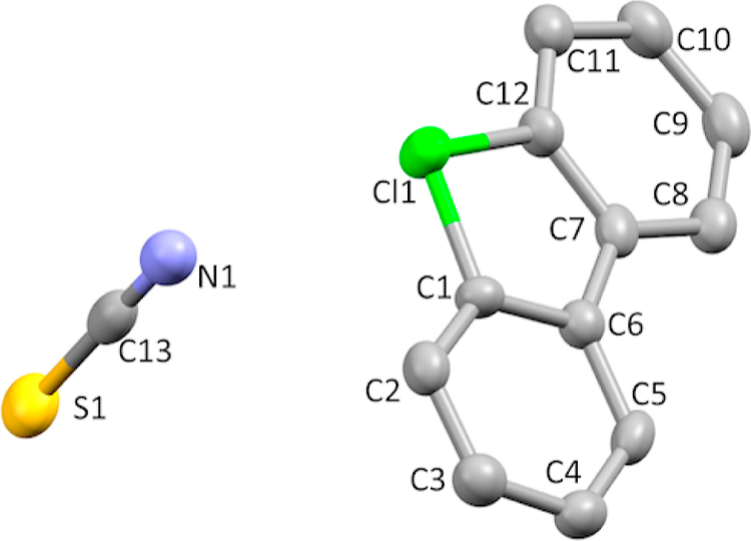
Thermal ellipsoid plot of **8f** drawn to the 50% probability level. Nonoxygen hydrogen atoms were removed for clarity.

Single crystals of product **8f** suitable for X-ray crystallographic analysis (Figure 12) were obtained by slow crystallization from MeOH–H_2_O solution. X-ray diffraction data for **8f** were collected on a Rigaku RAPID II Image Plate system using graphite-monochromated Cu Kα radiation (λ = 1.54187 Å) at 173 K. The structure was solved by DIRDIF v2008.3[1] and refined using SHELXL-2014/7. Crystal data for **8f** C_13_H_8_ClNS, monoclinic, space group *P*2(1)/*c*, *a* = 6.7605(15) Å, *b* = 18.1687(3) Å, *c* = 9.4972(7) Å, α = 90°, β = 109.973(9)°, γ = 90°, *V* = 1096.4(3) Å^3^, *Z* = 4, 5770 reflections measured, 1906 unique reflections, 1382 *I* > 2/*s*(*I*), 145 parameters, 0 restraints; GooF = 1.017, final *R*_1_ = 0.0463, *R*_w_ (all) = 0.0636. CCDC 2278337.

## Data Availability

The data underlying this study are available in the published article and its Supporting Information.

## Supporting Information Available

X-ray data for compounds **7** and **8** (CIF), and copies of NMR spectra for all compounds (PDF)
