# Supplementary material for: Preparation and X-ray Structural Study of Dibenzobromolium and Dibenzochlorolium Derivatives
Source: ACS Omega. 2024 Jan 3;9(2):2664–73. doi: 10.1021/acsomega.3c07512 (PMC10795028; doi:10.1021/acsomega.3c07512)

Supporting Information

Preparation and X-ray Structural Study of Dibenzobromolium and Dibenzochlorolium Derivatives

Christopher D. Huss,[a] Akira Yoshimura,*[a,b] Gregory T. Rohde,*[c] Irina A. Mironova,[d] Pavel S. Postnikov,[d,e] Mekhman S. Yusubov,[d] Akio Saito,[f] and Viktor V. Zhdankin*[a]

[a]*Department of Chemistry and Biochemistry, University of Minnesota Duluth, Duluth, Minnesota, 55812. E-mail: vzhdanki@d.umn.edu*

[b]*Faculty of Pharmaceutical Sciences, Aomori University, 2-3-1 Kobata, Aomori 030-0943, Japan. E-mail: ayoshimura@aomori-u.ac.jp*

[c]*Marshall School, Duluth, Minnesota 55811, USA. E-mail: greg.rohde@marshallschool.org*

[d]*Research School of Chemistry and Applied Biomedical Sciences, The Tomsk Polytechnic University, 634050 Tomsk, Russia*

[e]*Department of Solid-State Engineering, University of Chemistry and Technology, Prague 16628, Czech Republic*

[f]*Division of Applied Chemistry, Institute of Engineering, Tokyo University of Agriculture and Technology, 2-23-16 Naka-cho, Koganei, Tokyo 184-8588*

Contents:

NMR Spectra of products

Figure S1. Compound 2: ^{1}H NMR (400 MHz, DMSO-d_6)

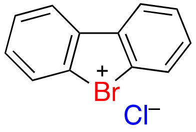

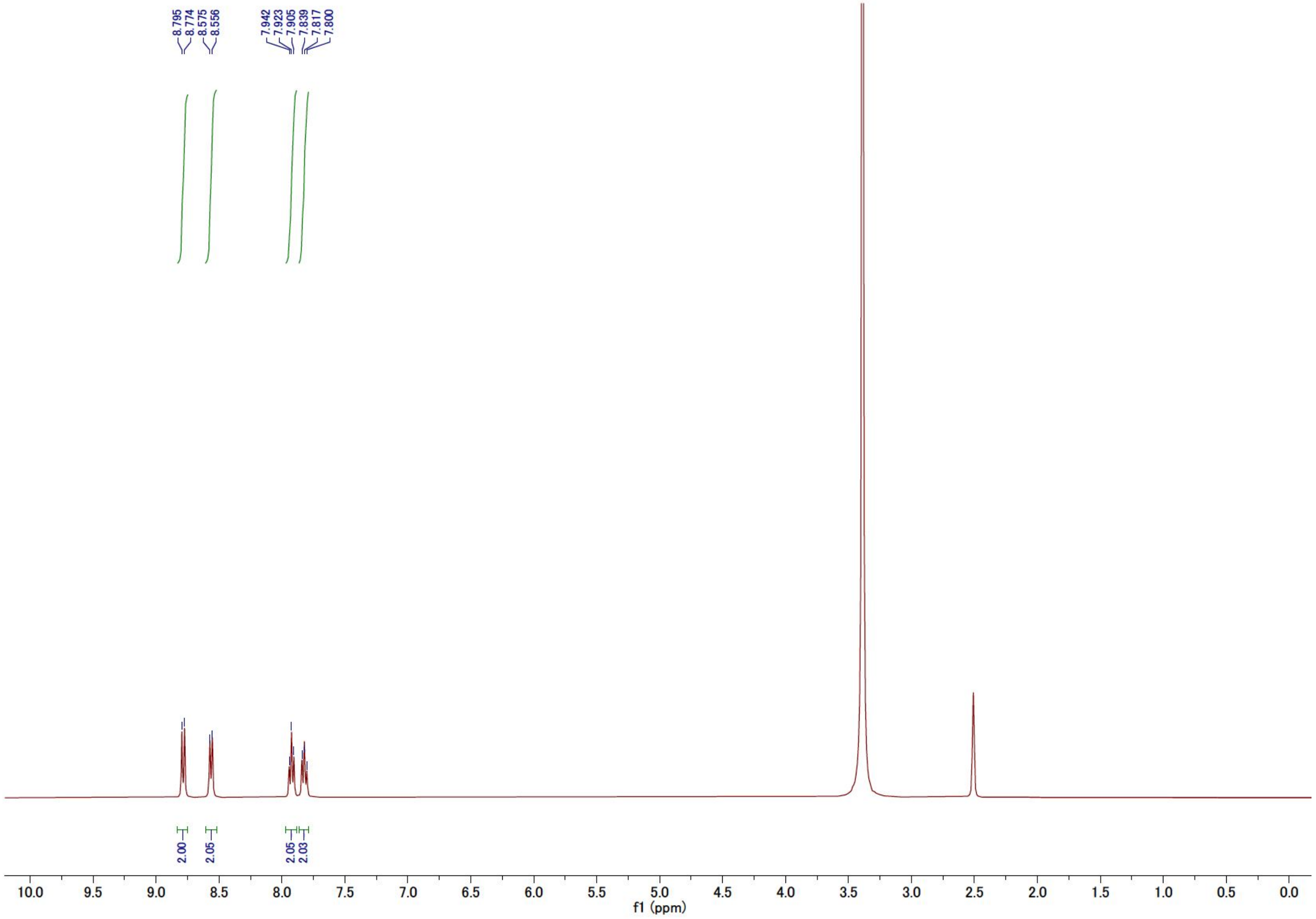

Figure S2. Compound 2: ^{13}C NMR (100 MHz, DMSO-d_6)

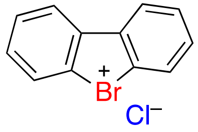

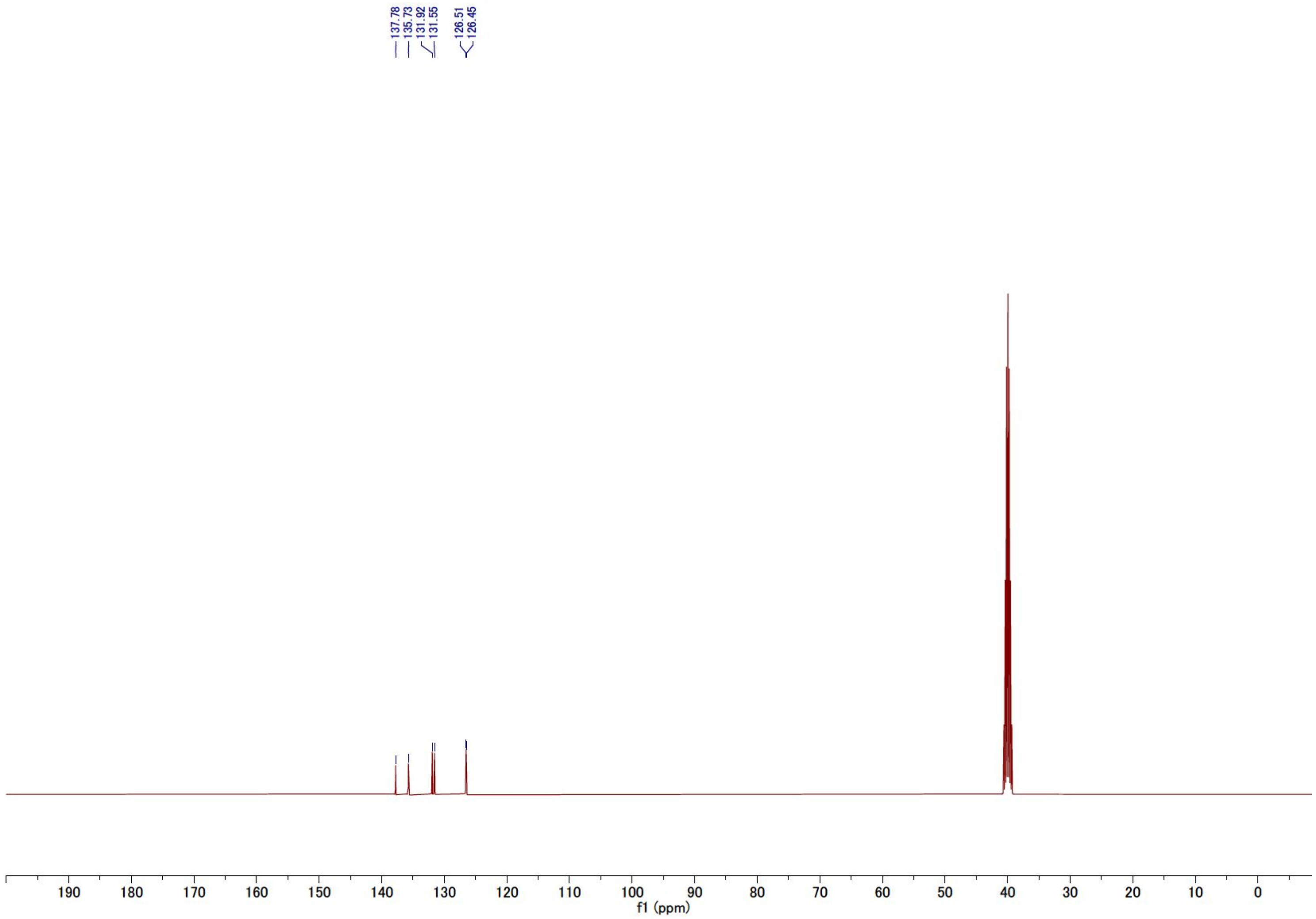

Figure S3. Compound 7a: ^1H NMR (500 MHz, CD_3OD)

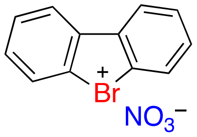

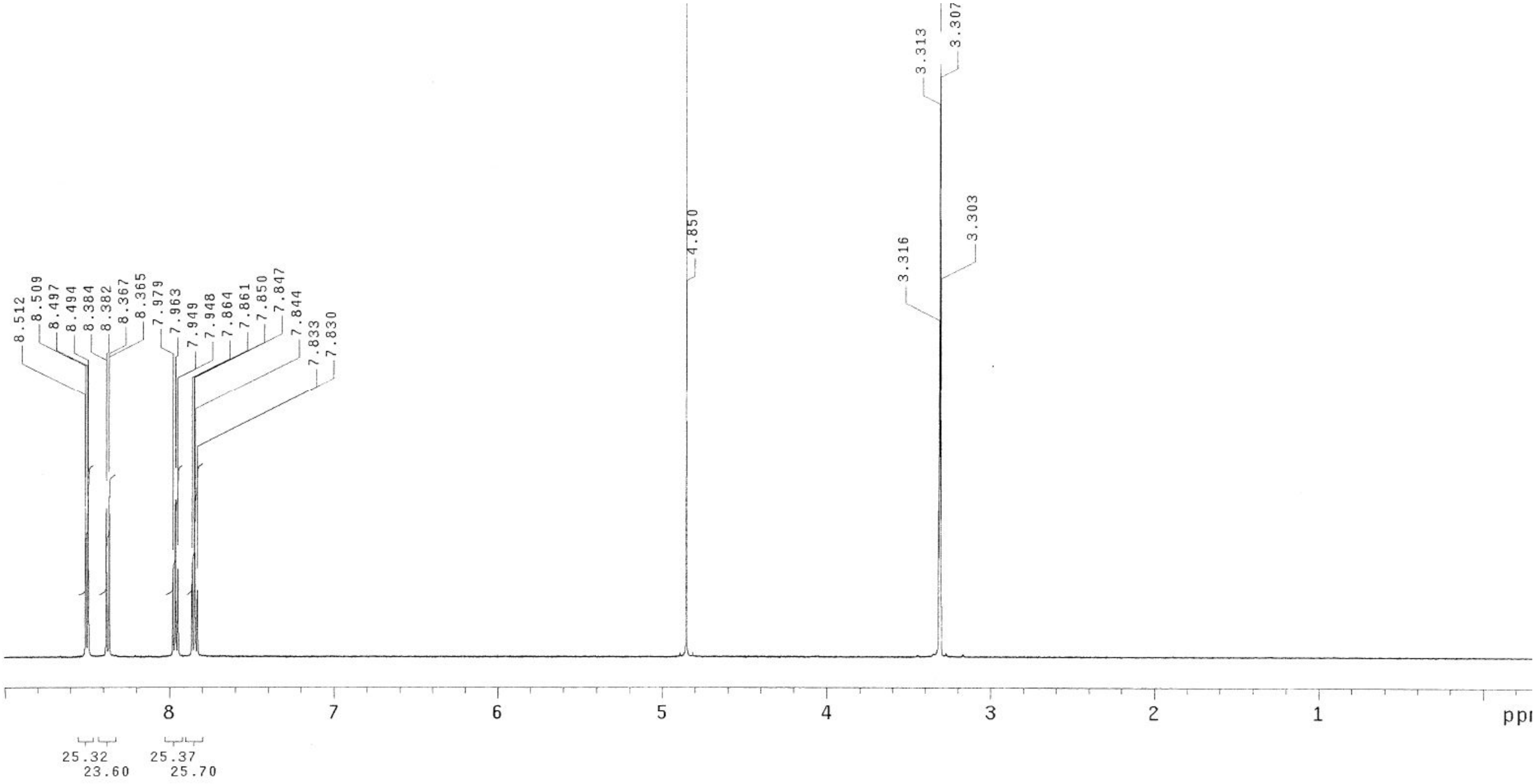

Figure S4. Compound 7a: ^{13}C NMR (75 MHz, CD_3OD)

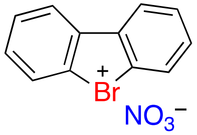

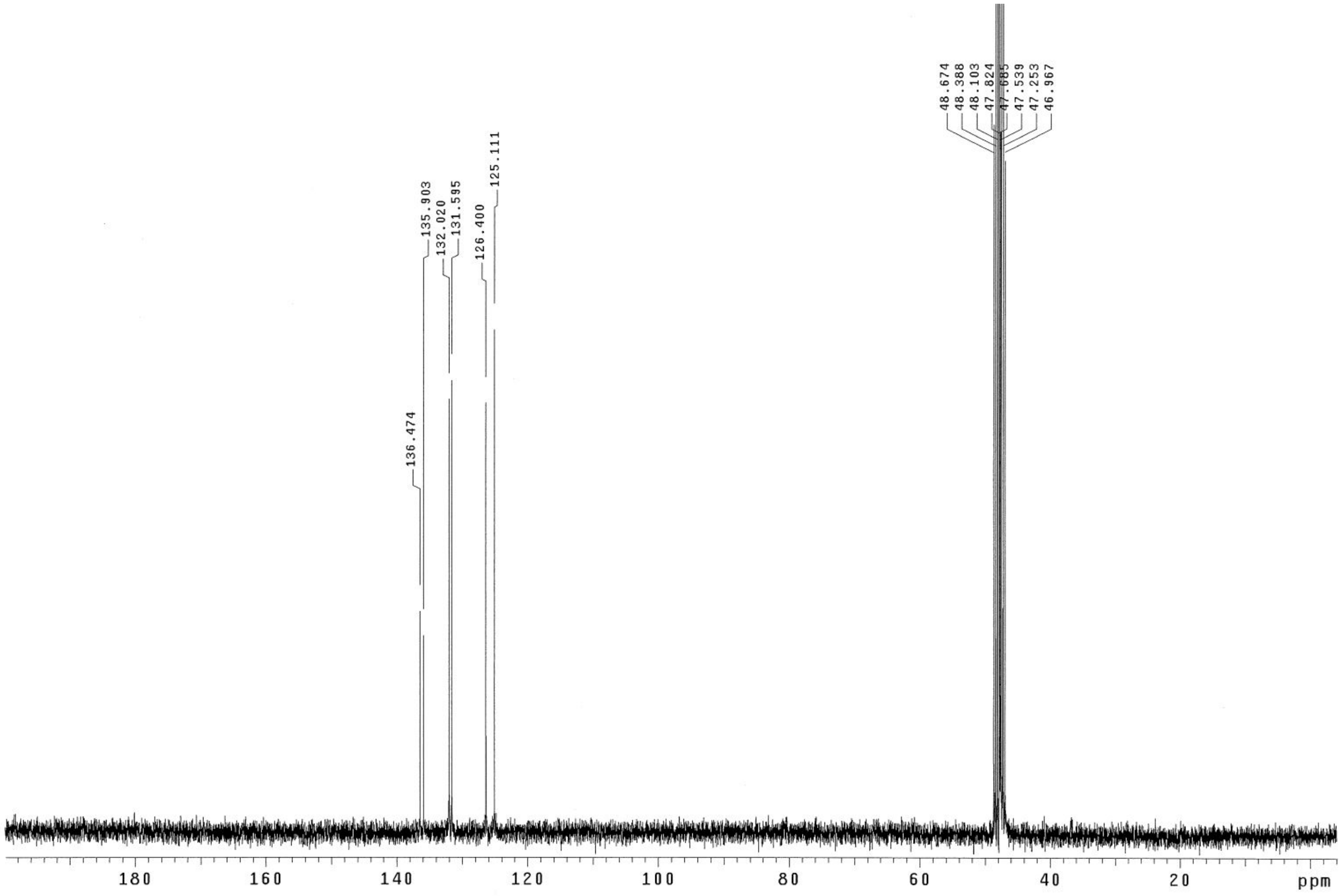

Figure S5. Compound 7b: ^{1}H NMR (400 MHz, CD_3CN)

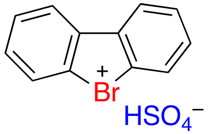

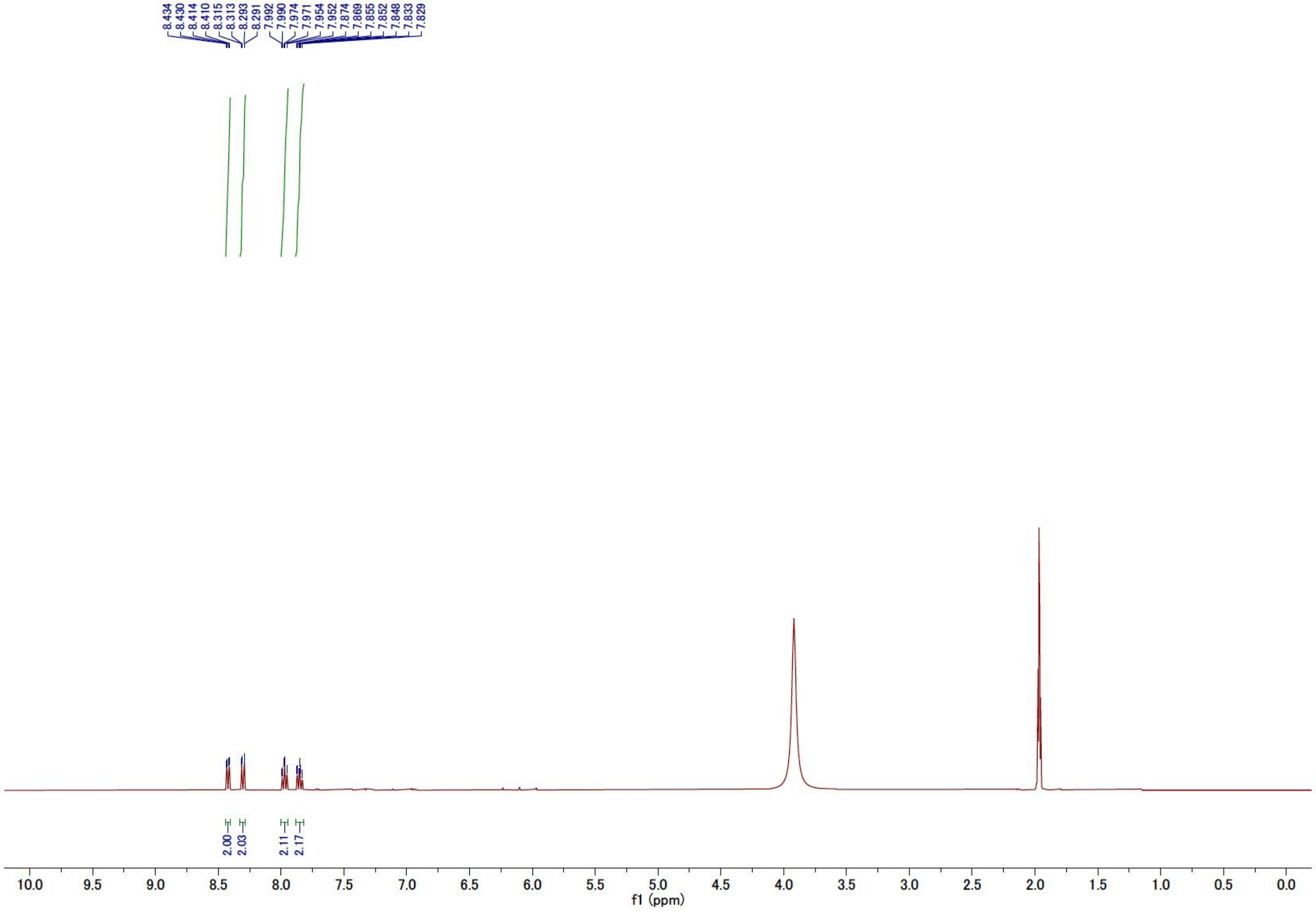

Figure S6. Compound 7b: ^{13}C NMR (100 MHz, CD_3CN)

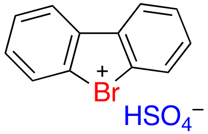

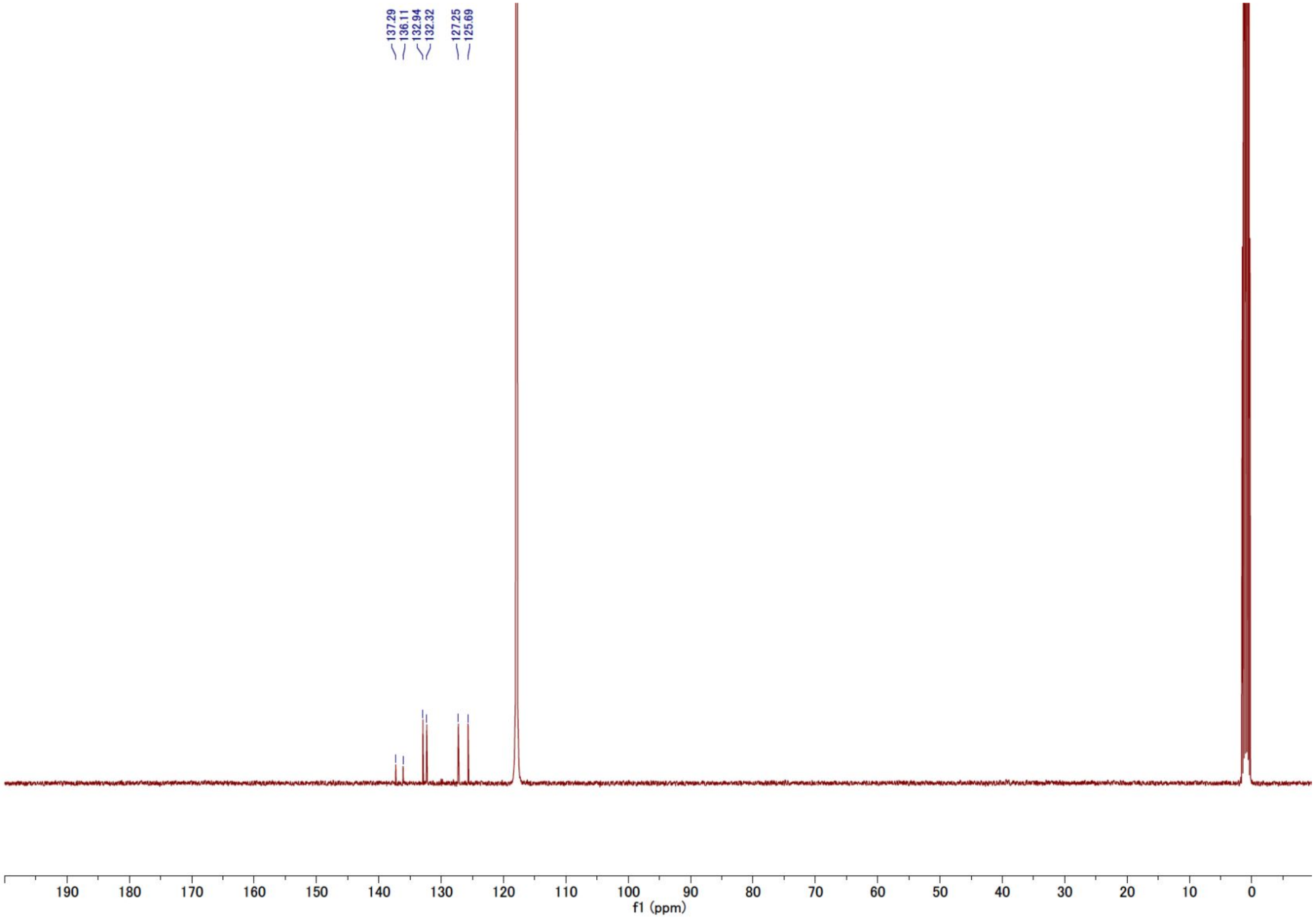

Figure S7. Compound 7c: ^{1}H NMR (400 MHz, CD_3CN)

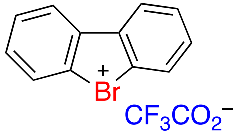

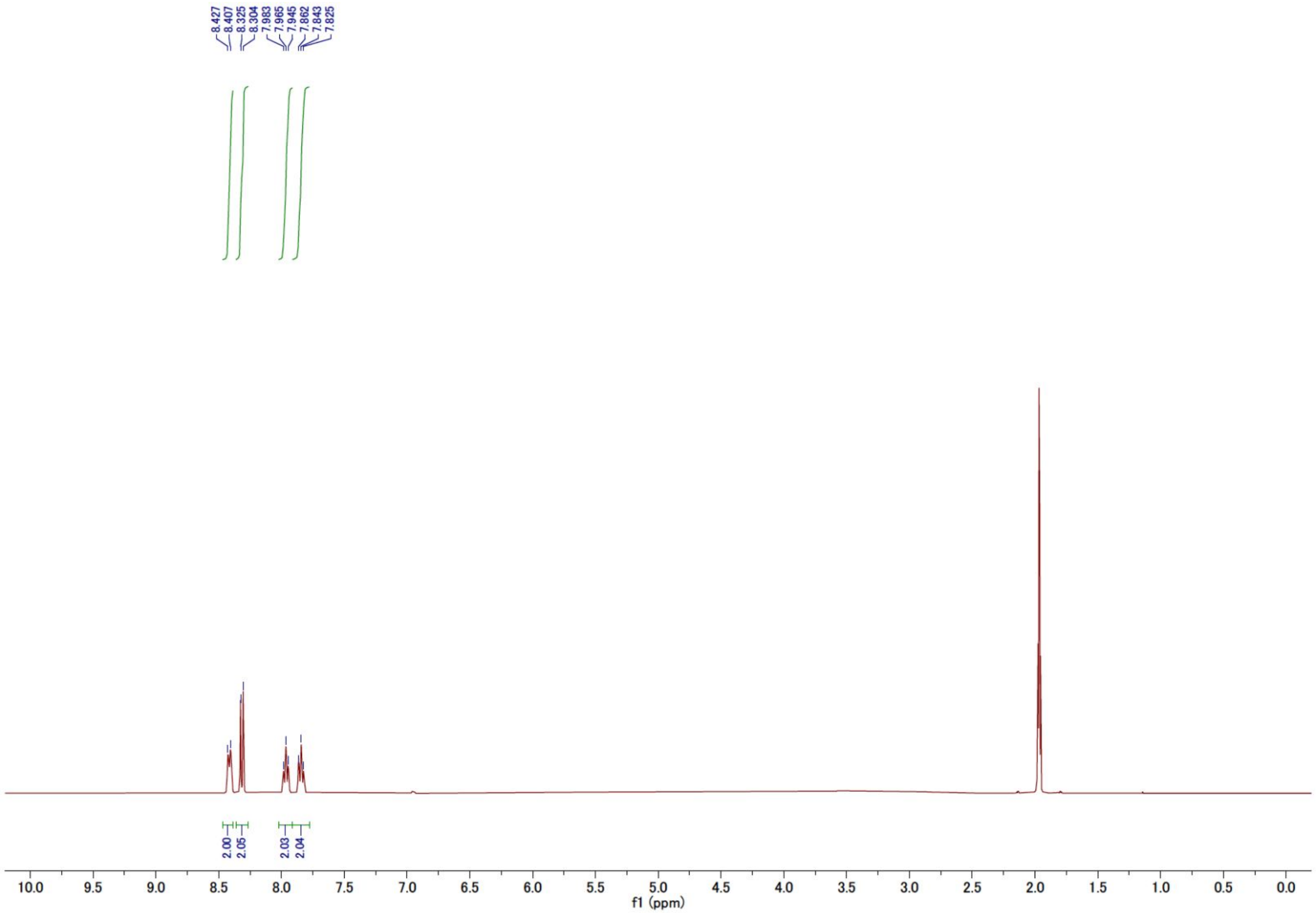

Figure S8. Compound 7c: ^{13}C NMR (100 MHz, CD_3CN)

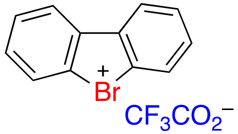

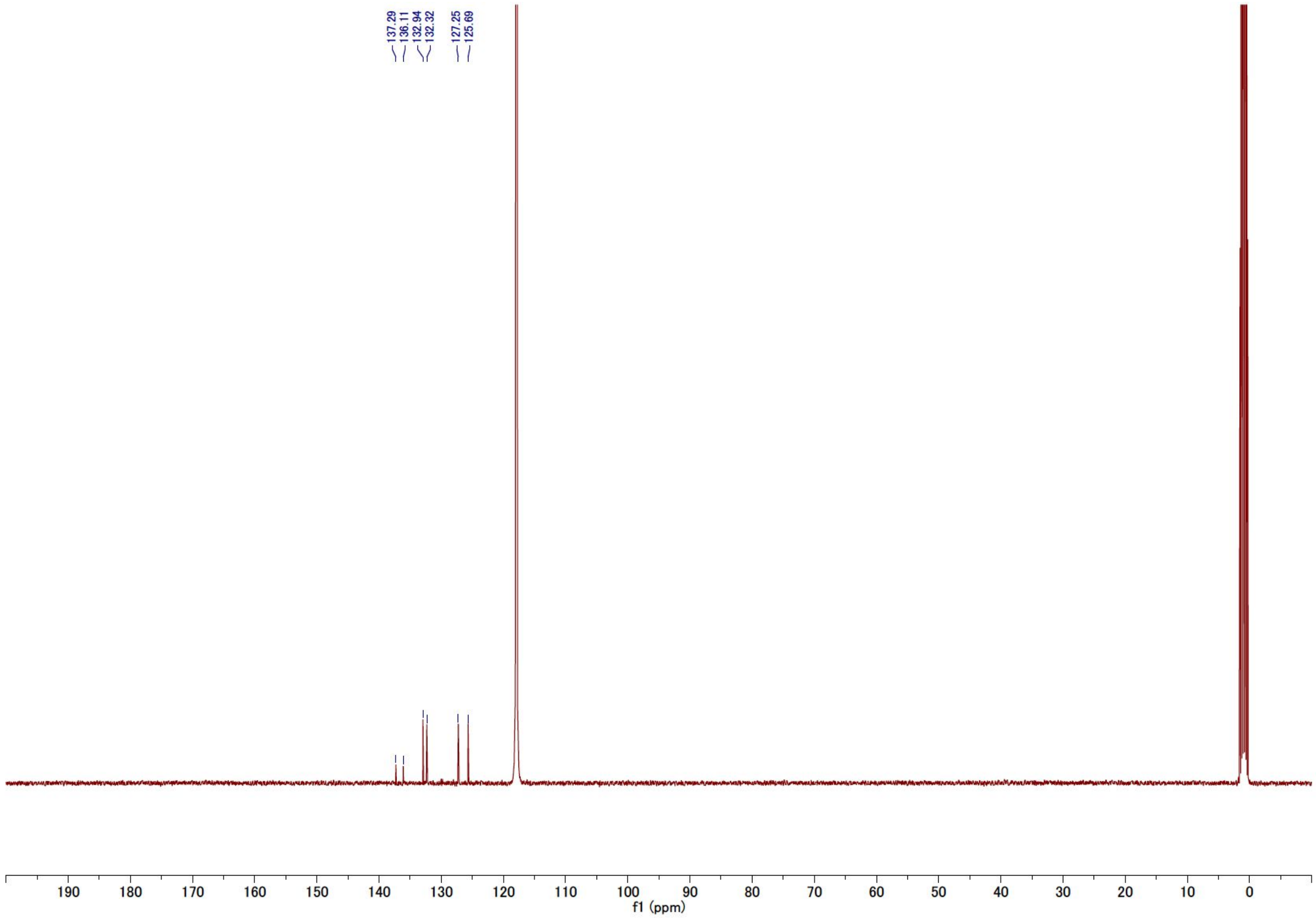

Figure S9. Compound 7c: ^{19}F NMR (376 MHz, CD_3CN)

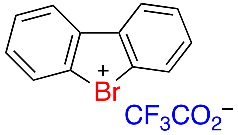

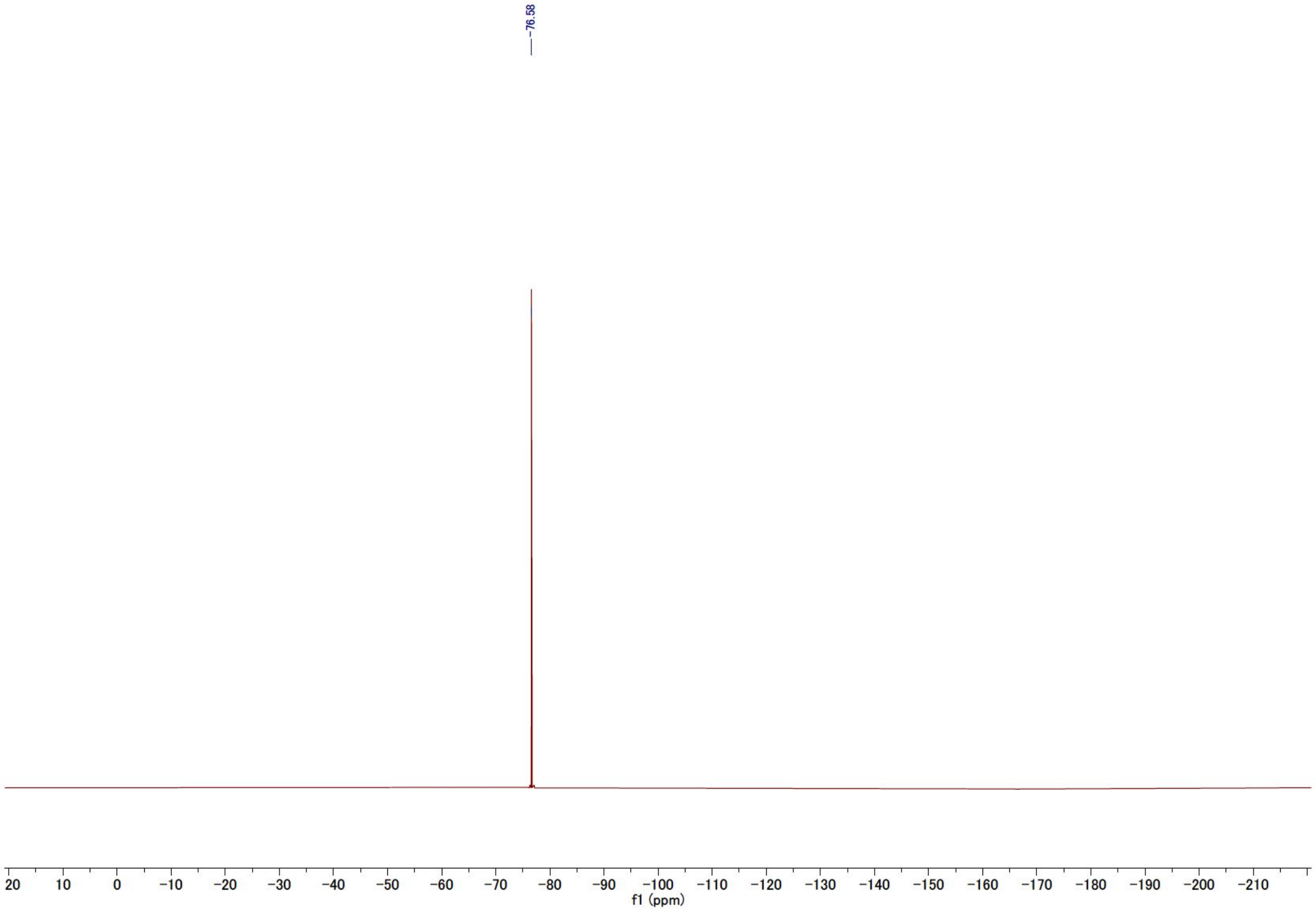

Figure S10. Compound 7d: ^{1}H NMR (500 MHz, DMSO-d_6)

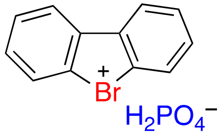

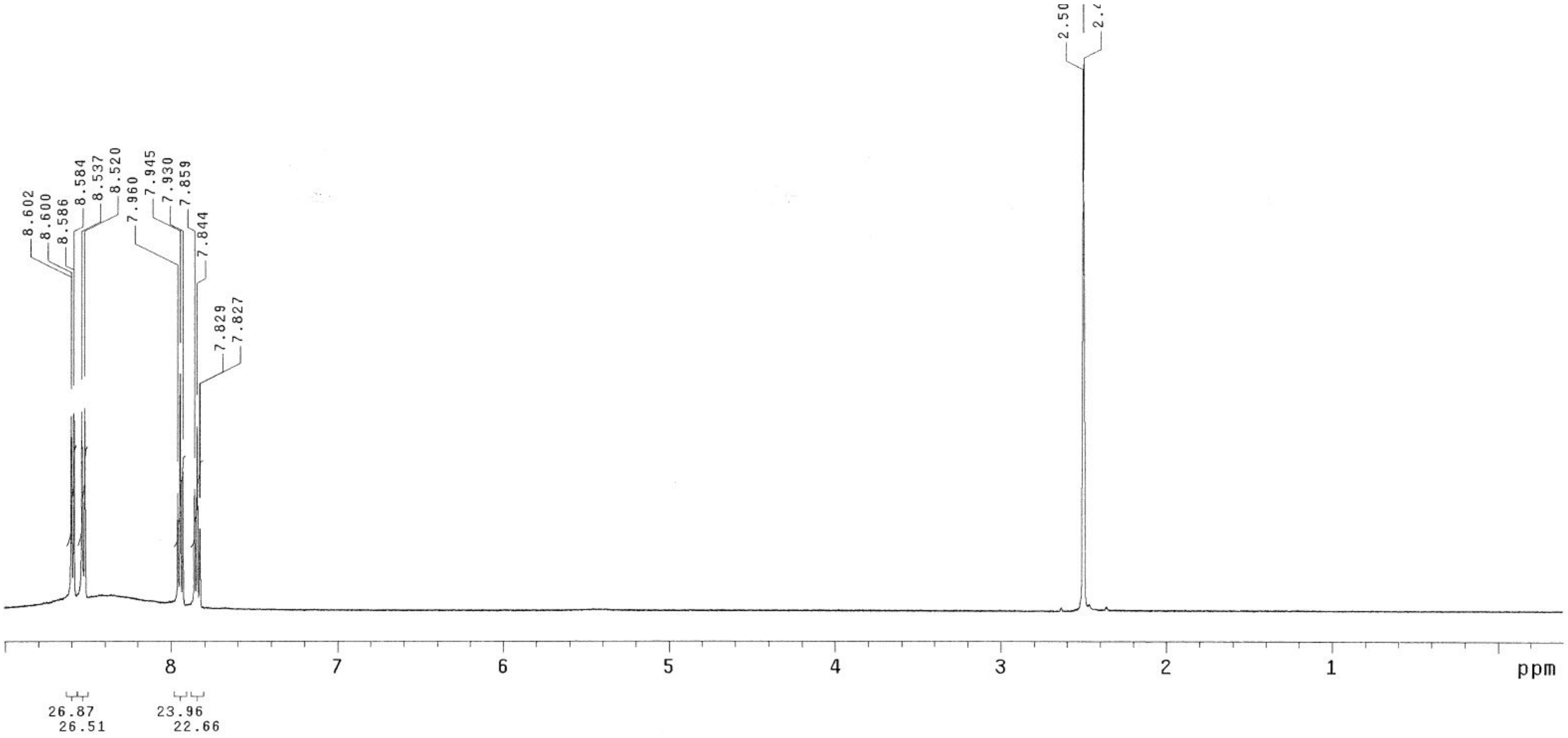

Figure S11. Compound 7d: ^{13}C NMR (75 MHz, DMSO-d$_6$)

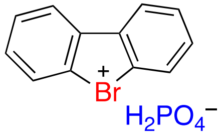

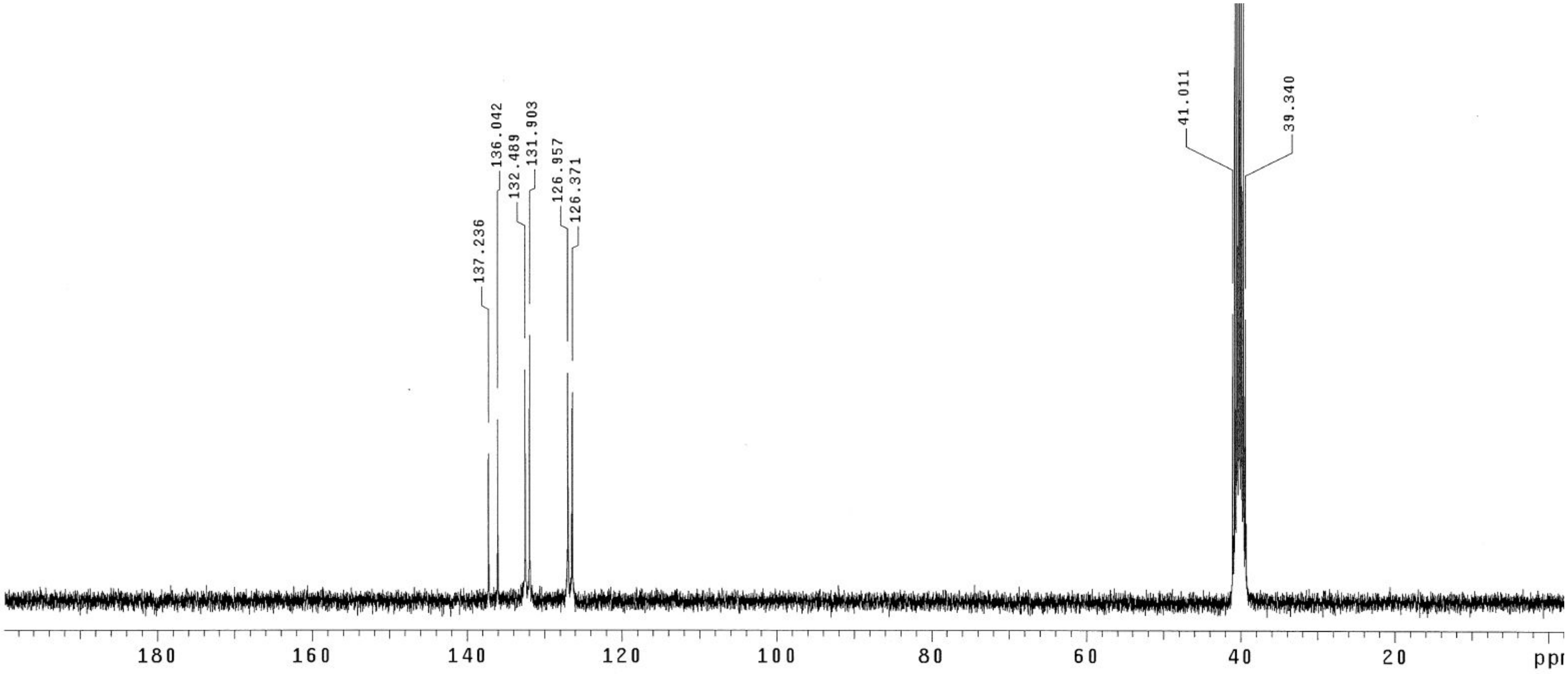

Figure S12. Compound 7d: ^{31}P NMR (121 MHz, DMSO-d_6)

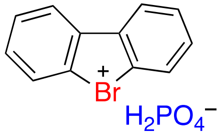

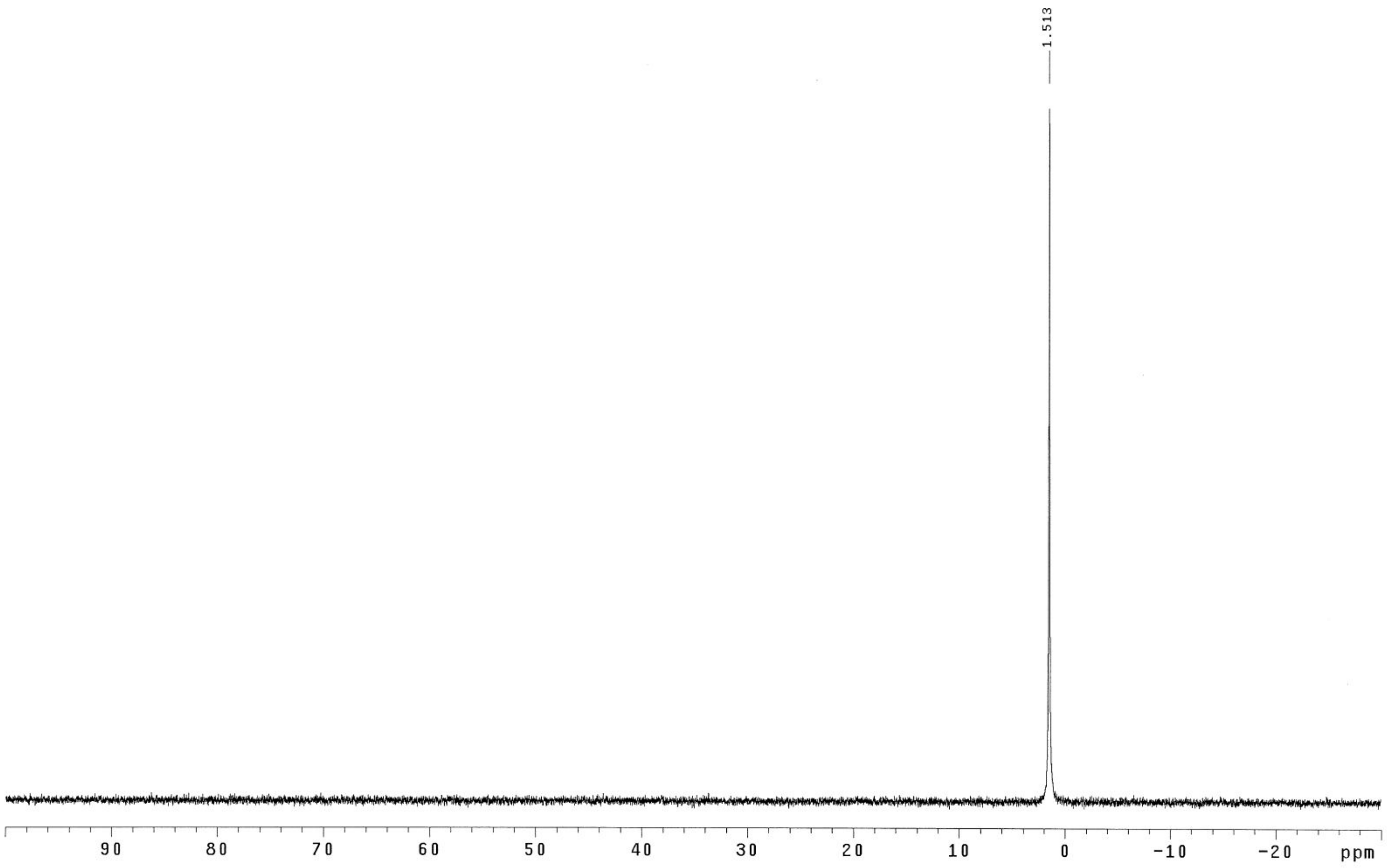

Figure S13. Compound 7e: ^{1}H NMR (400 MHz, CD_3CN)

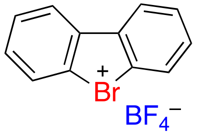

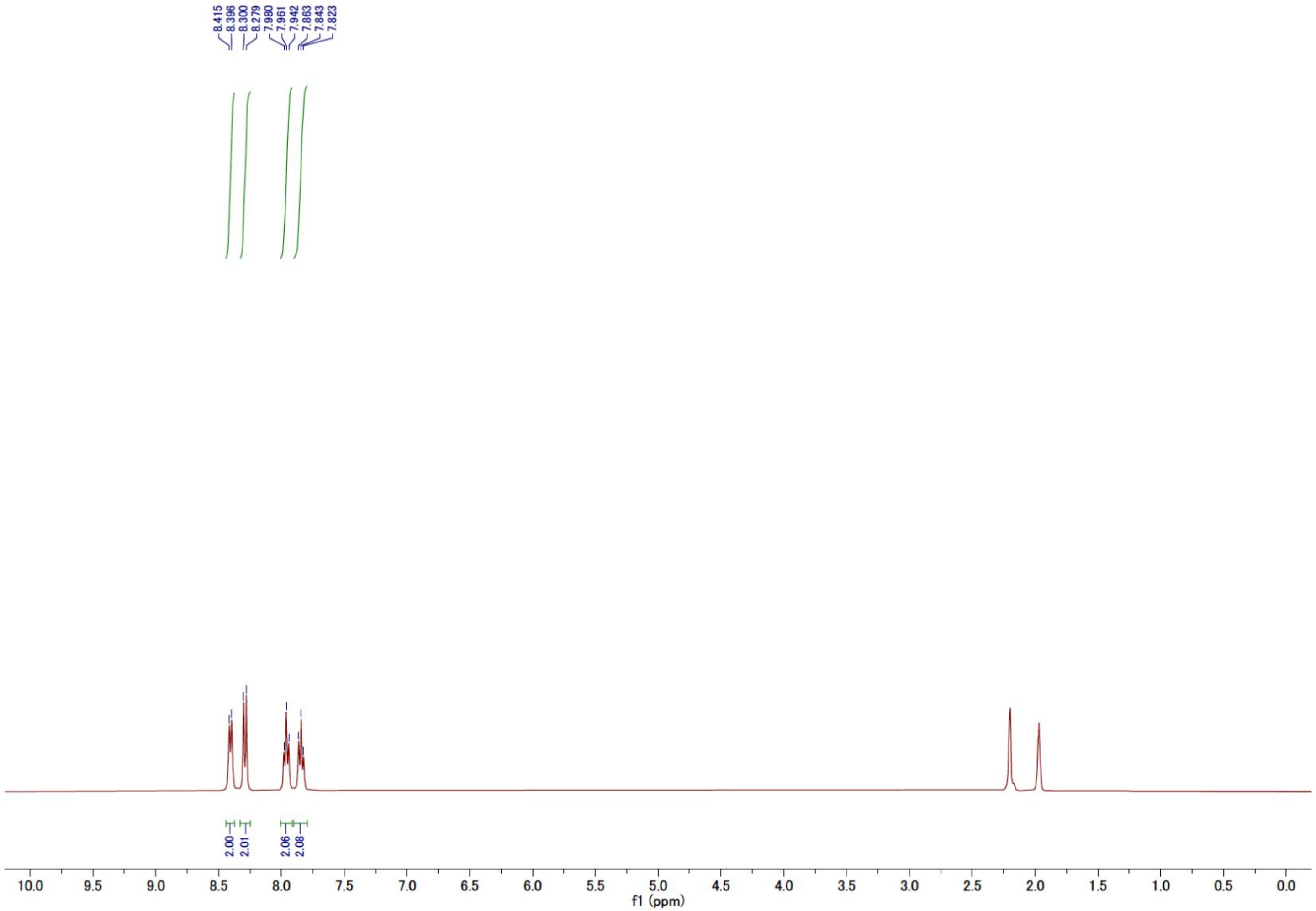

Figure S14. Compound 7e: ^{13}C NMR (100 MHz, CD_3CN)

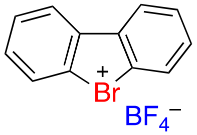

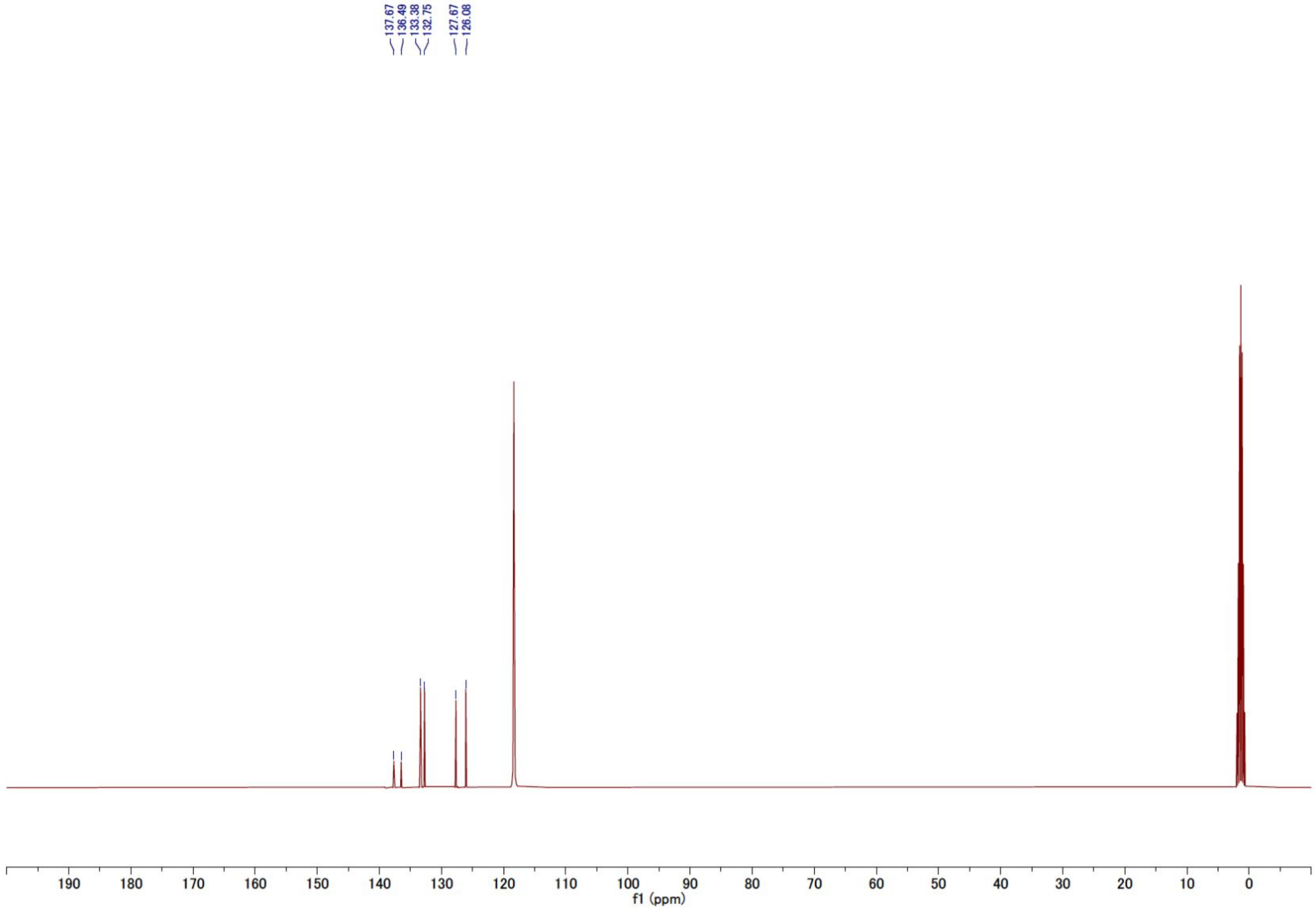

Figure S15. Compound 7e: ^{19}F NMR (376 MHz, CD_3CN)

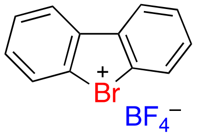

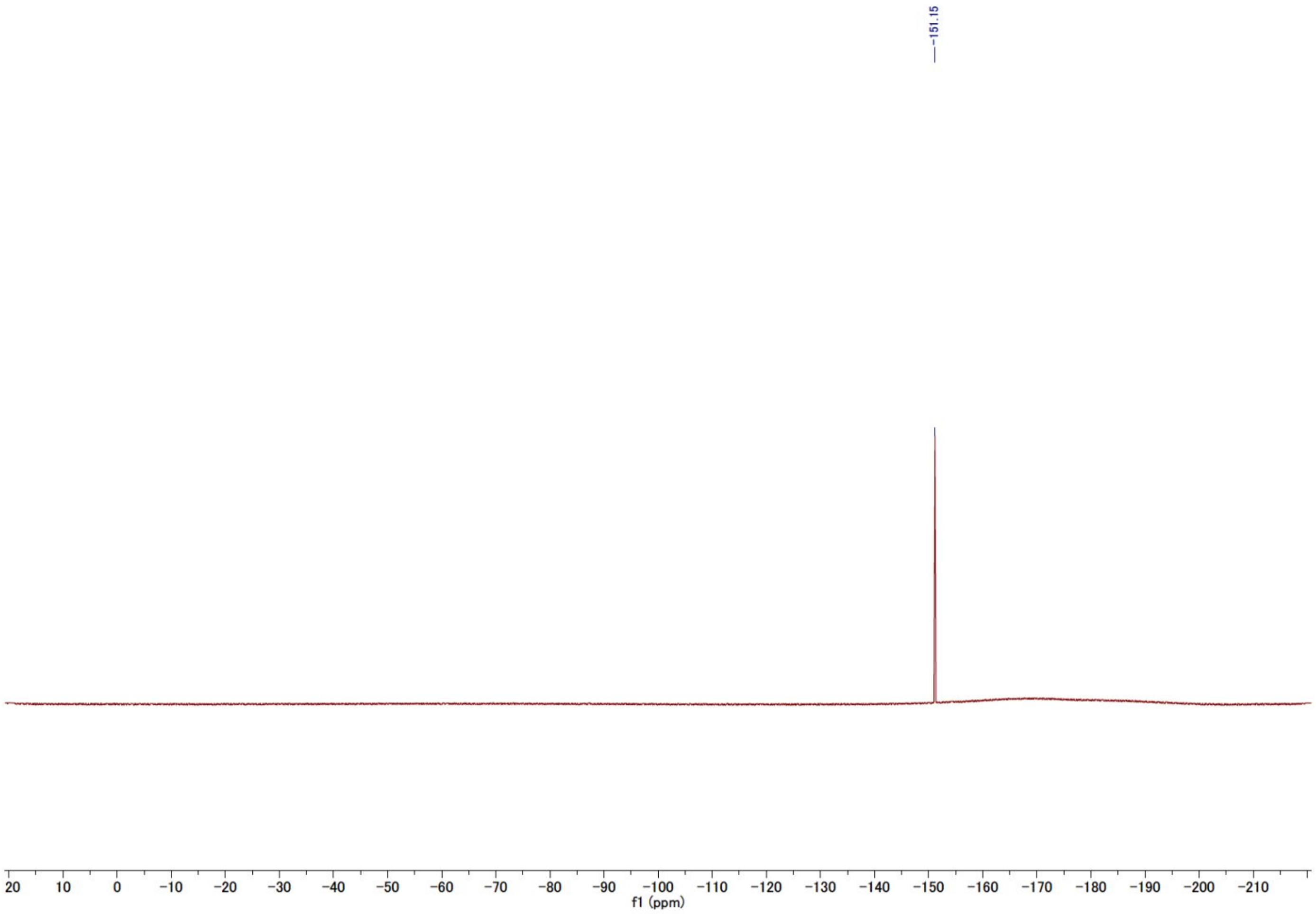

Figure S16. Compound 4: ^{1}H NMR (400 MHz, DMSO-d_6)

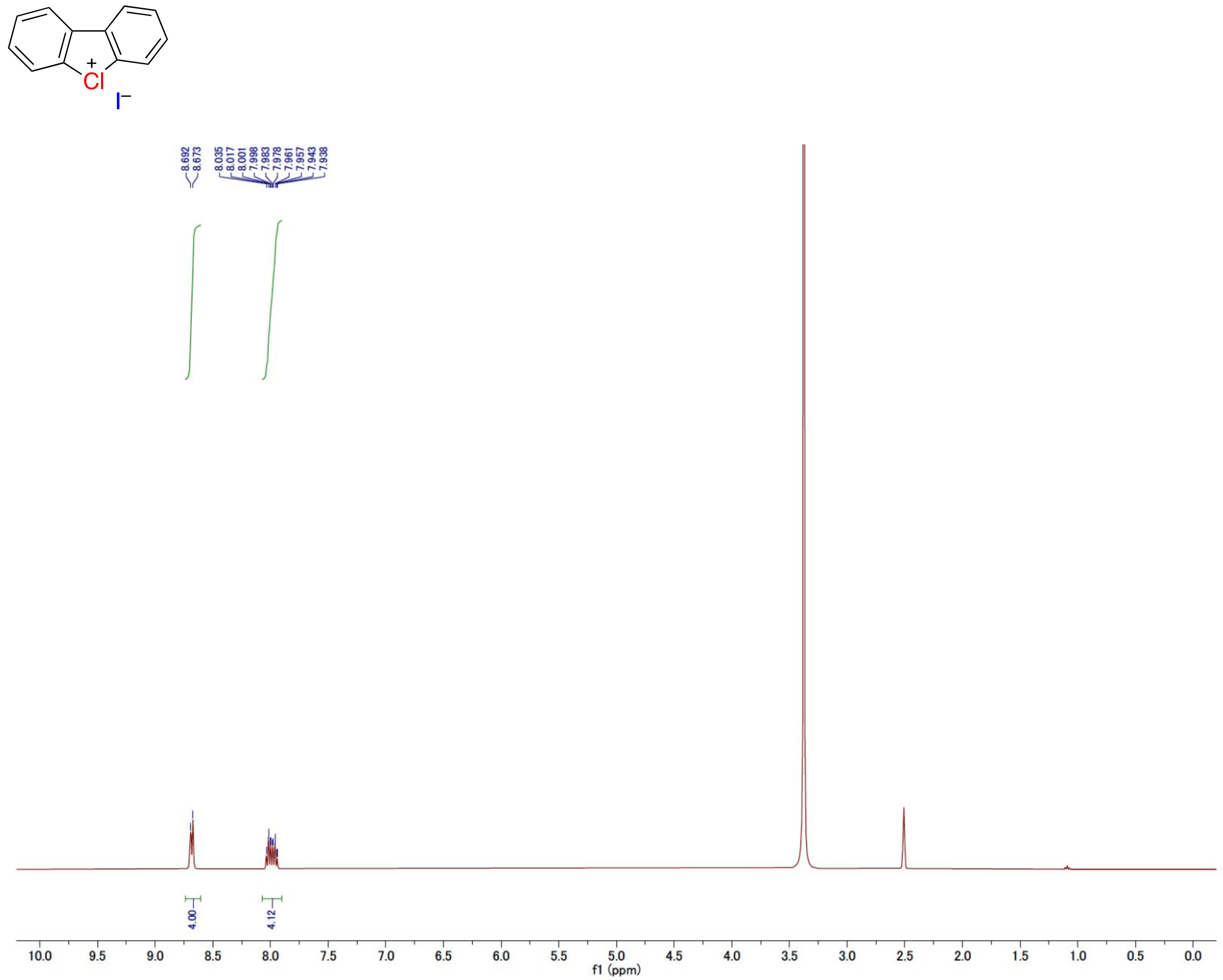

Figure S17. Compound 4: ^{13}C NMR (100 MHz, DMSO-d_6)

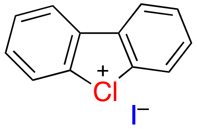

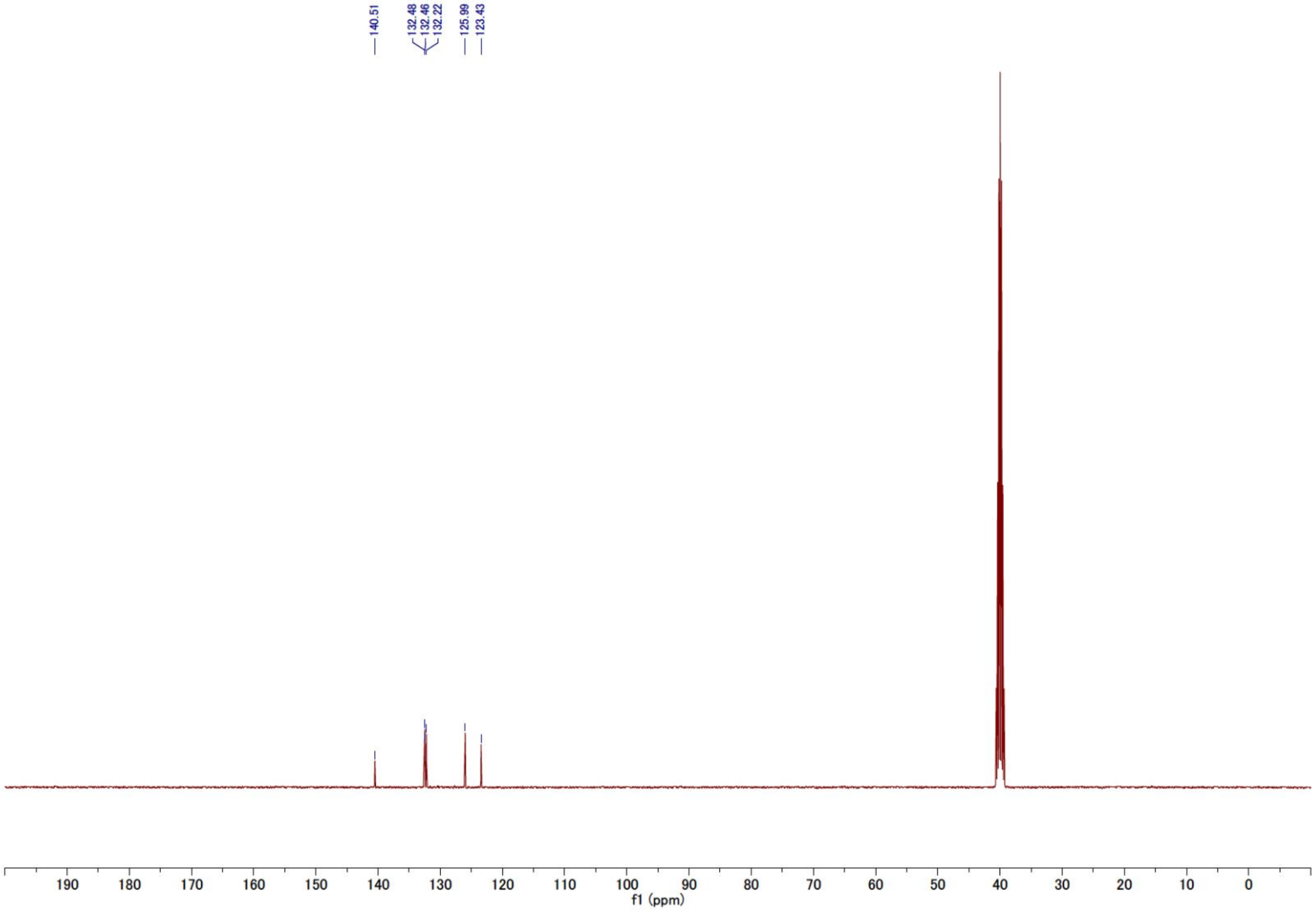

Figure S18. Compound 8a: ^{1}H NMR (400 MHz, CD_3CN)

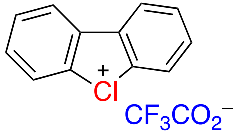

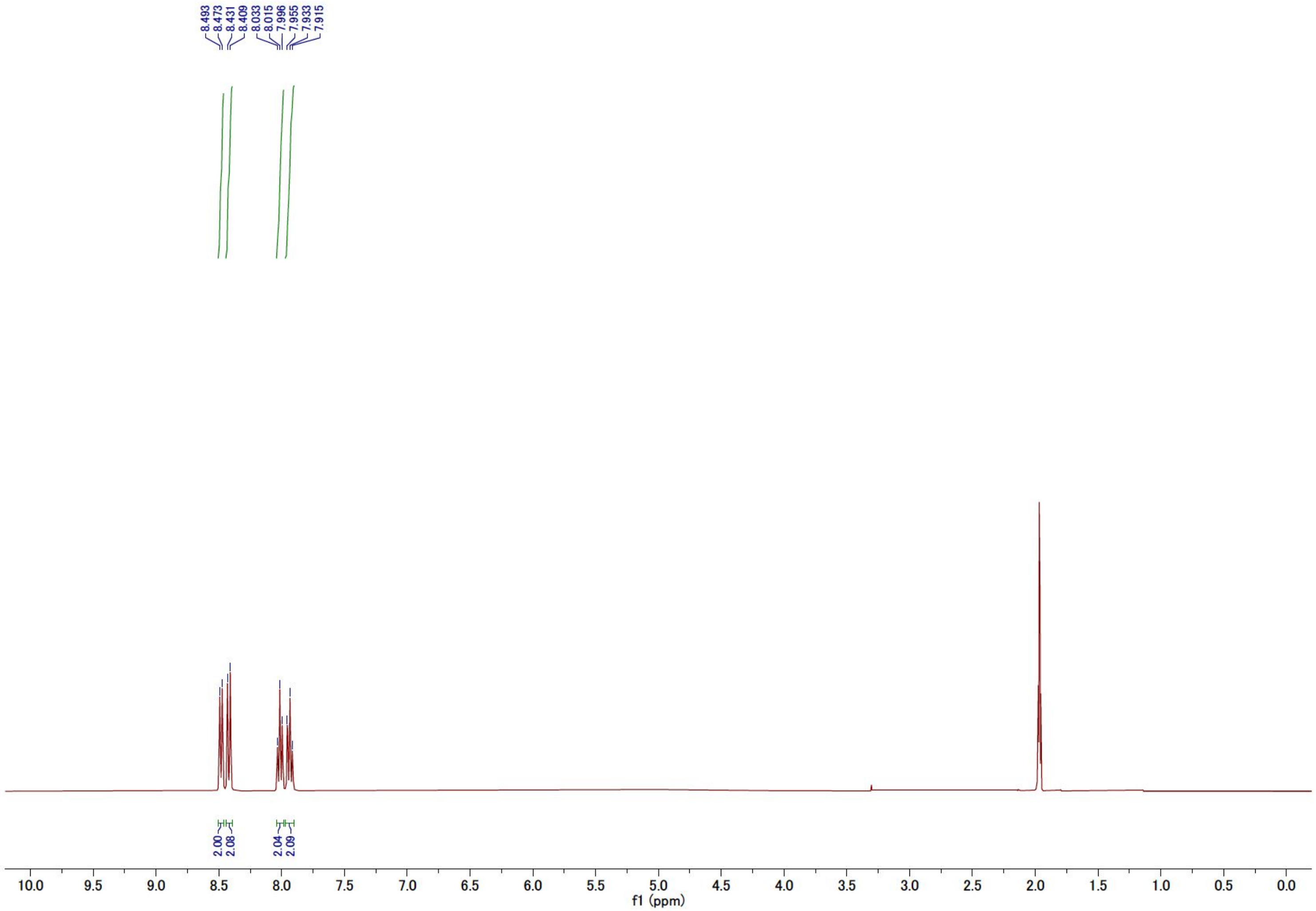

Figure S19. Compound 8a: ^{13}C NMR (100 MHz, CD_3CN)

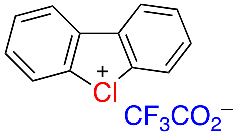

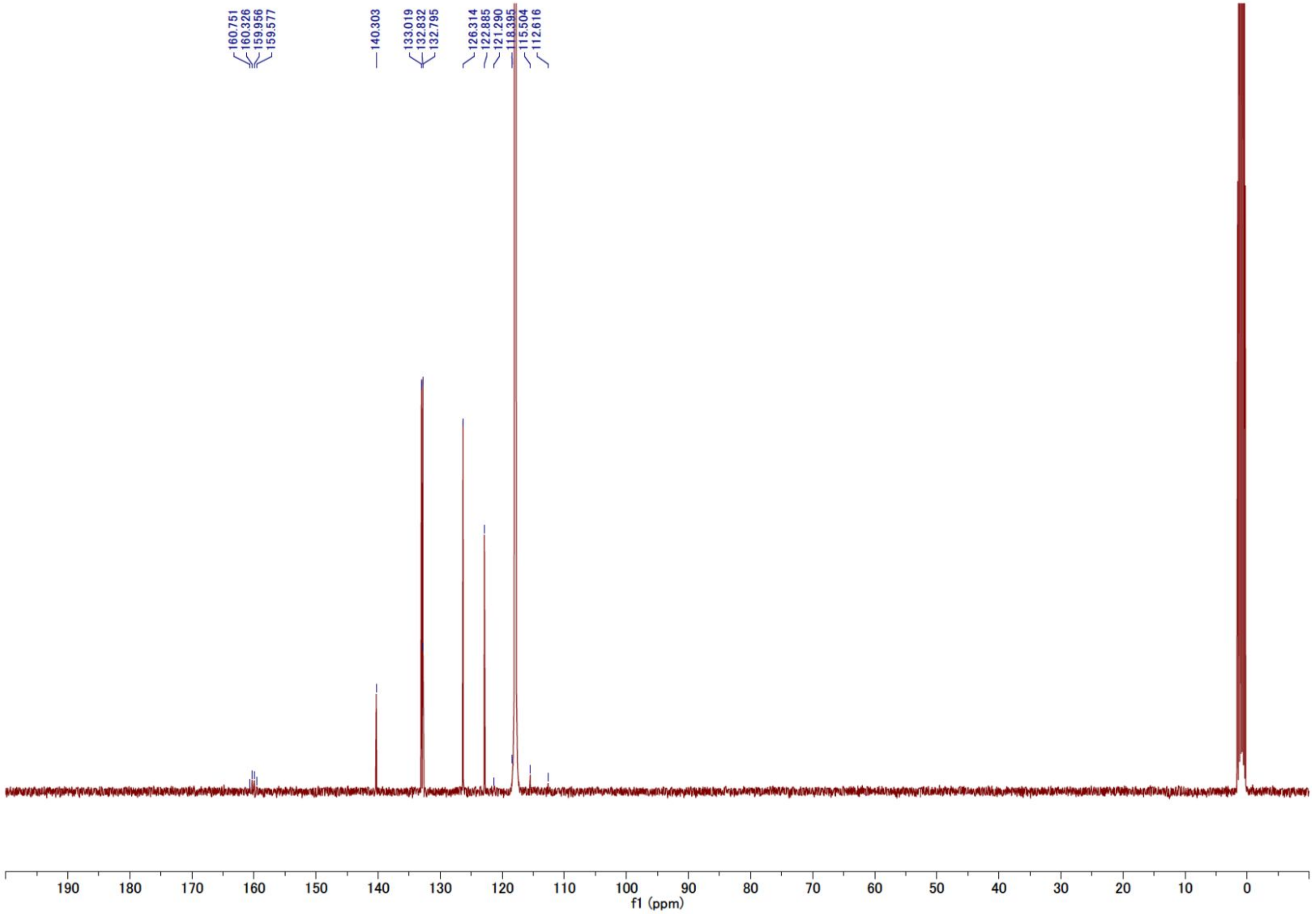

Figure S20. Compound 8a: ^{19}F NMR (377 MHz, CD_3CN)

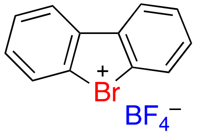

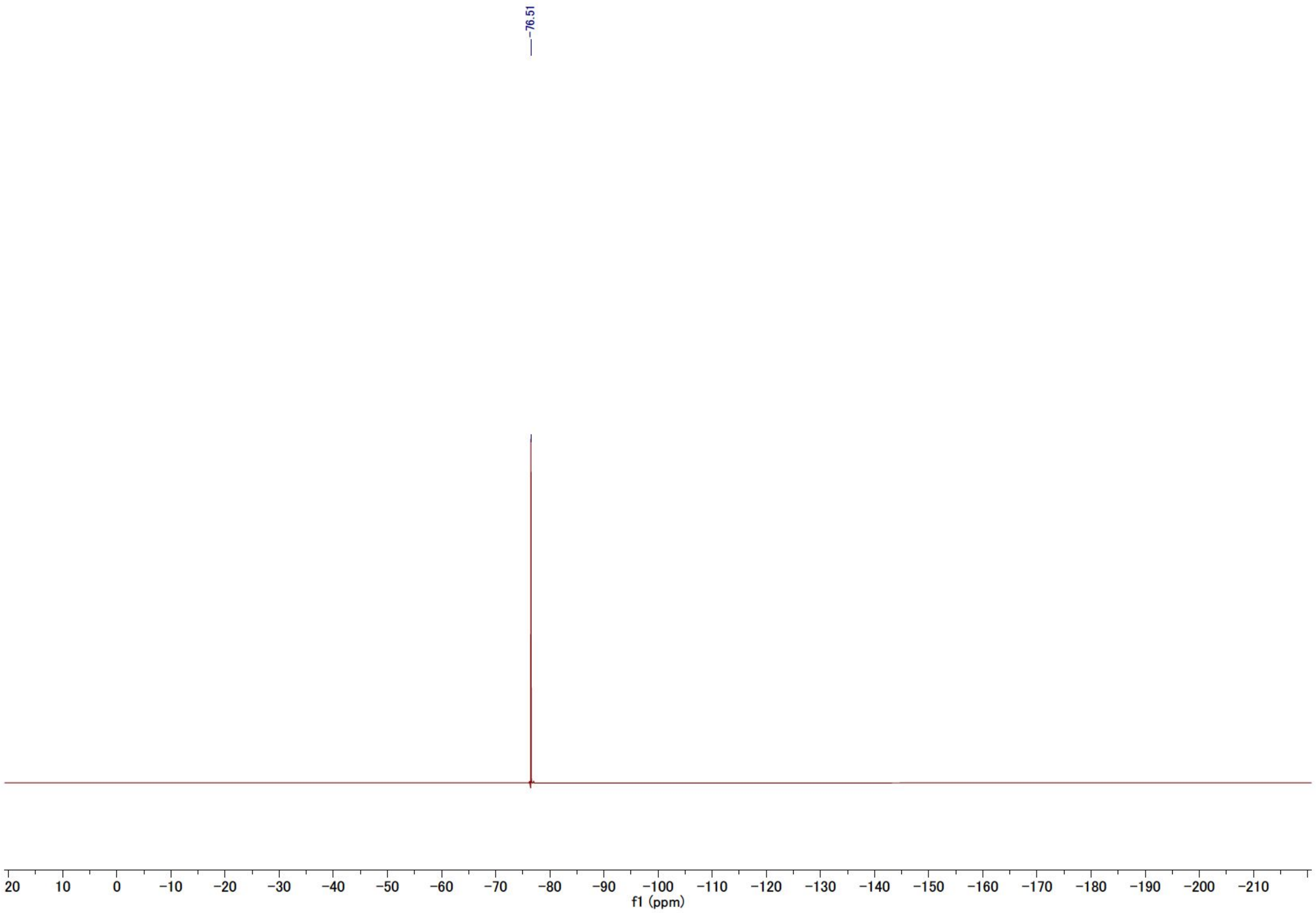

Figure S21. Compound 8b: ^{1}H NMR (400 MHz, CD_3OD)

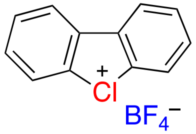

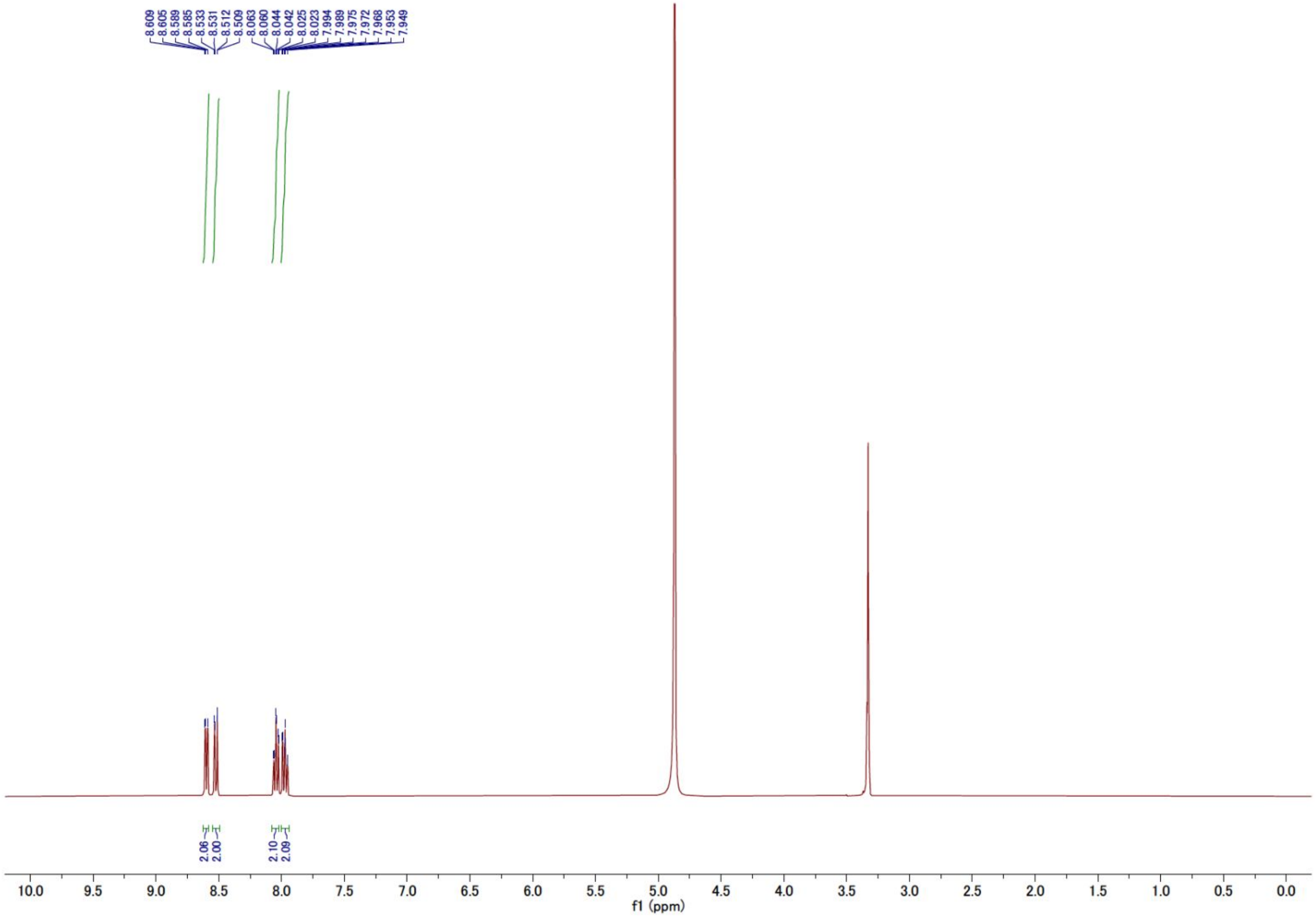

Figure S22. Compound 8b: ^{13}C NMR (100 MHz, CD_3OD)

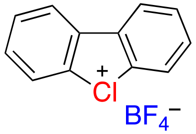

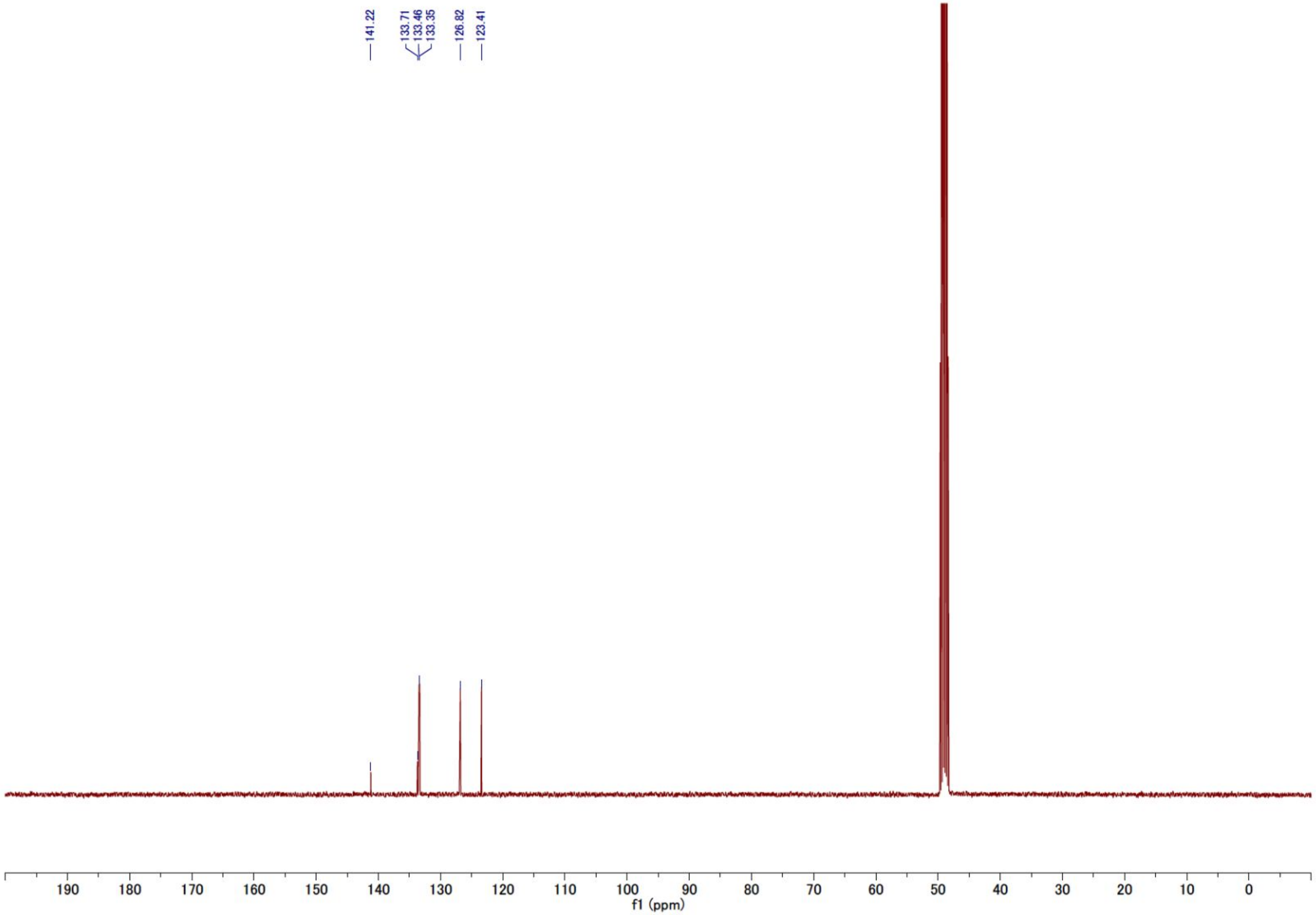

Figure S23. Compound 8b: ^{19}F NMR (377 MHz, CD_3OD)

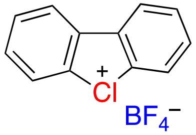

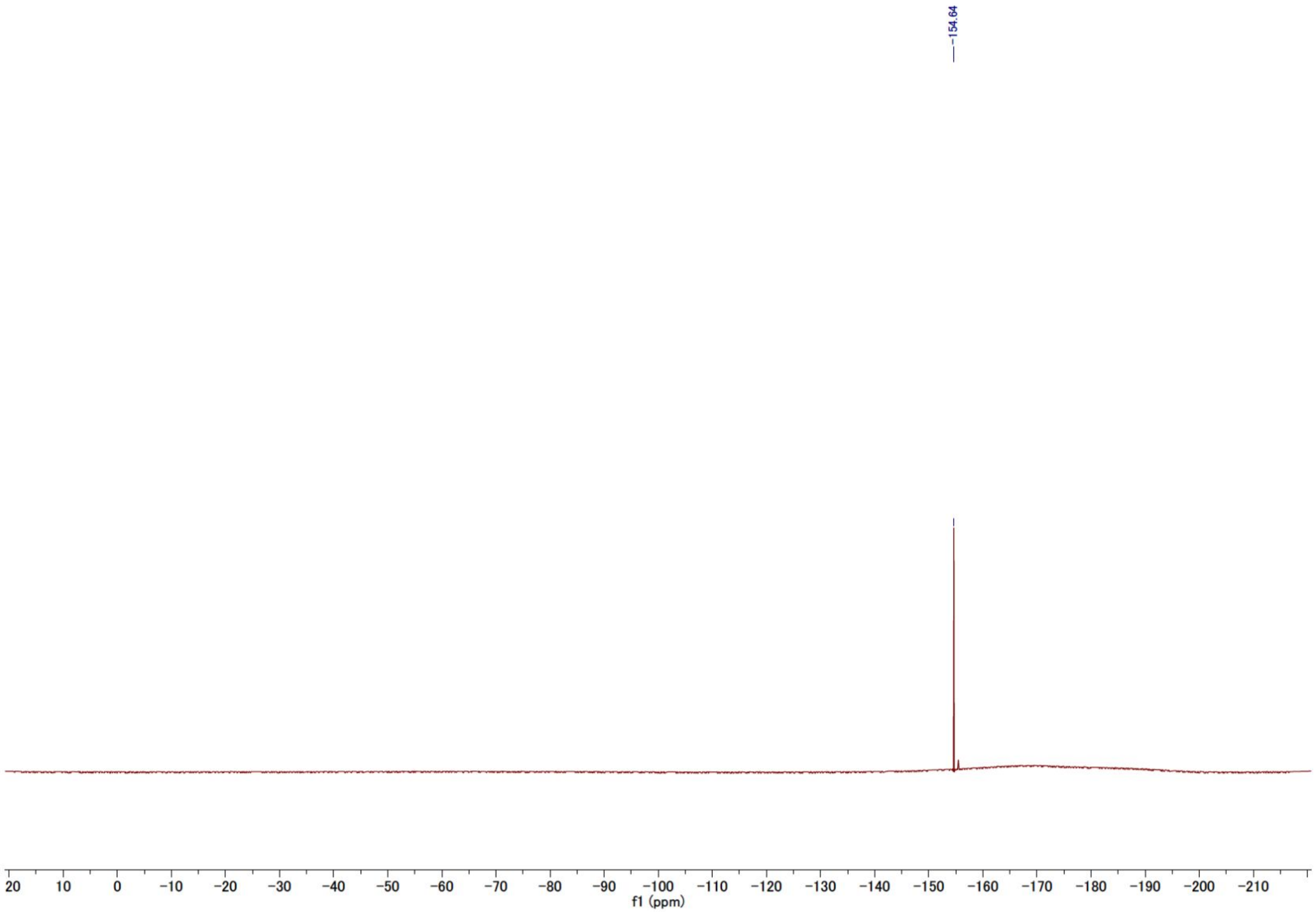

Figure S24. Compound 7f: ^{1}H NMR (400 MHz, CD_3OD)

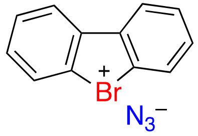

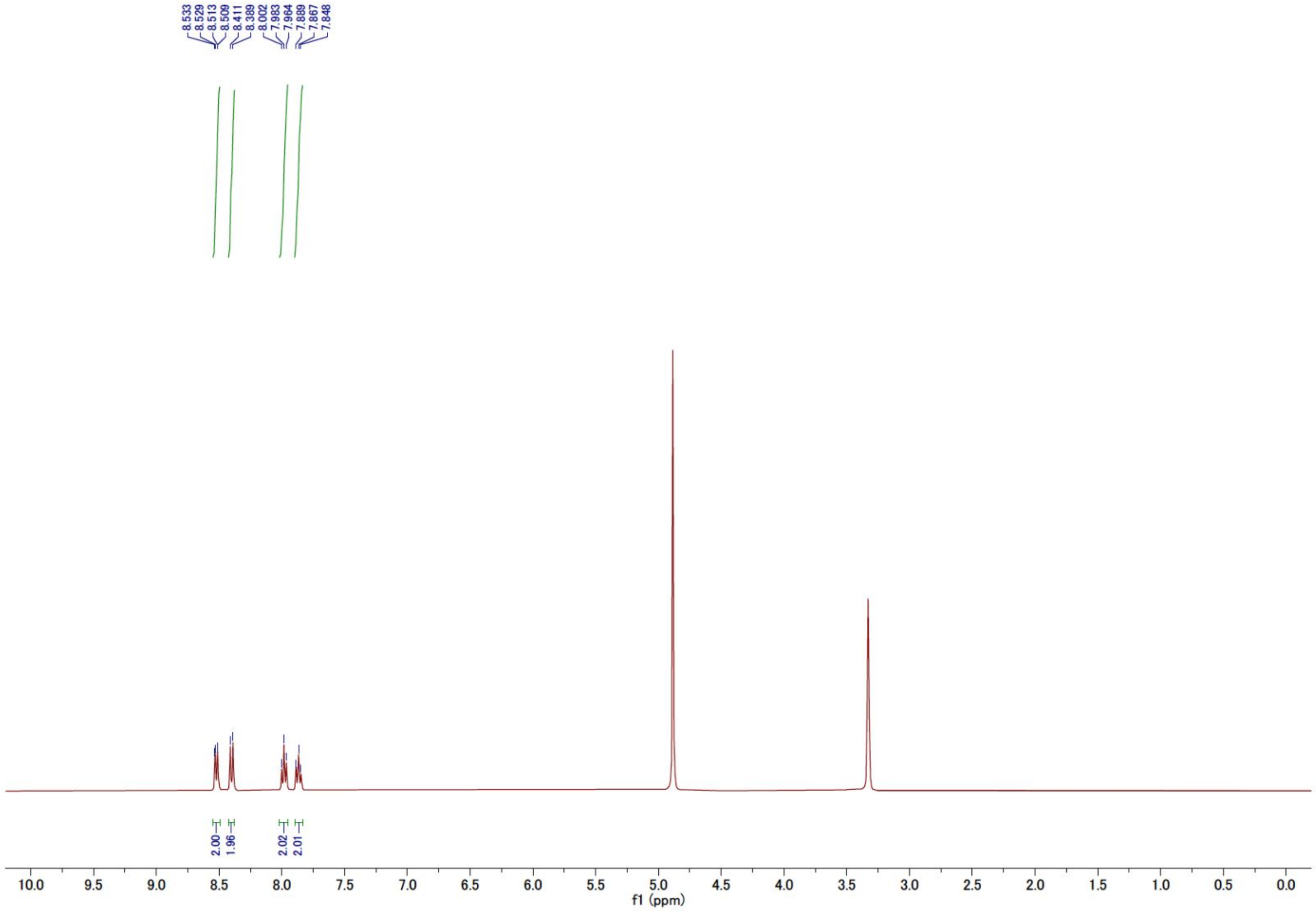

Figure S25. Compound 7f: ^{13}C NMR (75 MHz, CD_3OD)

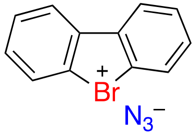

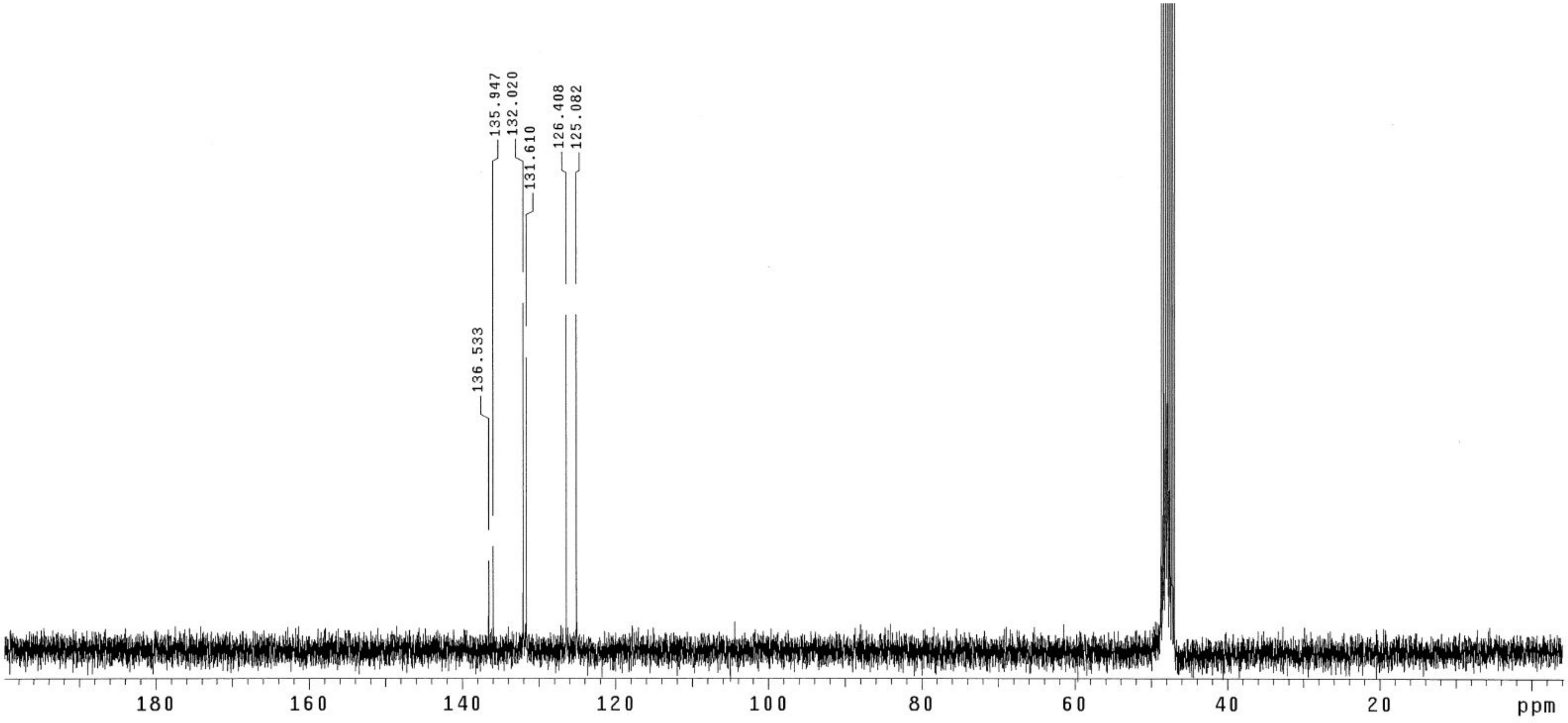

Figure S26. Compound 7g: ^{1}H NMR (400 MHz, CD_3CN)

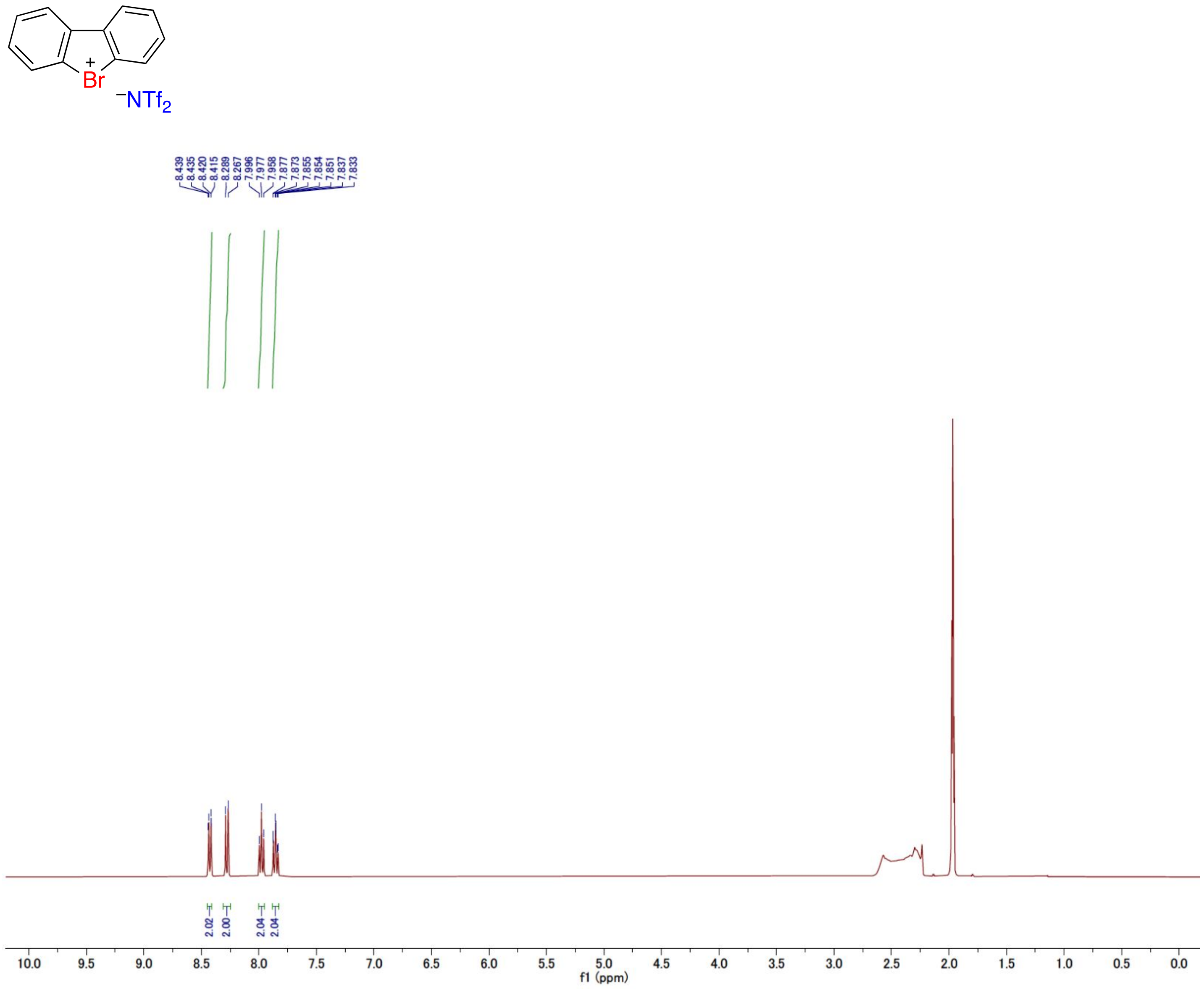

Figure S27. Compound 7g: ^{13}C NMR (100 MHz, CD_3CN)

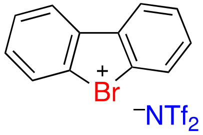

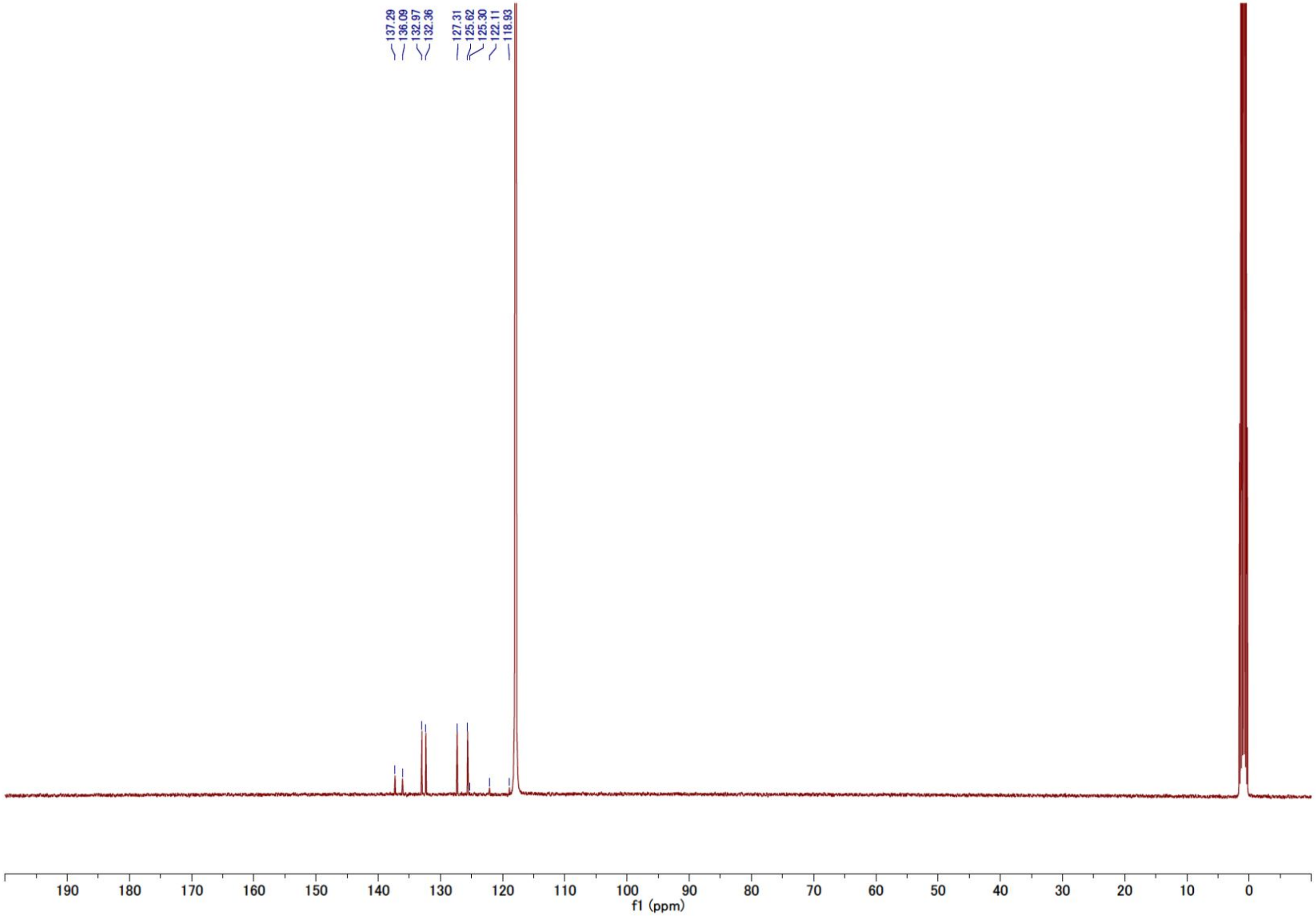

Figure S28. Compound 7g: ^{19}F NMR (376 MHz, CD_3CN)

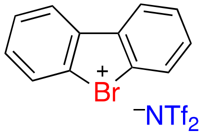

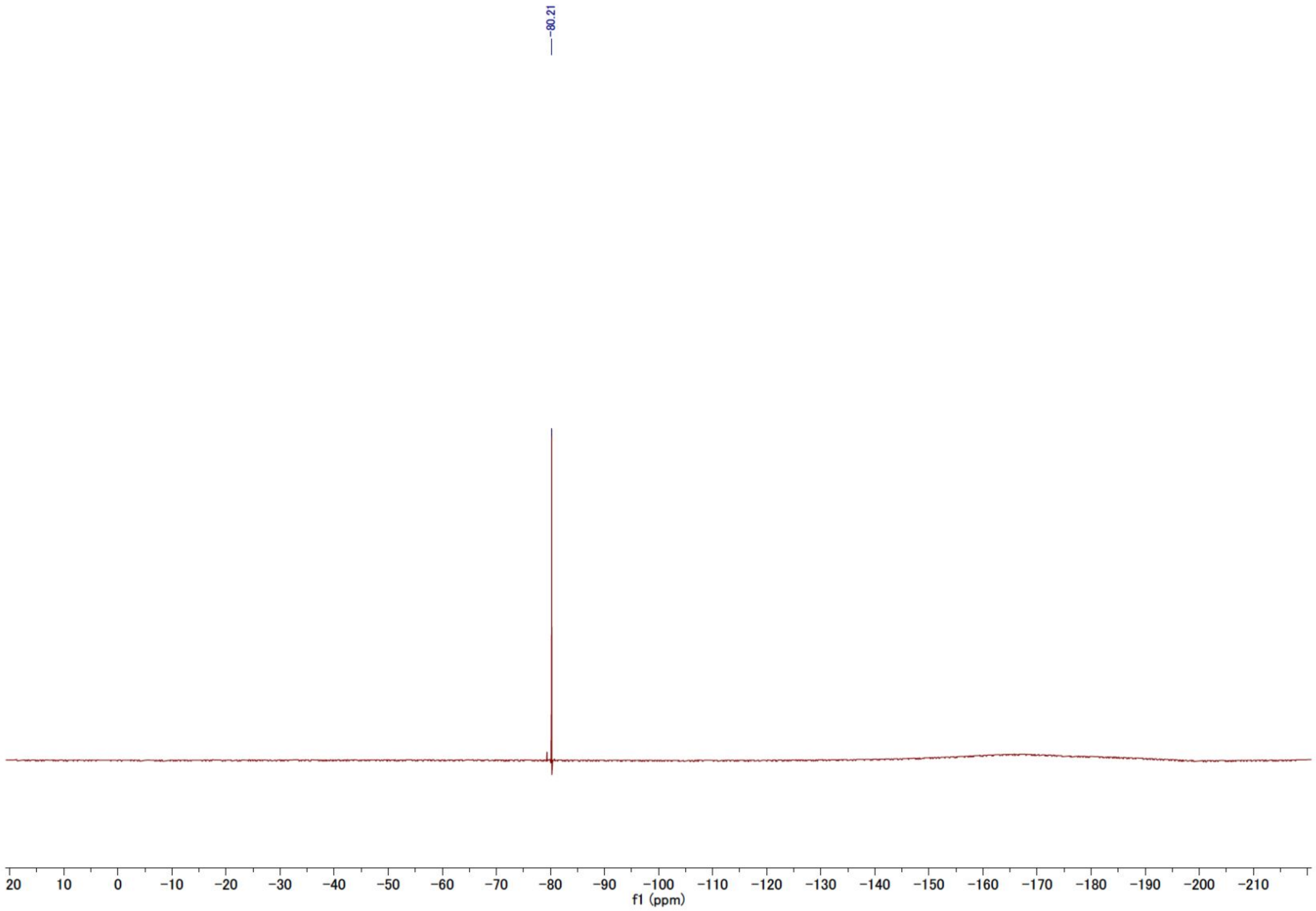

Figure S29. Compound 7h: ^{1}H NMR (400 MHz, CD_3CN)

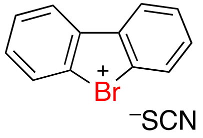

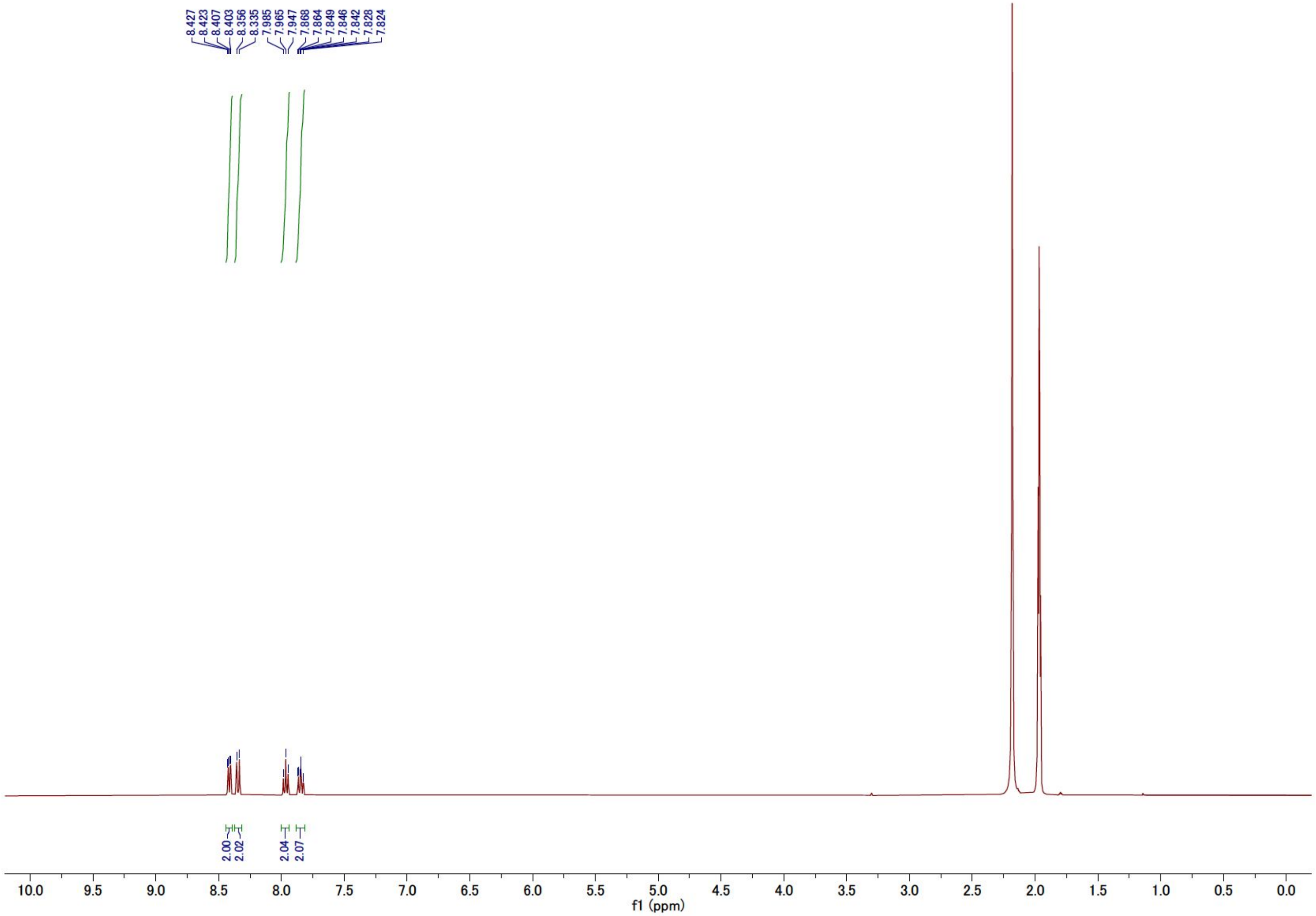

Figure S30. Compound 7h: ^{13}C NMR (100 MHz, CD_3CN)

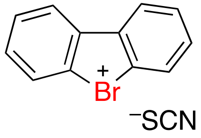

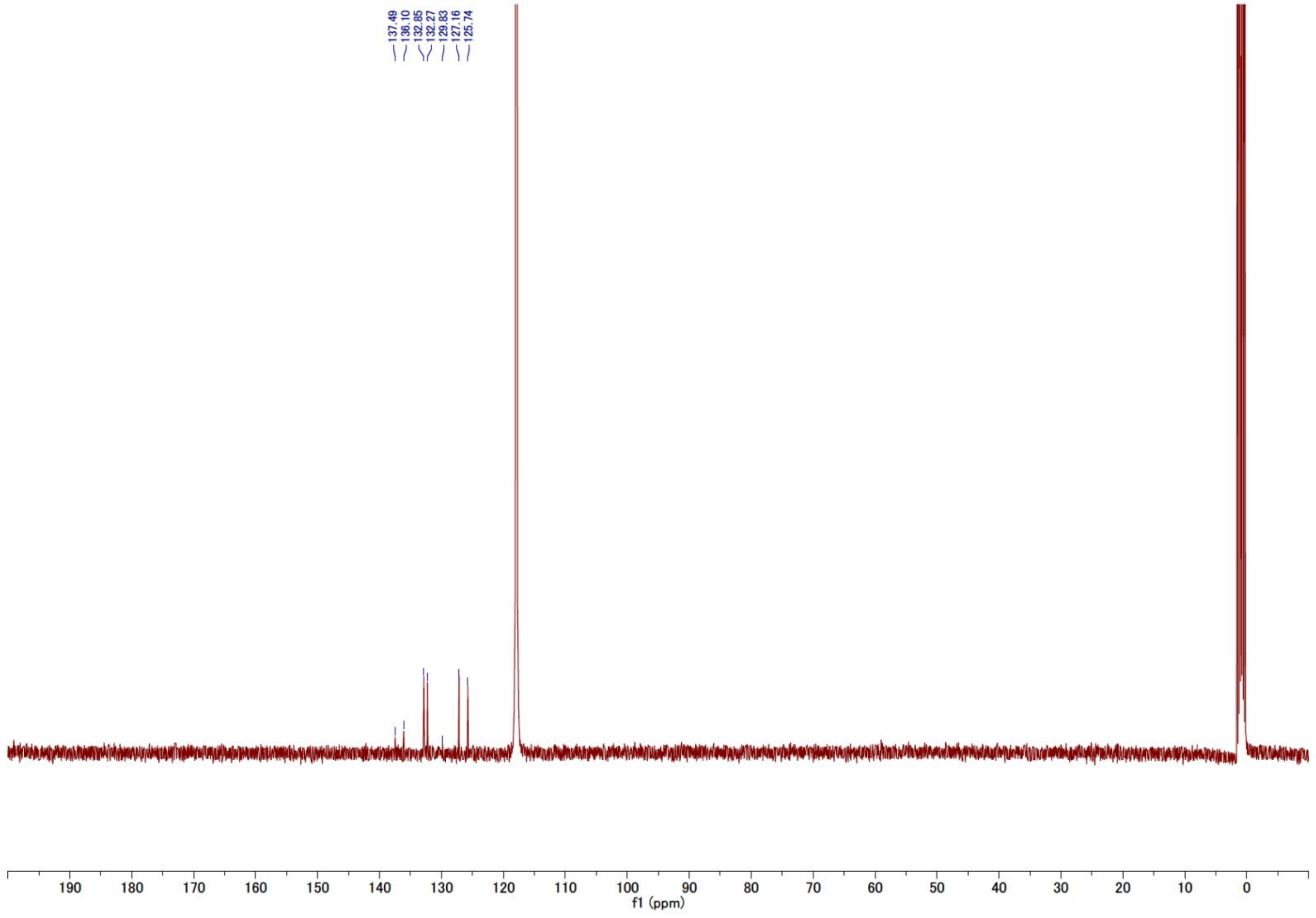

Figure S31. Compound 7i: ^{1}H NMR (400 MHz, CD_3CN)

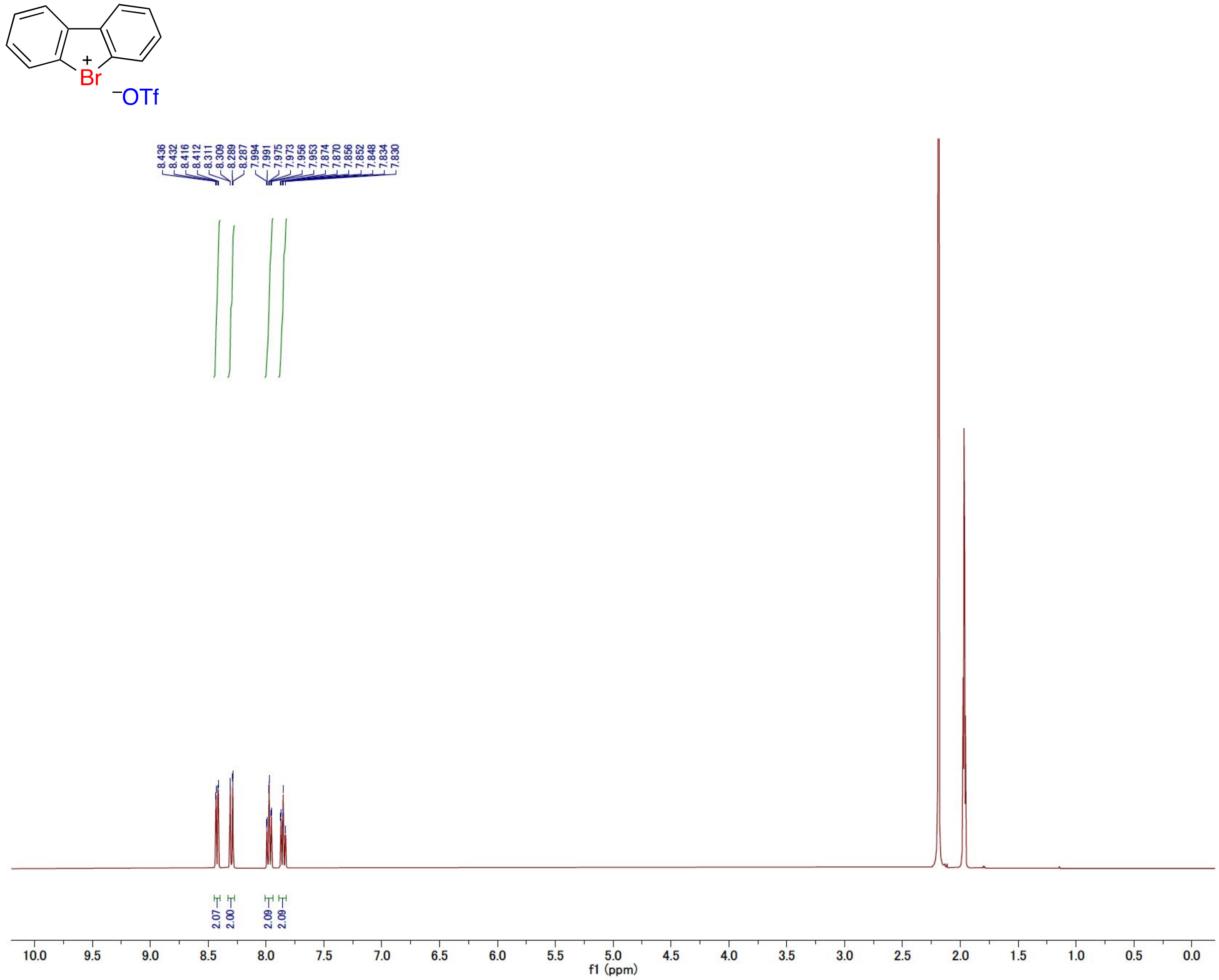

Figure S32. Compound 7i: ^{13}C NMR (100 MHz, CD_3CN)

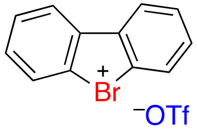

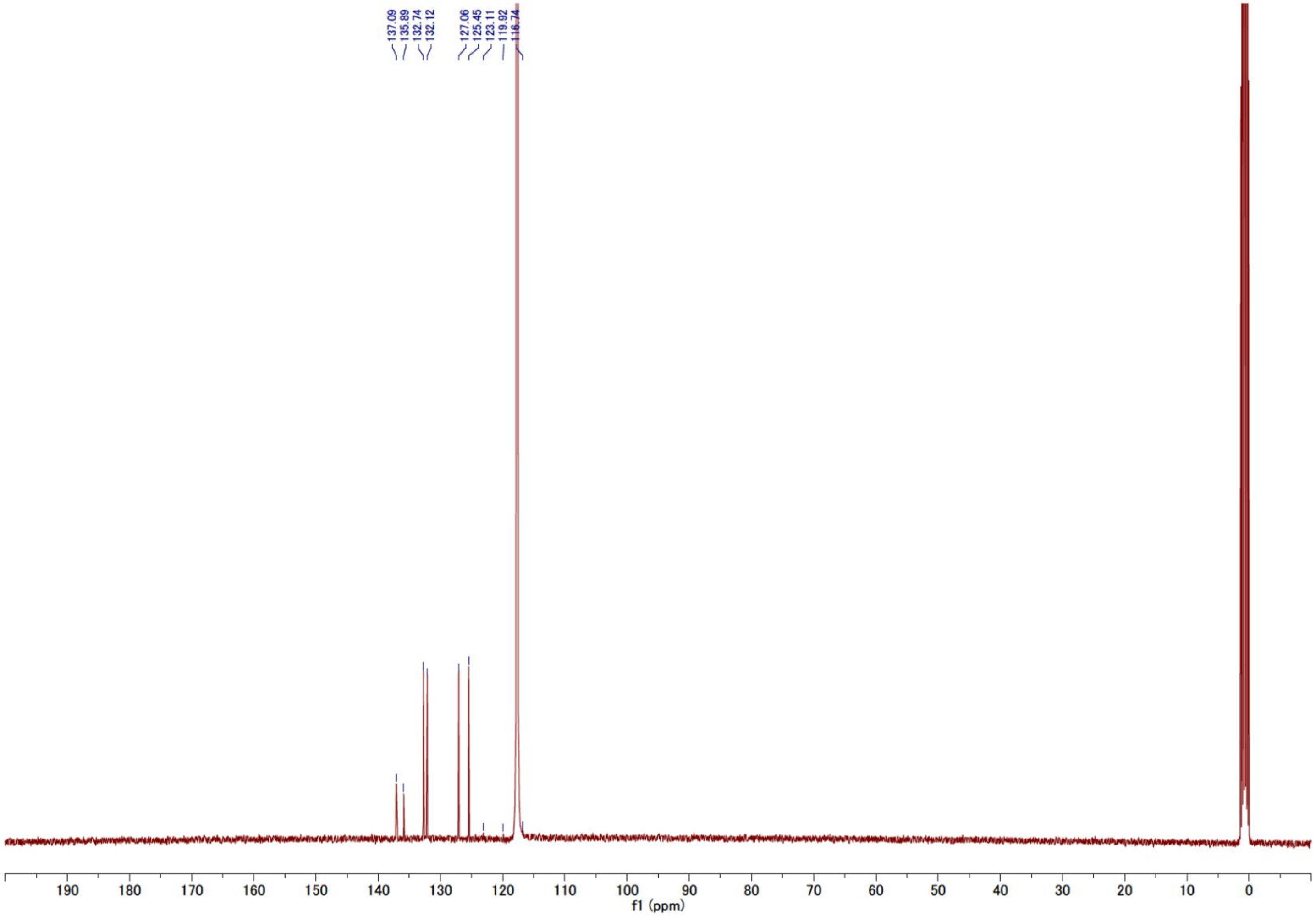

Figure S33. Compound 7i: ^{19}F NMR (376 MHz, CD_3CN)

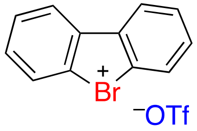

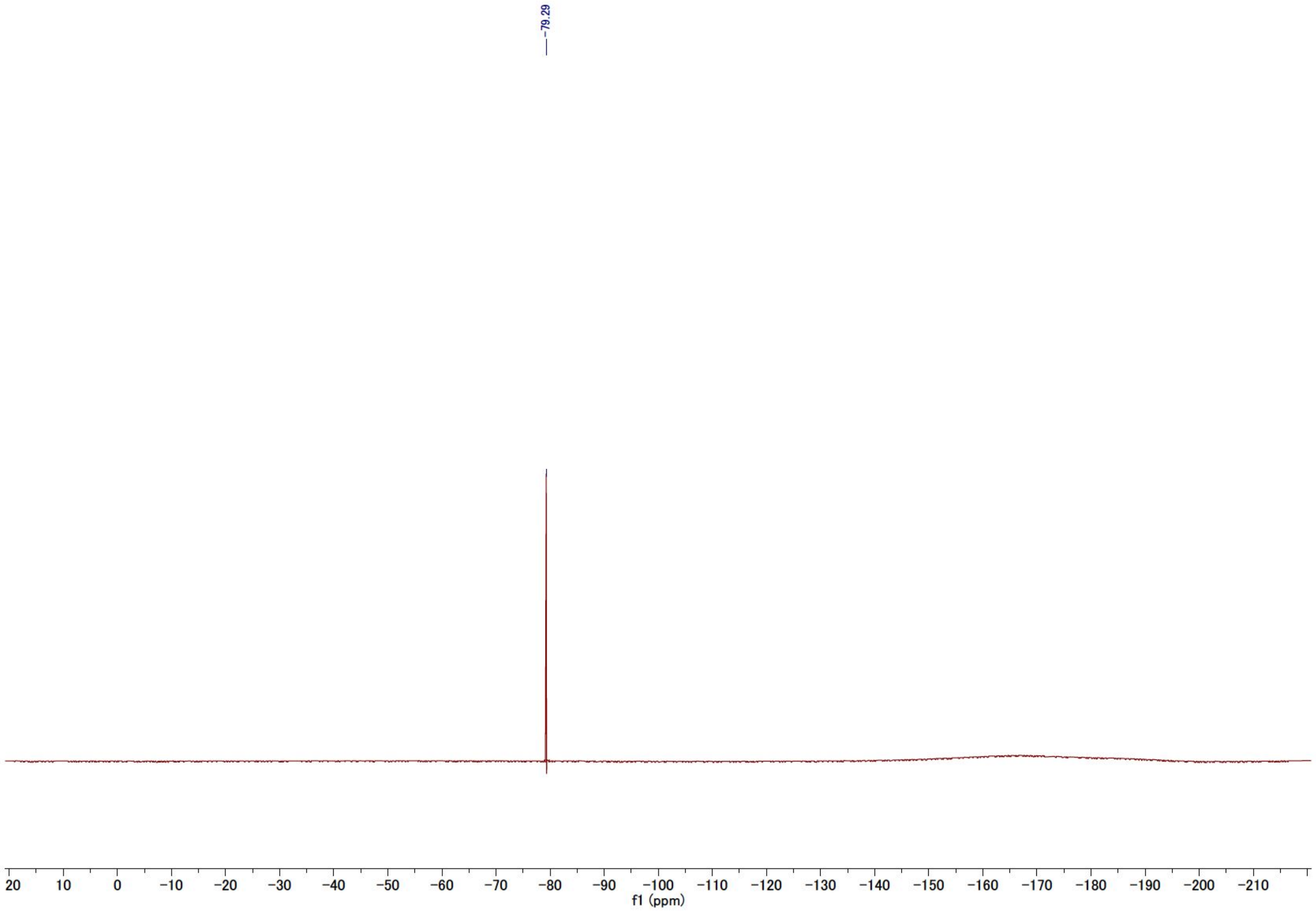

Figure S34. Compound 8c: ^{1}H NMR (400 MHz, CD_3OD)

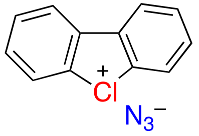

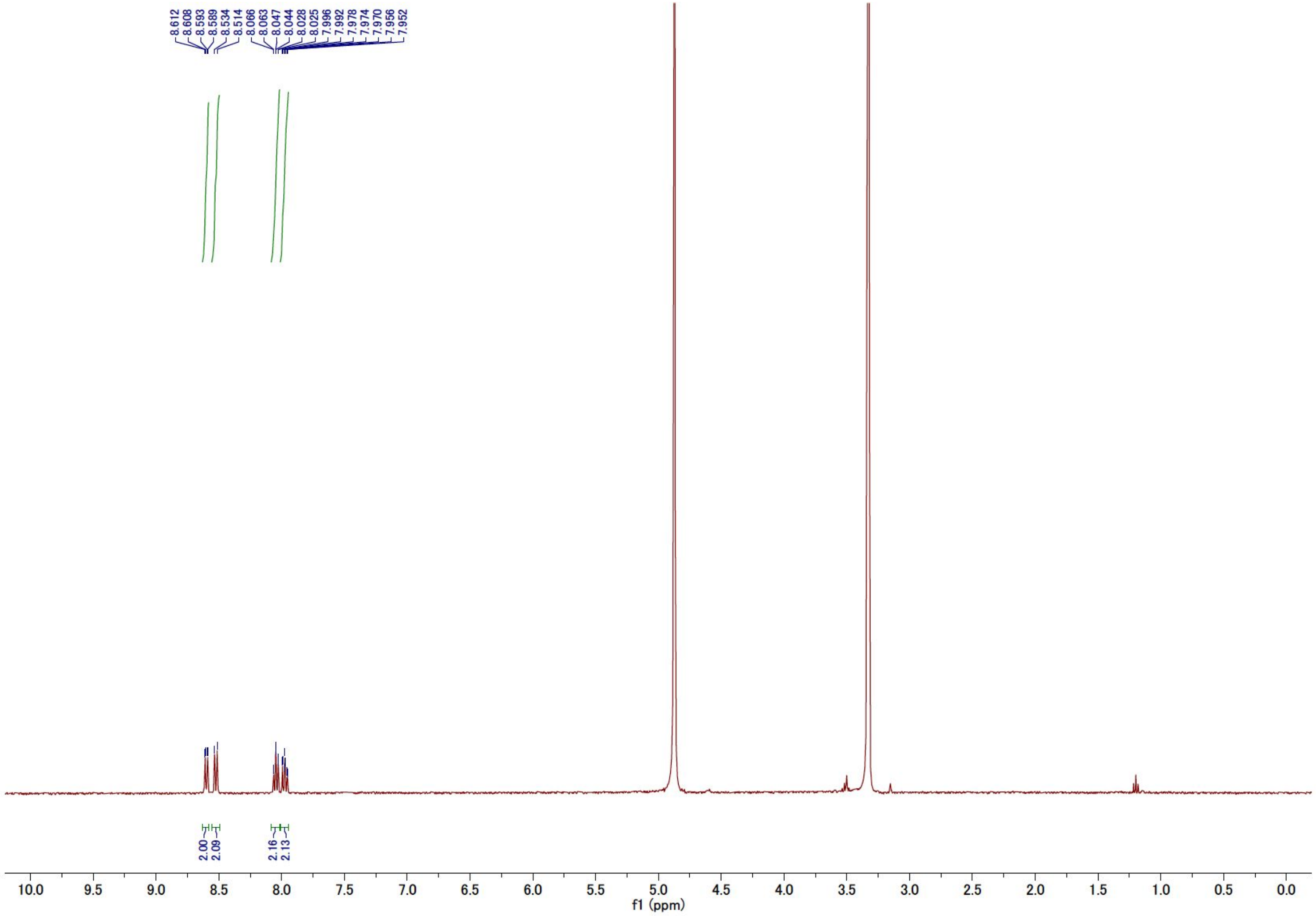

Figure S35. Compound 8c: ^{13}C NMR (100 MHz, CD_3OD)

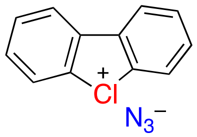

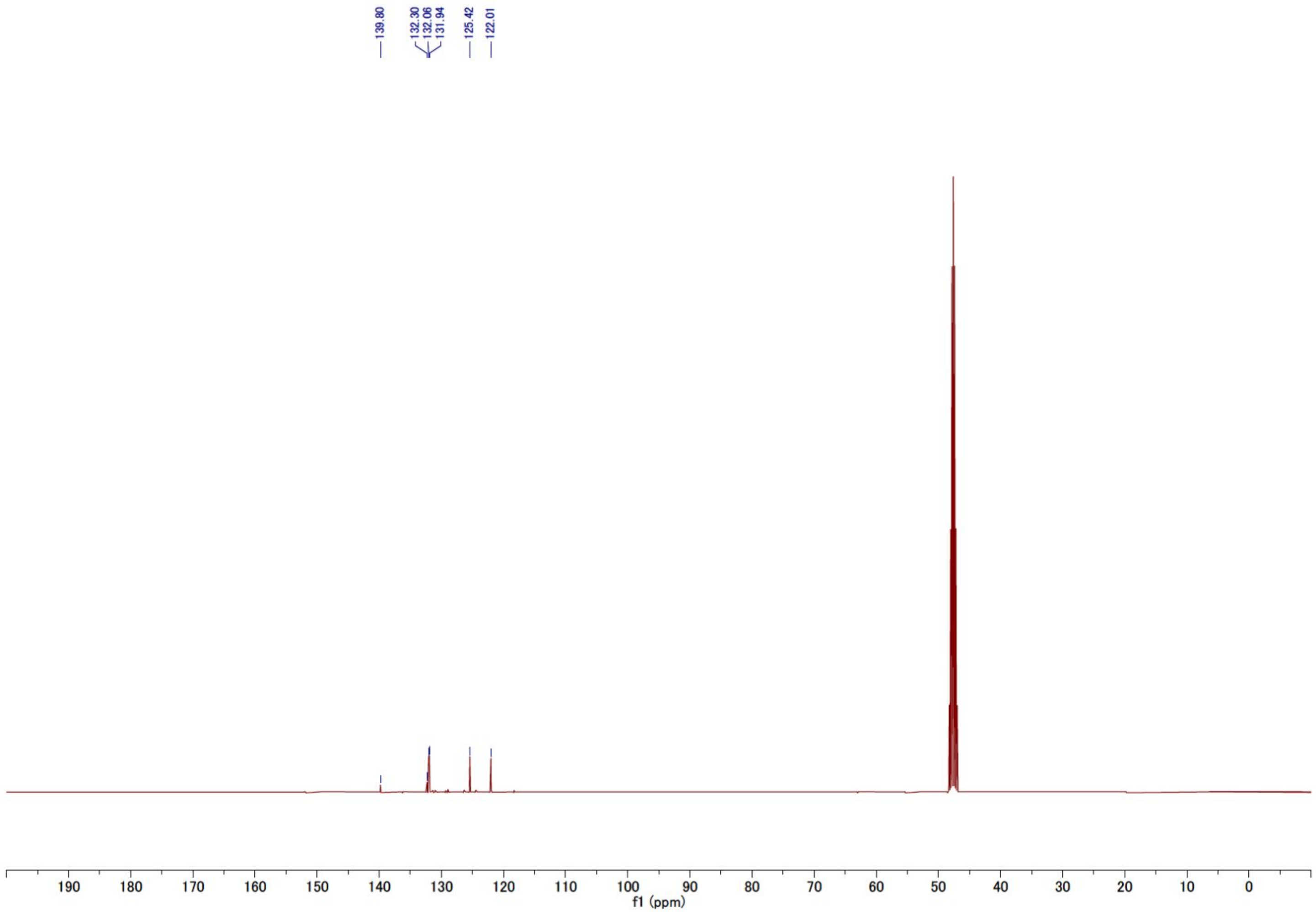

Figure S36. Compound 8d: ^{1}H NMR (400 MHz, CD_3CN)

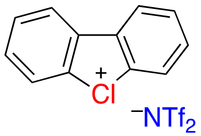

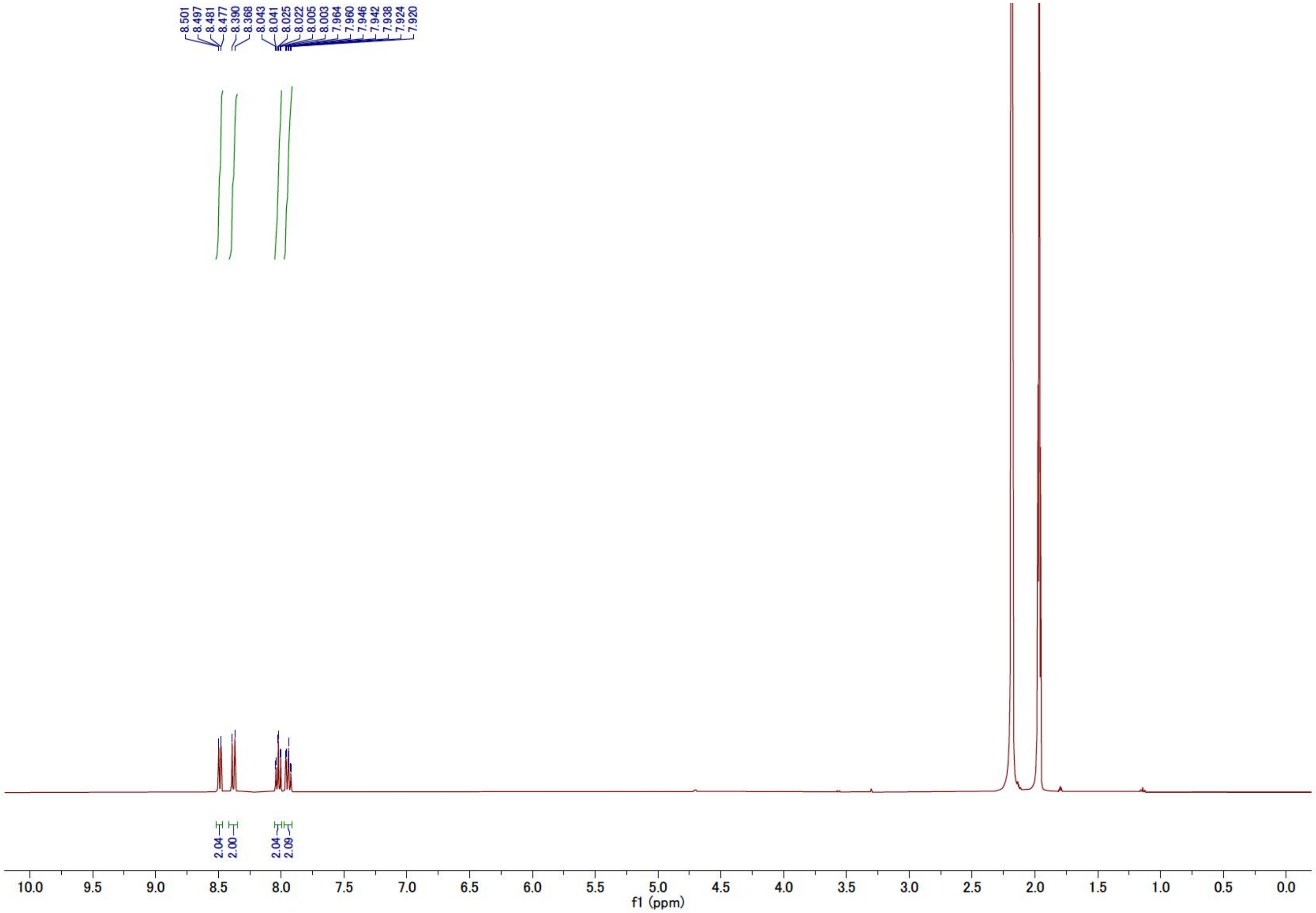

Figure S37. Compound 8d: ^{13}C NMR (75 MHz, CD_3OD)

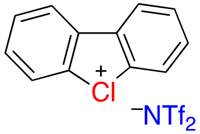

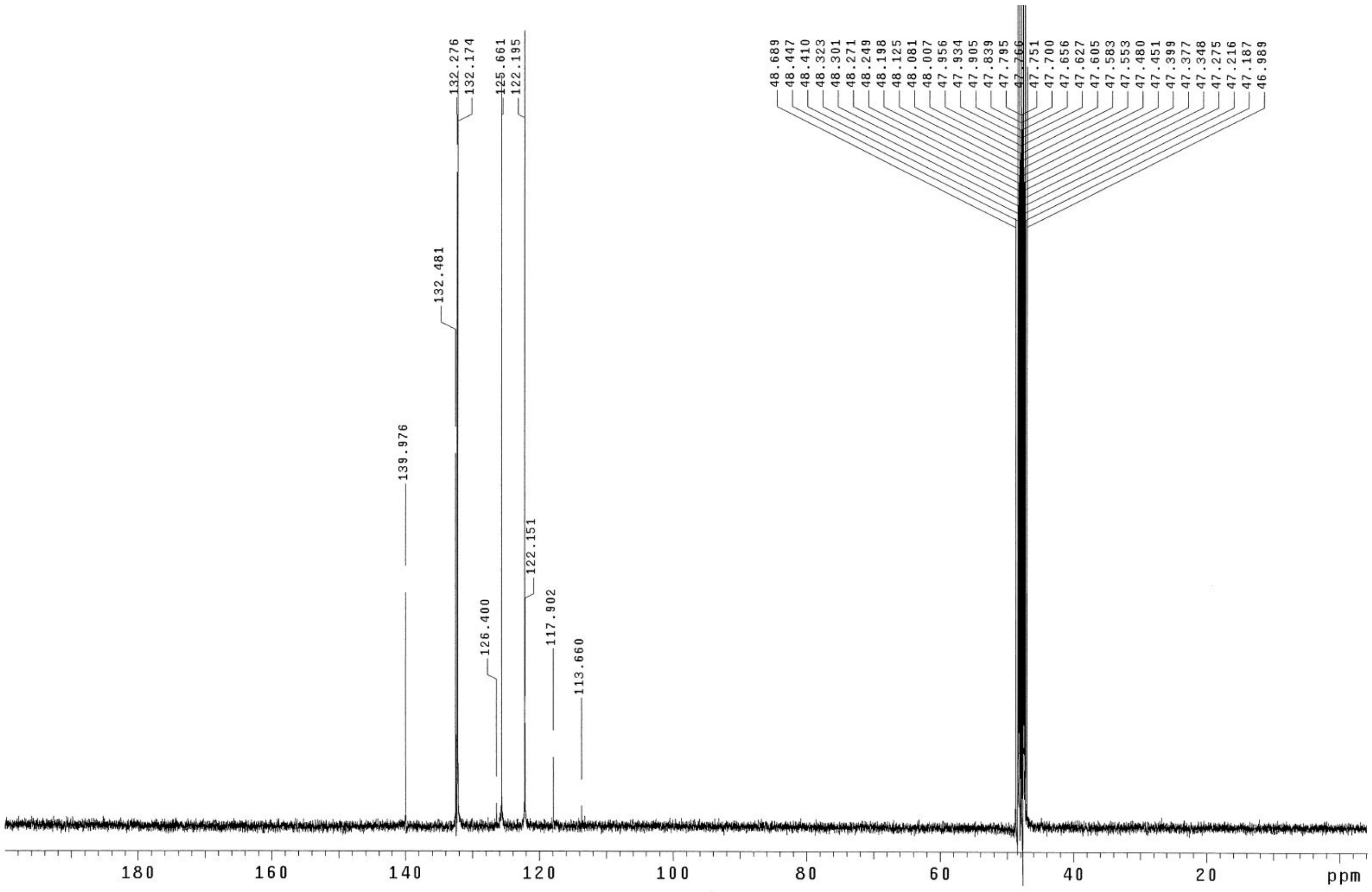

Figure S38. Compound 8d: ^{19}F NMR (376 MHz, CD_3CN)

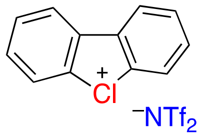

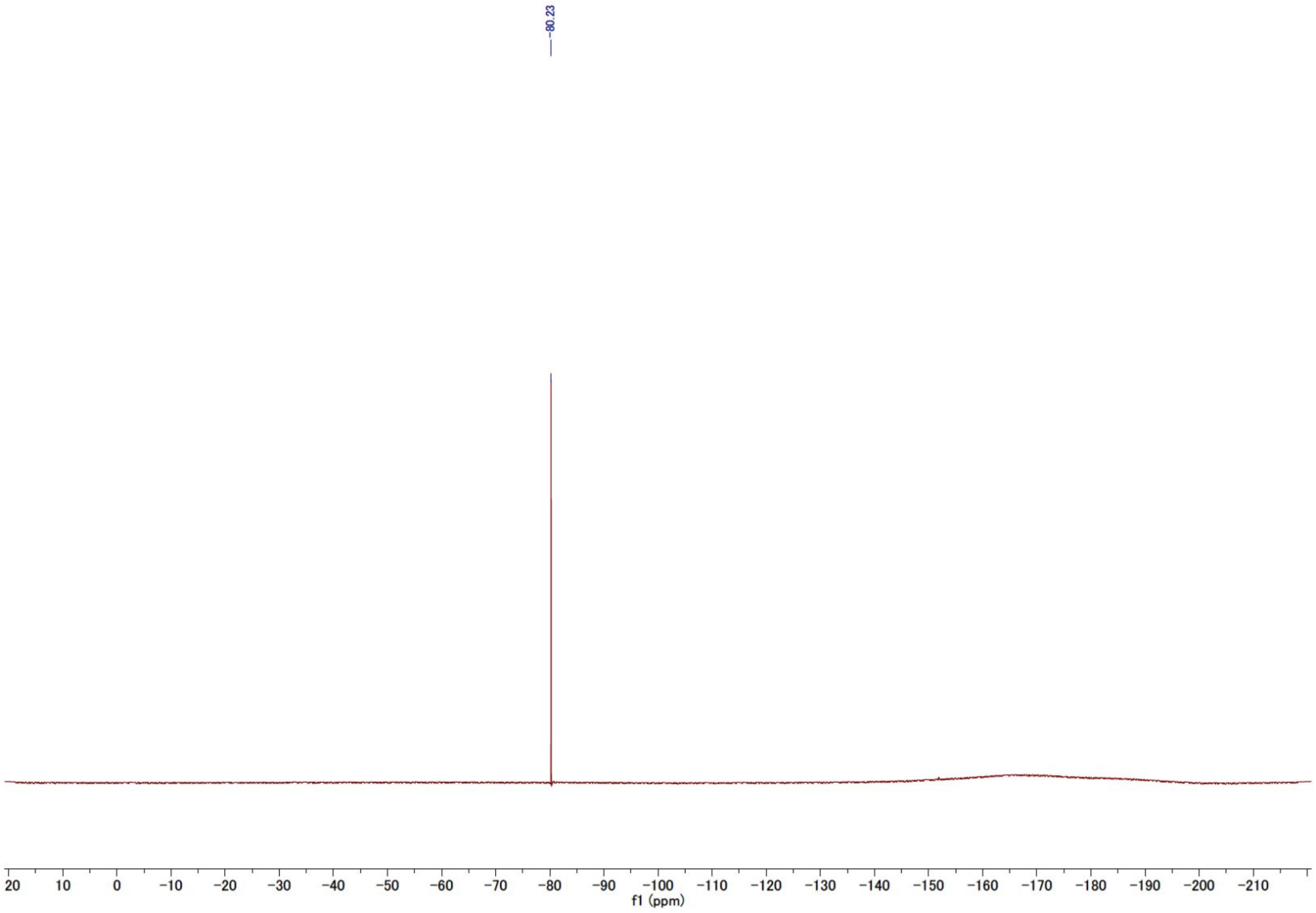

Figure S39. Compound 8e: ^{1}H NMR (400 MHz, CD_3CN)

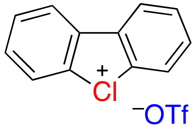

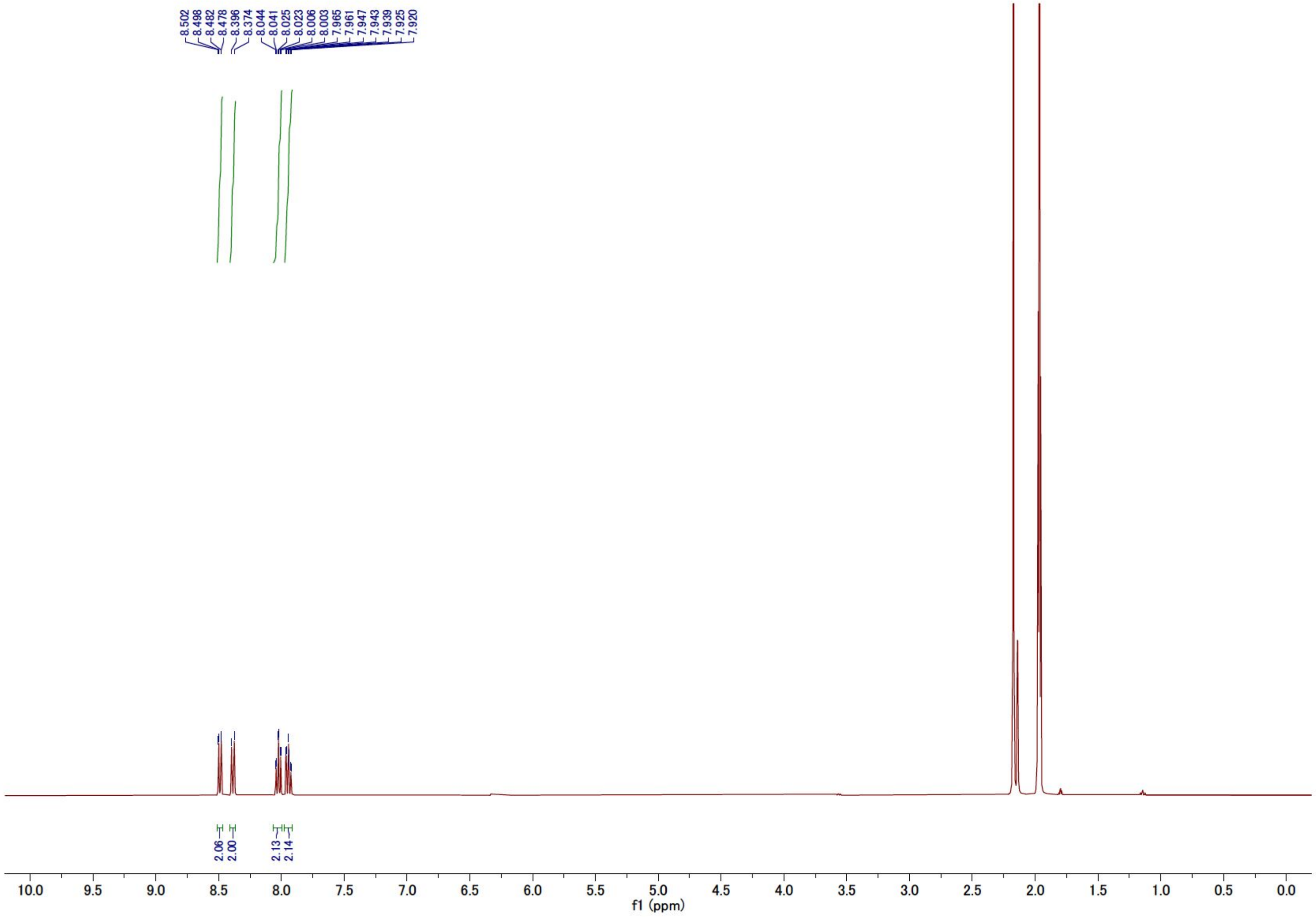

Figure S40. Compound 8e: ^{13}C NMR (100 MHz, CD_3CN)

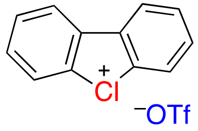

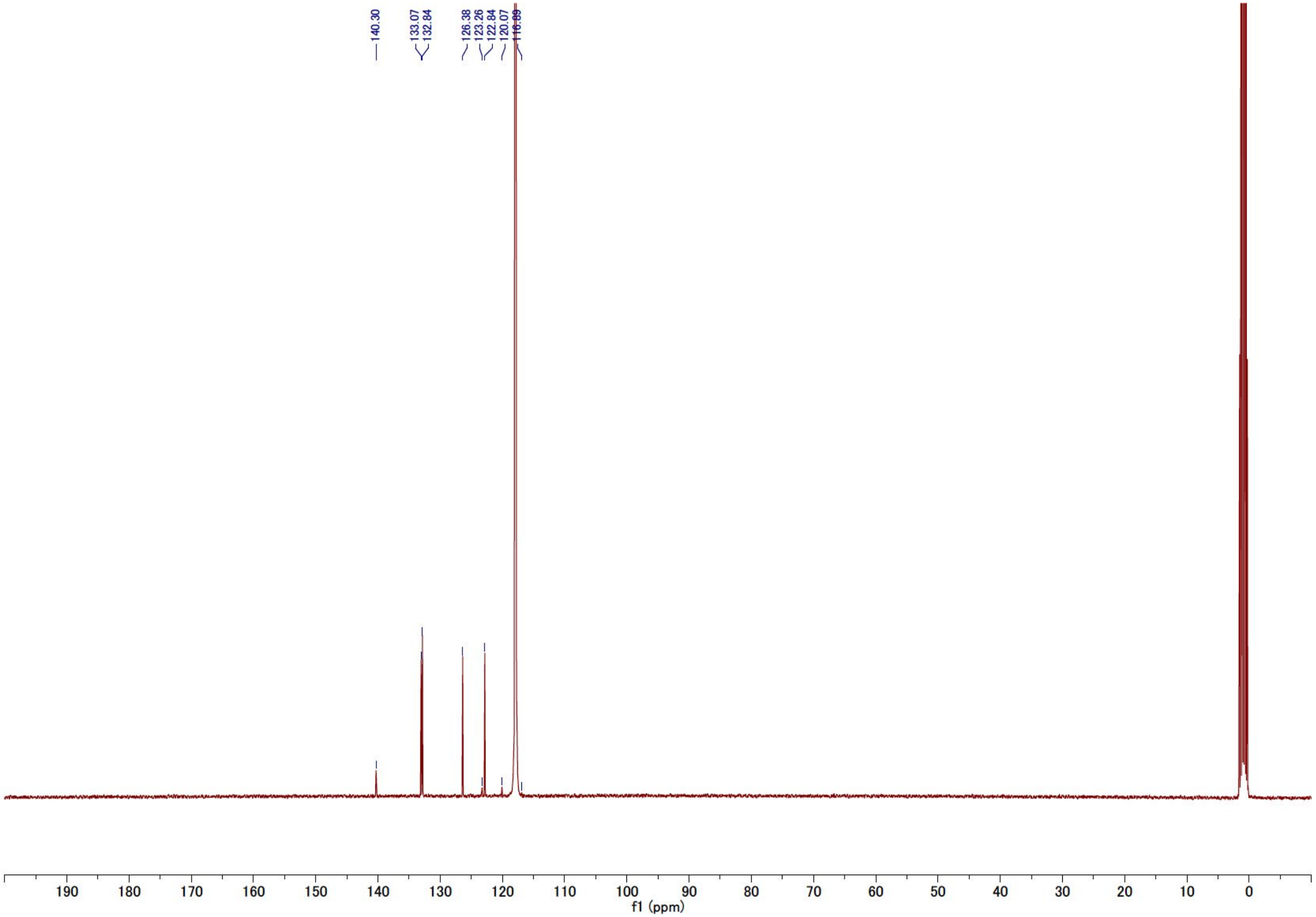

Figure S41. Compound 8e: ^{19}F NMR (376 MHz, CD_3CN)

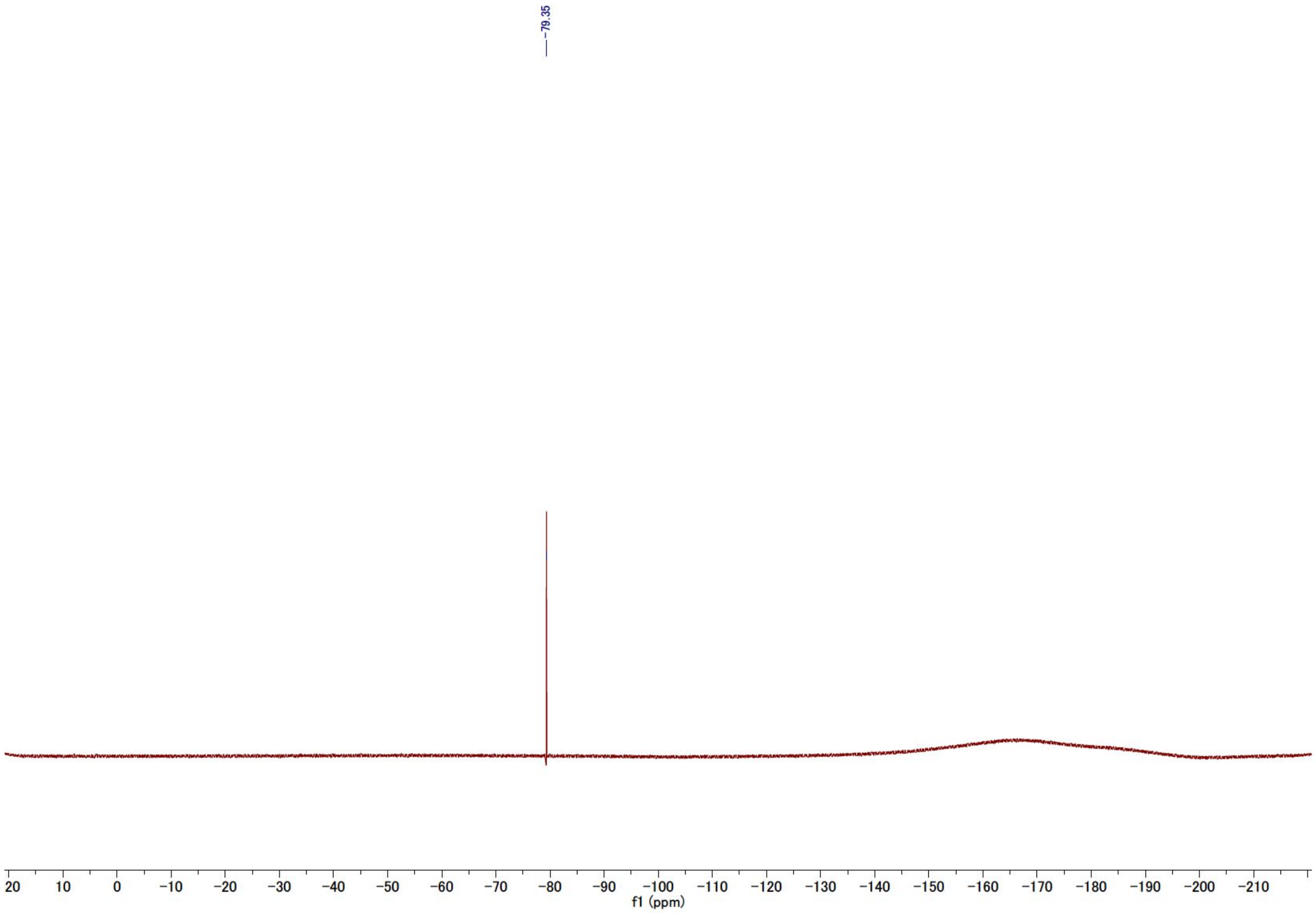

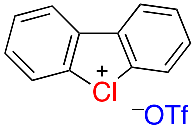

Figure S42. Compound 8f: ^{1}H NMR (400 MHz, CD_3OD)

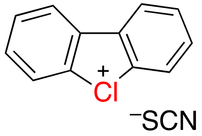

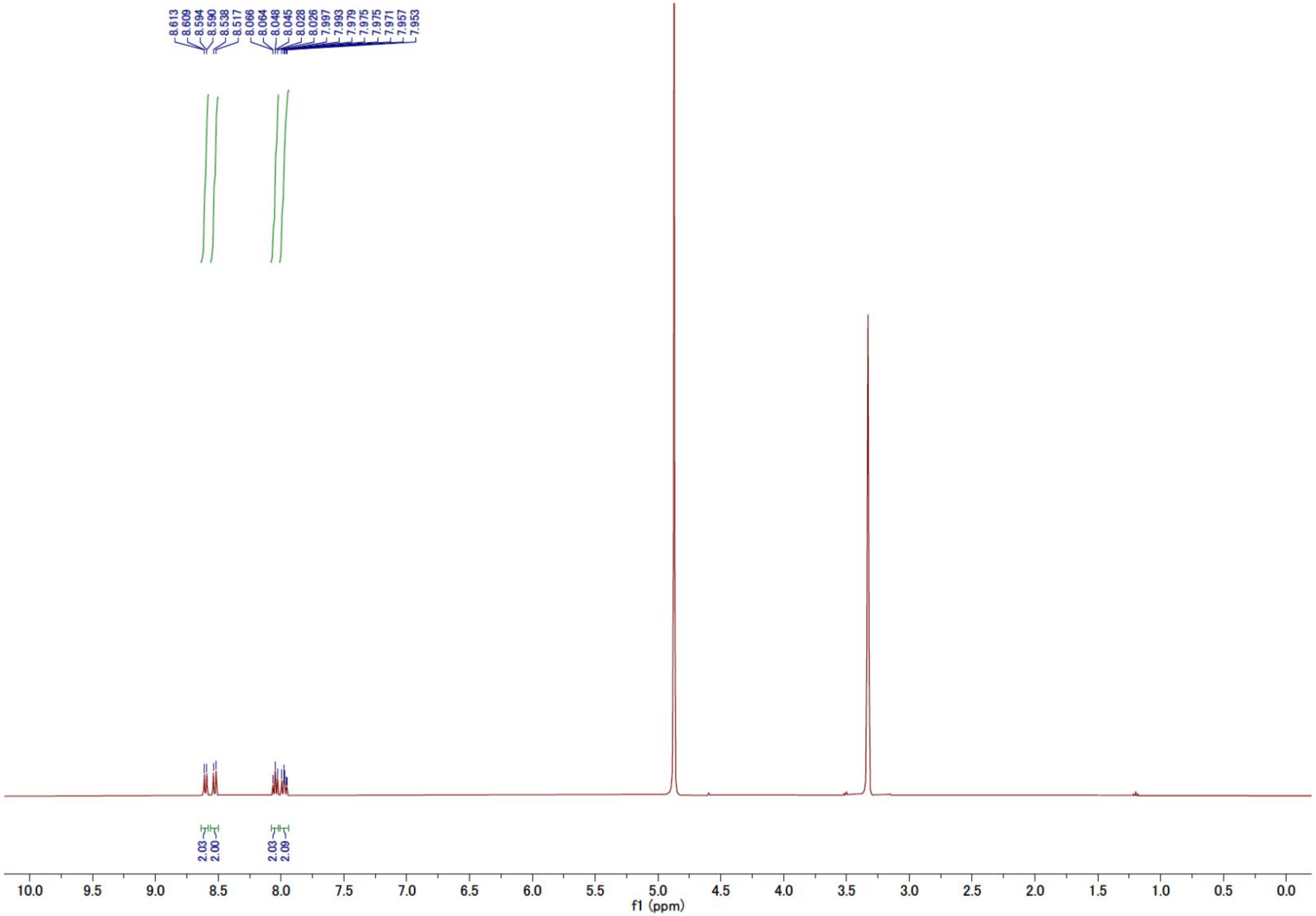

Figure S43. Compound 8f: ^{13}C NMR (100 MHz, CD_3OD)

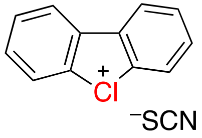

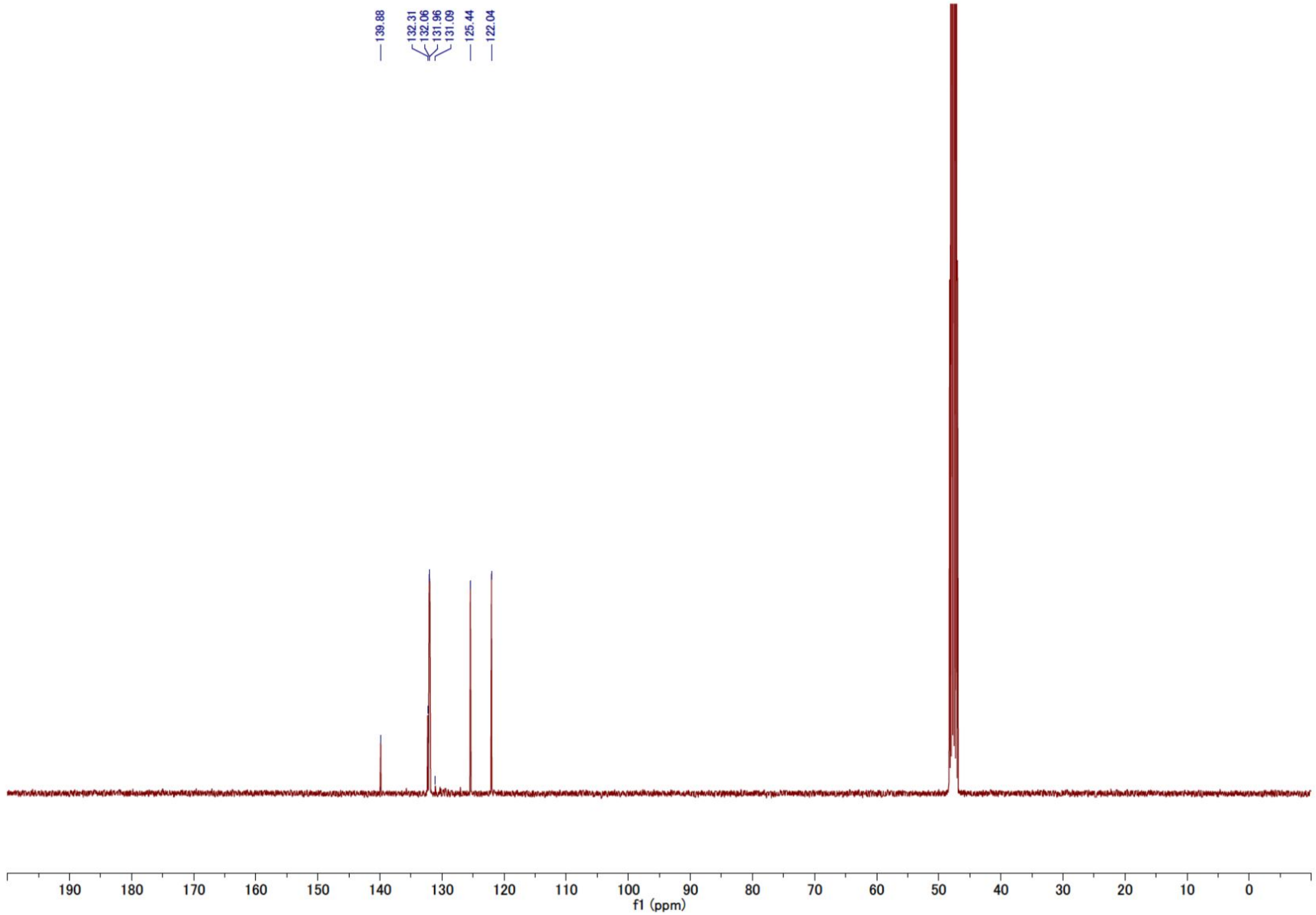

Supplement: Supplementary file 1 — ao3c07512_si_001.pdf [file ao3c07512_si_001.pdf]
